# Engagement, user satisfaction, and the amplification of divisive content on social media

**DOI:** 10.1093/pnasnexus/pgaf062

**Published:** 2025-03-05

**Authors:** Smitha Milli, Micah Carroll, Yike Wang, Sashrika Pandey, Sebastian Zhao, Anca D Dragan

**Affiliations:** Department of Computer Science, Cornell Tech, New York, NY 10044, USA; Department of Electrical Engineering and Computer Science, University of California, Berkeley, CA 94720, USA; Paul G. Allen School of Computer Science & Engineering, University of Washington, Seattle, WA 98195, USA; Department of Electrical Engineering and Computer Science, University of California, Berkeley, CA 94720, USA; Department of Electrical Engineering and Computer Science, University of California, Berkeley, CA 94720, USA; Department of Electrical Engineering and Computer Science, University of California, Berkeley, CA 94720, USA

**Keywords:** social media, ranking algorithms, stated preferences, user engagement, algorithmic audit

## Abstract

Social media ranking algorithms typically optimize for users’ revealed preferences, i.e. user engagement such as clicks, shares, and likes. Many have hypothesized that by focusing on users’ revealed preferences, these algorithms may exacerbate human behavioral biases. In a preregistered algorithmic audit, we found that, relative to a reverse-chronological baseline, Twitter’s engagement-based ranking algorithm amplifies emotionally charged, out-group hostile content that users say makes them feel worse about their political out-group. Furthermore, we find that users do *not* prefer the political tweets selected by the algorithm, suggesting that the engagement-based algorithm underperforms in satisfying users’ stated preferences. Finally, we explore the implications of an alternative approach that ranks content based on users’ stated preferences and find a reduction in angry, partisan, and out-group hostile content, but also a potential reinforcement of proattitudinal content. Overall, our findings suggest that greater integration of stated preferences into social media ranking algorithms could promote better online discourse, though potential trade-offs also warrant further investigation.

**Significance Statement**

The posts that users engage with on social media sometimes are not the ones that they value upon reflection. Should social media algorithms show users posts that they will engage with or posts that they explicitly say they want to see? We conduct an algorithmic audit of Twitter’s algorithm, which optimizes for what users will engage with, and find that it amplifies divisive content much more than if posts were ranked based on what users say they want to see. Our results suggest that focusing more on what users explicitly say they want could improve online discourse (though more research is needed into the potential trade-offs of this approach).

## Introduction

Social media ranking algorithms primarily personalize content to individual users by predicting what they will engage with (1, 2). For example, in April 2023, Twitter’s ranking algorithm was based on predicting whether a user would engage with a particular tweet using 10 different types of engagement (3). These predictions include behaviors like retweeting, replying, watching an embedded video, or lingering on a tweet for at least 2 min. Similarly, in December 2021, it was reported that TikTok’s algorithm predicted how long users would watch a video and whether they would like or comment on it (4). We refer to these kinds of ranking algorithms as *engagement-based* ranking algorithms. These algorithms can be seen as optimizing for users’ *revealed preferences*, i.e. they interpret a user engaging with a piece of content as an indication that the user wants to see that piece of content (5).

### Do engagement-based algorithms exploit human biases?

There is concern that by focusing on maximizing user engagement—especially reactive or passive behaviors like lingering—these algorithms may amplify content that is attention-grabbing but is unaligned with the user’s reflective values or with broader societal values (2, 6–10). For example, Brady et al. (7) suggest that, “content algorithms systematically exploit human social-learning biases because they are designed to optimize attentional capture and engagement time on the platform, and social-learning biases strongly predict what users will want to see.” They specifically claim that ranking algorithms tend to amplify what they term “PRIME” content—prestigious, in-group, moral, and emotional content. While humans may have developed biases toward PRIME information for adaptive reasons, they suggest that in the context of modern social media, exploiting these biases can lead to heightened social misperception and conflict. Similarly, Agan et al. (6) and Morewedge et al. (11) suggest that since cognitive biases are more likely to be triggered during fast, intuitive thinking (“system 1”), algorithms trained on more passive or impulsive behaviors—like time spent watching a video or lingering on a tweet—are likely to replicate these biases.

The notion that algorithms *exploit* implicit human biases to drive user engagement suggests that, if users deliberately chose the content they consumed, it would differ from what the algorithm prioritizes. However, most existing research does not clearly disentangle the algorithm’s exploitation of automatic human biases from deliberate human choices. For example, many studies do not control for who the user follows. On most social media platforms, the content that is shown to a user is heavily shaped by their own explicit choices of who to follow. Many existing studies find a correlation between the engagement that a post receives and characteristics such as its emotional intensity (12–14). However, it is possible that those posts received more engagement, not due to the algorithm prioritizing them, but because users chose to follow accounts that post more emotional content. Analogously, it was often hypothesized that YouTube’s recommendation algorithms led users down “rabbit holes” to politically extreme content (15–18). However, subsequent studies revealed that users often reach extreme channels through external links and subscriptions, suggesting that intentional human choice and demand might be an alternative explanation for the consumption of extreme content (16, 19–21).

Moreover, even controlling for who a user follows is not sufficient to disentangle deliberate choices from automatic biases. Most studies control for who a user follows by comparing posts selected by a ranking algorithm to those in a *reverse-chronological* baseline which consists of recent posts from the accounts a user follows. For example, in concurrent work, Bouchaud et al. (22) conducted such an audit of Twitter and found that the ranking algorithm selected more emotionally valent content compared to the reverse-chronological baseline. This implies that the ranking algorithm amplifies emotional content beyond what users’ deliberate choices of who to follow alone would generate, and therefore, provide stronger evidence of the algorithm exploiting human biases. However, such evidence would be far from conclusive. The algorithm might be surfacing more emotional content because, within the accounts a user follows, the user may actually prefer more emotional posts, even if they lack a direct mechanism to filter content at such a granular level. In other words, the algorithm could still be optimizing for what users would have deliberately selected on their own, given finer control over their content.

### Can optimizing for stated preferences lead to better outcomes?

Disentangling deliberate human choice from algorithmic exploitation of automatic biases is important not only for scientific understanding but also for designing better systems. If, as Brady et al. (7) suggest, algorithmic exploitation of social biases leads to greater social misperception and conflict, then redesigning algorithms to avoid such exploitation becomes imperative. In fact, a growing body of research in computer science is working toward this goal by trying to align recommender systems more to what users say they want to see—their *stated preferences*—rather than merely what they engage with—their revealed preferences. This is an interesting turn as conventional wisdom in fields such as economics (23, 24) often places higher priority on revealed preferences, as stated preferences might be unpredictive of behavior or affected by other factors like demand characteristics. On the other hand, if it is true that current ranking algorithms exploit automatic biases in users’ revealed preferences, then this suggests that we should explore alternatives that may provide a more reflective signal about users’ preferences (5, 8, 11, 25).

In any case, the choice between revealed and stated preferences is not an either-or. There is precedent for platforms optimizing some combination of both to yield benefits. Platforms currently get information on users’ stated preferences via *item-level surveys* (e.g. “How interested are you in this post?”) or *user controls* (e.g. clicking “Show Less” on an item) (2). In a recent workshop held on the use of these “nonengagement signals” that brought together 18 experts across seven major content-ranking platforms (2), the group came to consensus that (i) many platforms have built predictive models of item-level surveys and integrated them into their ranking algorithms, (ii) item-level surveys are typically correlated with integrity metrics, and (iii) using item-level surveys in ranking can improve user retention.

While promising, platforms’ current integrations of stated preferences are minimal and have not assuaged critics (8, 11, 25) as their algorithms still primarily focus on engagement. Indeed, studies have found that users often struggle to align recommendations with their stated preferences, even in the presence of user controls (26–28). Part of the problem is technical—survey responses and user controls are far less observed than engagement data, making them more difficult to predict and optimize (29, 30). However, recent research in computer science has aimed to develop more sophisticated methods to overcome these challenges (9, 29, 31), making stated preference optimization potentially much more practical in the future.

Despite increasing calls to move beyond behaviorism in recommender systems, there is limited empirical evidence for the potential of stated preference optimization to remedy problems with engagement optimization. To strengthen the empirical foundation of this emerging research area, we must demonstrate that users’ stated preferences offer a distinct signal compared to their revealed preferences. That is, we need to show that if users deliberately chose their content, it would significantly differ from the content that maximizes their engagement. This would also provide evidence for the theories that engagement-based algorithms exploit automatic biases (7, 8, 11). Additionally, we would need to show that then optimizing for stated preferences leads to better outcomes. It is possible that optimizing for stated preferences might worsen certain issues, e.g. users might choose to see more proattitudinal content, potentially reinforcing in-group bias. Our study aims to explore these questions.

### Hypotheses and research questions

We executed a preregistered algorithmic audit of Twitter^a^ that was designed to differentiate deliberate human choices from algorithmic exploitation of biases. Over 2 weeks in February 2023, we recruited a group of Twitter users (n=806) and collected (i) the first 10 tweets the *engagement-based* personalized ranking algorithm would have shown them and (ii) the 10 most recent tweets they would have seen from accounts that they follow, i.e. a *reverse-chronological* baseline.^b^ Informed consent was obtained from all participants. The study was approved by UC Berkeley’s IRB under the CPHS protocol ID number 2021-09-14618 and complies with all ethical regulations.

By also collecting users’ reverse-chronological timelines, we are able to assess the impact of the engagement-based timeline beyond users’ own deliberate decisions of who to follow. Moreover, beyond simply controlling for users’ following decisions, we also explicitly surveyed users for their stated preferences regarding each of the tweets shown in their engagement-based and reverse-chronological timelines (“When you use Twitter, do you want to be shown tweets like [@author-handle]’s tweet?”). This allowed us to test how distinct users’ revealed and stated preferences were. If users’ stated preferences do not sufficiently diverge from users’ revealed preferences, then we would expect to see that the engagement-based timeline, which optimizes for revealed preferences, also satisfies users’ stated preferences better than the reverse-chronological timeline.

In exploratory analysis, we also used these item-level survey responses to construct a counterfactual *stated preference timeline* consisting of the collected tweets that the user said they valued the most. We then compared this stated preference timeline with the engagement-based and reverse-chronological timelines, allowing us to assess how optimizing for stated preferences may affect the other outcomes that we studied (listed below), relative to the standard engagement-based or reverse-chronological timelines.

We preregistered five primary hypotheses and four secondary research questions. All hypotheses and research questions are stated as expectations about the effects of the engagement-based timeline relative to their reverse-chronological timeline. In exploratory analysis, we also analyzed the same outcomes for the stated preference timeline that we constructed using users’ explicit stated value for individual tweets.

Our first three hypotheses focused on the differences in the content selected by the engagement-based timeline and the reverse-chronological timeline. In particular, we hypothesized that, compared to the reverse-chronological timeline, the engagement-based timeline would show tweets (i) that were more emotional, (ii) had a stronger ideological leaning, and (iii) contained more expressions of animosity towards participants’ political out-group.

**Hypothesis 1.** The engagement-based timeline will show tweets that are more emotional (along four dimensions of anger, sadness, anxiety, and happiness), compared to the reverse-chronological timeline.
**Hypothesis 2.** The engagement-based timeline will show tweets with a stronger ideological leaning, compared to the reverse-chronological timeline.
**Hypothesis 3.** The engagement-based timeline will show tweets containing greater out-group animosity, compared to the reverse-chronological timeline.

These three hypotheses are consistent with both prior theoretical and empirical work. They are consistent with the theoretical framework put forth by Brady et al. (7), who suggest that social media ranking algorithms exploit humans’ social biases towards emotional, moralized, and in-group content. Moreover, previous observational studies have found that tweets with more emotion and out-group animosity tend to receive higher engagement (12, 14). However, as previously noted, these studies do not account for users’ choices in whom they follow, making it challenging to assess the specific influence of the ranking algorithm in amplifying emotional and out-group antagonistic content. Our hypotheses posit that engagement-driven algorithms do indeed amplify such emotionally charged, politically divisive content beyond the impact of users’ own follow choices.

We also had two hypotheses about how participants would feel after reading the tweets in their engagement-based timeline. The first was that, by selecting tweets with greater ideological leaning and out-group animosity, we expected readers to feel worse about their political out-group after reading tweets from their engagement-based timeline.

**Hypothesis 4.** Participants will feel worse about their political out-group after reading tweets from their engagement-based timeline, compared to the reverse-chronological timeline.

Our second reader-focused hypothesis was that participants would feel happier but less angry, sad, or anxious reading tweets in their engagement-based timeline. This contrasted with our expectation that the engagement-based timeline would increase the frequency of expressions of all four emotions (Hypothesis 1). The rationale for this difference is that platforms test ranking algorithms in A/B tests to identify those that optimize user retention (2); thus, we assumed that fostering positive emotions would encourage users to return to the platform. (Ultimately this turns out to be the only hypothesis we did not find supporting evidence for—readers reported feeling higher levels of all four emotions on the engagement-based timeline.)

**Hypothesis 5.** Participants will feel happier and less angry, anxious, or sad reading tweets in their personalized timeline, compared to the reverse-chronological timeline.

We also formulated four secondary preregistered research questions. The first two explored the emotional impact of the engagement-based timeline on both the content it curates (as in Hypothesis 1) and the emotions experienced by readers (as in Hypothesis 4), but focused specifically on political tweets. While we expected that the engagement-based timeline would increase the expression of all four measured emotions—anger, happiness, sadness, and anxiety—across tweets overall (Hypothesis 1), we were uncertain about the effects when considering only political tweets. It is possible that political tweets might primarily amplify negative emotions rather than emotional content more broadly, with a similar pattern reflected in the emotions experienced by readers.

**Question 1.** How angry, happy, sad, or anxious are the political tweets selected by the engagement-based timeline, compared to the reverse-chronological timeline?
**Question 2.** How happy angry, happy, sad, or anxious do readers feel after reading political tweets selected by the engagement-based timeline, compared to the reverse-chronological timeline?

Our next question related to the reader’s change in perception of their political in-group. We hypothesized that the engagement-based timeline would make the reader feel worse about their out-group (Hypothesis 4) because of increased exposure to tweets expressing animosity to the out-group. However, we were uncertain whether, conversely, the reader’s perception of their in-group would improve when exposed to tweets from the engagement-based timeline, especially because previous research has found that users are more likely to share tweets that are negative towards their out-group than those that are positive towards their in-group (32).

**Question 3.** How do readers’ perceptions of their political in-group change after reading tweets in their engagement-based timeline, compared to their reverse-chronological timeline?

Our final question investigated whether the engagement-based timeline aligns with users’ stated preferences—specifically, whether it shows them tweets they say they value. Users’ revealed preferences appear to favor the engagement-based timeline over the reverse-chronological one, as randomized experiments at Twitter have shown that it increases the amount of time users spend on the platform compared to the reverse-chronological timeline (30). However, theories suggesting that ranking algorithms exploit social biases (6, 7) suggest that users’ revealed preferences, which are given in an automatic state, may significantly differ from their stated preferences, which are obtained in a more reflective way, e.g. through user surveys.

**Question 4.** Do users see more tweets they (say they) value in the engagement-based timeline, compared to the reverse-chronological timeline?

## Results

From 2023 February 11 to February 27, we conducted our study on CloudResearch Connect, an online crowd-working platform. The study period was broken into four waves^c^ and participants could complete the study once during each wave. Every day, we recruited up to 150 eligible participants who lived in the United States, were at least 18 years old, and used Google Chrome. Furthermore, participants were required to use Twitter at least a few times a week and follow at least 50 people on Twitter (both gauged through self-reports). To collect data, participants were directed to download a Chrome extension that we developed which scraped their Twitter homepage to collect the top tweets from their personalized, engagement-based timeline. While scraping, the Chrome extension added an overlay to the homepage that prevented the user from seeing the tweets during collection. At the same time that the engagement-based timeline was collected, we queried the Twitter API to get the top tweets from the reverse-chronological timeline. Only public tweets were collected and no promoted tweets (advertisements) were collected.

After collecting both sets of tweets,^d^ participants were directed to complete a survey on Qualtrics that asked questions about each of the top 10 tweets from their engagement-based and reverse-chronological timeline.^e^ All tweets were displayed^f^ in a randomized order (thus, tweets from both timelines were typically interwoven rather than, for example, first showing all the engagement-based tweets and then all reverse-chronological tweets). If the same tweet was present in both the engagement-based and reverse-chronological timeline, then participants were only shown it once. If a tweet was a reply to another tweet, the user was shown both the replied tweet and the main tweet, and asked to answer the questions for both tweets. Similarly, if a tweet was a quote tweet, then the user was asked to answer the questions for both the quoted tweet and the main tweet.

For each tweet, we asked the user to assess whether it was about a political or social issue. If they marked the tweet as political, we asked them about the tweet’s ideological leaning, their own perception of their political in-group and out-group after reading the tweet, and whether the tweet author was expressing out-group animosity. For all tweets (including nonpolitical tweets), we asked users to assess the author’s emotions as well as the emotions the tweet made them feel, along four dimensions: happiness, anger, sadness, and anxiety. Finally, we also asked the reader for their stated preference about the tweet, i.e. whether they wanted to see tweets like it when they used Twitter. The full survey is provided in Section S5.

For our analysis of outcomes that are about the tweet itself (the tweet’s ideological leaning, the emotions expressed by the author, and whether the tweet expresses out-group animosity), as opposed to the reader’s emotions, we measure these outcomes in two ways. First, as just described, we collect readers’ perceptions of these outcomes. Second, we ask the same questions to GPT-4 (33), a large language model (LLM). The main text presents the results based on readers’ judgments, and Section S4.5 includes results using GPT-generated labels. In the main text, we present the results based on readers’ judgments, while Section S4.5 contains results using GPT-provided labels. The findings indicate that both sets of results align qualitatively, providing reassurance given the limitations of each approach. Reader-given labels have the disadvantage that the reader’s own background, e.g. political leaning, education, etc., might cause them to systematically misinterpret certain outcomes such as the author’s emotions (34). Conversely, while humans can grasp broader context beyond the tweet’s text, LLMs are limited to textual input and cannot process associated images or linked content. Moreover, LLMs have their own biases in responses that may not be reflective of the human population (35, 36). Nevertheless, the consistency between the two methods, each with their advantages and disadvantages, supports the robustness of our results.

A full description of our study procedure can be found in Section S1. In Section S4.1, we provide descriptive statistics of the metadata in both timelines, e.g. the number of likes, retweets, links, photos, etc. in each tweet, as well as analysis on the amplification of individual user accounts.

### Effects of engagement-based ranking

**Fig. 1.**
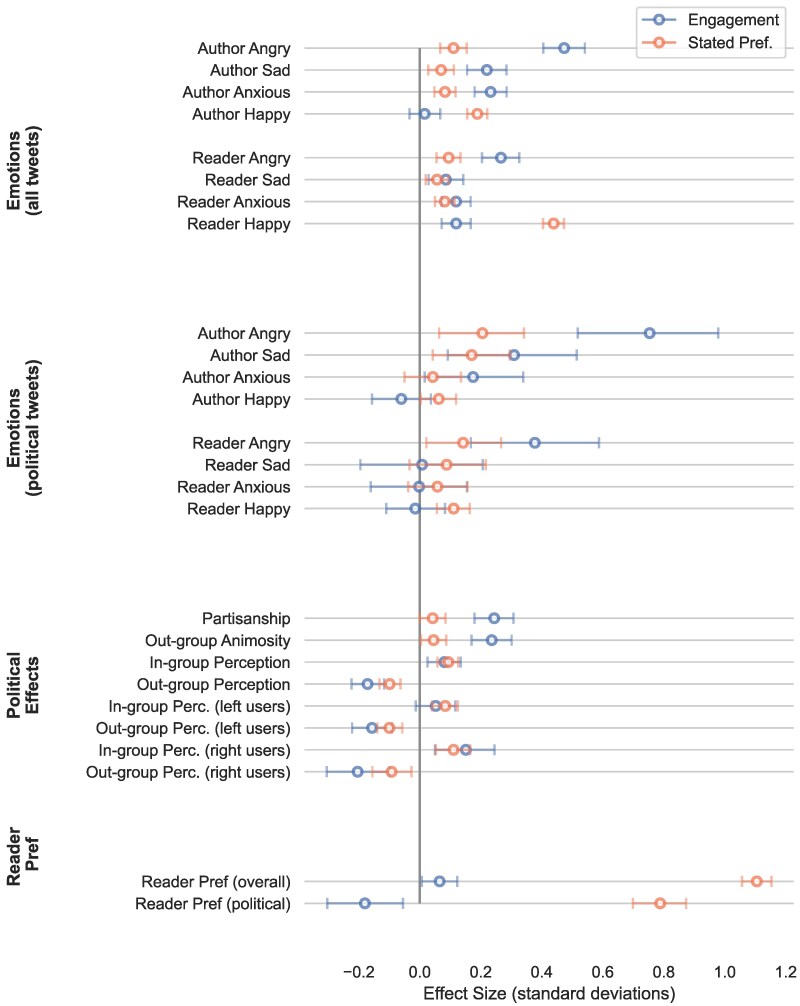
Average treatment effects for all outcomes. ATEs are shown with 95% bootstrap CI (unadjusted for multiple testing). The treatment effects of two different timelines are shown, relative to the reverse-chronological timeline: (i) Twitter’s own engagement-based timeline and (ii) our exploratory timeline that ranks based on users’ stated preferences. Exact values are provided in Tables S21 and S43. The outcomes shown here are based on reader judgments, the analogous effects for GPT-4 based labels can be found in Section S4.5 in Table S42 and Fig. S13. The effect sizes for both timelines are relative to the reverse-chronological timeline (the zero line). Average treatment effects are standardized using the SD of outcomes in the reverse-chronological timeline (see Section S1.3 for details).

First, we state our findings on the effects of Twitter’s engagement-based algorithm. All tested outcomes and our analysis plan were preregistered at https://osf.io/upw9a. Figure 1 shows a summary of the average treatment effect for each outcome. As specified in our preanalysis plan, the average treatment effect (of the engagement-based algorithm, relative to the control of the reverse-chronological ranking) is estimated through a difference in means (see Section S1.3), and two-sided *P*-values are estimated by paired permutation tests. In total, we tested 26 outcomes, and all results that are significant (at a *P*-value threshold of 0.05) remain significant at a false discovery rate (FDR) of 0.01. Thus, in expectation, none of our discoveries are false discoveries. The full table of standardized and unstandardized effect sizes, *P*-values, and FDR-adjusted *P*-values can be found in Section S3.

#### Political outcomes

We defined the *partisanship* of a tweet as the absolute value of the reader-provided ideological leaning of the tweet (ranging from −2 = “Far left” to +2 = “Far right”). Relative to the reverse-chronological baseline, we found that the engagement-based algorithm amplified tweets that exhibited greater partisanship (0.24 SD, P<0.001) and expressed more out-group animosity (0.24 SD, P<0.001). Furthermore, tweets from the engagement-based algorithm made users feel significantly worse about their political out-group (−0.17 SD, P<0.001) and better about their in-group (0.08 SD, P=0.0014). These effects remained significant when considering left and right-leaning users specifically, except for in-group perception in left-leaning users, where we found no significant effect.

#### Amplified emotionality

The engagement-based algorithm significantly amplified tweets that expressed negative emotions—anger (0.47 SD, P<0.001), sadness (0.22 SD, P<0.001), and anxiety (0.23 SD, P<0.001). It also led readers to feel more of all four emotions—anger (0.27 SD, P<0.001), sadness (0.09 SD, P=0.003), anxiety (0.12 SD, P<0.001), and happiness (0.12 SD, P<0.001). When considering only political tweets, we found that anger was by far the predominant emotion amplified by the engagement-based algorithm, both in terms of the emotions expressed by authors (0.75 SD, P<0.001) and the emotions felt by readers (0.37 SD, P<0.001).

#### User’s stated preference

For each tweet, we also asked users whether they wanted to see tweets like it when they used Twitter. We found that overall, tweets shown by the engagement-based algorithm are rated slightly higher (0.06 SD, P=0.022). Interestingly, however, the political tweets recommended by the engagement-based algorithm led to significantly lower user value than the political tweets in the reverse-chronological timeline (−0.18 SD, P=0.005).

### Ranking by stated preferences

Next, we conducted an exploratory analysis in which we simulated an alternative ranking of tweets that is based on users’ stated preferences. In particular, we took the approximately twenty unique tweets that each user was surveyed about and re-ranked them by the users’ stated preference for the tweet. Each tweet received a score of 1 = “Yes,” 0 = “Indifferent,” or −1 = “No,” depending on the users’ stated preference for the tweet.^g^ The “Stated Preference” (SP) timeline consisted of the 10 tweets (out of the approximately twenty unique tweets) that scored highest according to the users’ stated preferences (ties are broken at random). We chose the top 10 tweets to maintain consistency with the engagement-based and reverse-chronological timelines (where we also considered only the top 10 tweets).

**Fig. 2.**
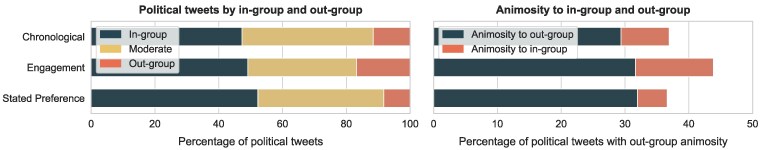
The distribution of political tweets and out-group animosity. The graph on the left shows the distribution of political tweets in each timeline, categorized by whether they align with the reader’s in-group, out-group, or are moderate. Meanwhile, the graph on the right delineates the proportion of political tweets that express out-group animosity, broken down by whether they target the reader’s in-group or out-group. The stated preference timeline has a lower percentage of tweets with animosity than the engagement timeline. However, this decrease is mainly due to a decrease in animosity towards the reader’s *in-group*; the percentage of tweets with animosity towards the reader’s *out-group* stays roughly the same.

As shown in Fig. 1, relative to the engagement timeline, the SP timeline reduced negativity (anger, sadness, anxiety) and increased happiness, both in terms of the emotions expressed by authors and the emotions felt by readers. Moreover, the content shown in the SP timeline is less partisan and less likely to contain expressions of out-group animosity. However, further analysis shown in Fig. 2 reveals that the reduction in partisanship and animosity in the SP timeline is due almost entirely to reducing the number of tweets from the reader’s out-group, and by reducing animosity directed toward the reader’s in-group (but not animosity towards the reader’s out-group).

## Discussion

###  

####  

##### The engagement-based algorithm does not cater to users’ stated preferences

For tweets overall, we found that users had only a slight preference for tweets in their engagement-based timeline compared to tweets in their reverse chronological timeline. Moreover, users were *less* likely to prefer the political tweets that the engagement-based algorithm selected. This is notable because, as measured by internal A/B tests at Twitter, the engagement-based algorithm causes users to spend more time on the platform, relative to the reverse-chronological timeline (30, 37). Thus, the engagement-based algorithm seems to be catering to users’ *revealed preferences* (in terms of engagement and usage patterns) but not to their *stated preferences*, particularly when it comes to political content. Rathje et al. (38) found that US adults believe social media platforms amplify negative, emotionally charged, and out-group hostile content but should not. This perspective may help explain why users were dissatisfied with the political tweets selected by the engagement-based algorithm, which tended to be angrier and more out-group hostile. The fact that the engagement-based algorithm is not aligned with users’ stated preference for tweets also suggests that its effects cannot entirely be explained by users’ deliberative choices.

##### The engagement-based algorithm amplifies partisan, emotional, out-group hostile tweets

We found that the engagement-based algorithm tends to select more emotionally charged, partisan, out-group hostile content than both the reverse-chronological timeline and the stated preference timeline. This finding helps clarify inconsistencies in previous research. Earlier studies relied on observational methods that were limited to specific subsets of content on the platform (12, 14) and replications produced mixed results (39, 40). These studies found a correlation between emotion and engagement but did not control for who users followed, leaving open the possibility that the observed correlation was due to users’ choices of who to follow. Our study provides insight into this debate by providing evidence that the engagement-based algorithm amplifies emotionally charged content, beyond users’ choices of who to follow, and even beyond what users’ stated preferences at the tweet-level would indicate. It also provides evidence for the theory of Brady et al. (7) who suggest that ranking algorithms exploit humans’ natural social biases towards moralized, emotional content.

##### The engagement-based timeline may be more polarizing than the reverse-chronological timeline

We also asked users how they felt about their political in-group and out-group (from “Much worse” to “Much better”) after each individual piece of content they saw from either the engagement-based or reverse-chronological timeline. We found that, after reading tweets selected by the engagement-based algorithm, users tended to have more positive perceptions of their in-group and more negative perceptions of their out-group, compared to the reverse-chronological timeline. Our results suggest that the engagement-based ranking algorithm selects more polarizing content compared to what would be expected from users’ following choices alone.

However, it is unclear whether ranking algorithms have lasting effects on users’ attitudes and affective polarization. In contrast to our content-specific surveying, Guess et al. (2023) measured the effect of the Facebook and Instagram ranking algorithms on users’ affective polarization, in general, between two weeks to three months after the start of their experiment (41). They found no significant impact on users’ affective polarization. Crucially, however, the scope of Guess et al. (2023)’s research does not encompass “general equilibrium” effects, such as the possibility for ranking algorithms to indirectly shape user attitudes by incentivizing and changing the type of content that users produce in the first place.

##### Ranking by stated preferences may reinforce in-group bias

We also found that while the stated preference timeline had fewer partisan tweets than the engagement-based timeline, this was primarily due to a reduction in tweets from the reader’s political out-group. Similarly, the lower out-group animosity in the stated preference timeline (relative to the engagement-based timeline) was primarily due to reducing hostility towards the readers’ in-group (but not towards their out-group). And we did not find a significant difference between the engagement-based timeline and the stated preference timeline, when it came to effects on readers’ perceptions of their in-group and out-group. Overall, these results suggests that it is possible that ranking by stated preferences could *heighten* users’ exposure to content that reinforces their preexisting beliefs, relative to the engagement-based algorithm. This is in line with prior research suggesting that users’ preference for proattitudinal political content and sources is a key factor contributing to greater exposure to in-group content compared to out-group content (42–44).

Our findings contrast with that of Agan et al. (6). They posit that algorithms that learn from users’ reflexive engagement tend to replicate automatic “system 1” biases. As supporting evidence, they show that, on Facebook, ranking content based on engagement increases exposure to in-group content (where in-groups are defined by race or religion), whereas ranking by stated preferences reduces this exposure. However, our results reveal an opposite trend when defining in-groups and out-groups by political affiliation. This might indicate that political in-group preferences are not solely “system 1” biases but also involve more deliberate “system 2” preferences. This distinction between political out-groups and other societal out-groups may also be an important distinction for the theory proposed by Brady et al. (7), which also posits that ranking algorithms capitalize on humans’ social biases towards in-group favoritism.

An important caveat in interpreting our results is that we only had a pool of approximately twenty tweets—the top 10 the users’ chronological and engagement timelines—to choose from when ranking by stated preferences. The platform has access to a much larger pool of tweets, and more research is needed to understand the impact of ranking by stated preferences in such a context. For example, users might be open to seeing respectful and civil content from their political out-group, but those tweets may not have been common in our limited pool of tweets—especially since about half were explicitly selected using engagement metrics.

It also may be that variants of the stated preference timeline can avoid reinforcing in-group bias. In Section S4.8, we investigate a slight modification to the stated preference timeline, one which involves tie-breaking content ranking based on the presence of out-group animosity, rather than random tie-breaking. This approach is similar to that of Jia et al. (45) who suggest ranking based on “societal objective functions,” e.g. an objective that prioritizes content with prodemocratic attitudes. Compared to the engagement-based and reverse-chronological timeline, we find that this modified SP timeline greatly reduces the amplification of out-group hostile content while avoiding heightened exposure to in-group content (Table S44 and Figs. S46–S47).

##### Implications on algorithm design

Many have advocated for greater incorporation of users’ stated preferences into content ranking algorithms (5, 8, 11). Typically, these suggestions do not aim to completely discard engagement data, which remain a valuable signal about users’ interests. Rather, the aim is to mitigate the biases associated with user engagement by placing greater emphasis on users’ stated preferences than is currently practiced. However, as of yet, there is still little empirical evidence showing that such a shift could remedy some of the problems with engagement optimization. Our study helps address this gap by showing that, relative to engagement-based ranking, optimizing for stated preferences can decrease the prominence of emotionally charged, divisive content—content that others have argued can lead to greater social misperception and conflict (7). These findings suggest that more emphasis on stated preferences could improve online discourse, though more research is also needed into potential trade-offs such as the reinforcement of in-group bias discussed earlier.

Although our findings are promising, there are still challenges to overcome before implementing more stated preference optimization on real-world platforms. The first is technical. Many platforms already incorporate users’ stated preferences (based on user surveys or controls like a “Show less” button) to some extent (2). However, these stated preferences are rarely observed and harder to optimize than engagement. As a result, engagement still remains the dominant factor in ranking decisions. Nonetheless, a growing body of work in computer science is exploring more effective methods for incorporating stated preference signals (9, 29, 31), making it possible that it will be increasingly feasible to optimize for stated preferences going forward.

The second challenge is platform incentives. Social media companies typically operate on an advertising-based revenue model, and thus, are incentivized to keep users on their platform for longer. Therefore, it may be challenging to prioritize stated preferences if doing so decreases user retention or time spent on the platform. Nevertheless, there are some reasons to be optimistic. First, stated preferences would complement rather than replace engagement. It may be possible to find a balance of both that improves societal outcomes without harming business metrics. Indeed, there is some evidence that such a balance is possible. Some companies already incorporate stated preferences, to some extent, in ranking and have found beneficial results on retention (2). A recent study by Piccardi et al. (46) also found that down-ranking posts on Twitter with high out-group animosity—content that users in our study often reported not wanting to see—did not reduce the amount that users engaged with posts through liking and sharing. These initial results are far from conclusive, however, and more investigation is needed to understand the impact of stated preference optimization on business metrics.

### Limitations

There are several limitations to our study that should be regarded when interpreting our results. First, our study analyzed the difference in users’ engagement-based and reverse-chronological timelines during only one point in time. Such an approach does not capture the engagement-based algorithm’s long-term effects. For example, by incentivizing some types of content over others, the algorithm changes what type of content is produced in the first place, which, in turn, affects what is available in the reverse-chronological timeline. If users have learned to produce more of the content that the engagement-based algorithm incentivizes (7, 47), then its long-term effects on the content-based outcomes we measure (emotions, partisanship, and out-group animosity in tweets) may be even greater. On the other hand, other research has failed to find long-term changes in user beliefs due to social media ranking algorithms (41, 48–50), and the short-term changes in users’ perceptions of their in-group and out-group that we found may not persist. Similarly, it is difficult to ascertain what the second-order effects of a stated preference feed might be. Rigorous experimentation needs to be done to establish how all the effects found in our study change over time in response to feedback loops (51).

Second, we required participants to install a Chrome extension and relied on an online crowd-working platform for participant recruitment, and this may have impacted the types of participants we could attract. In particular, compared to Twitter users in the 2020 ANES study (52), our population tended to be younger (53% of our study were aged 18–34 years old, compared to 33% in the ANES study) and more likely to affiliate with the Democratic Party (56% Democrat in our study vs. 43% in the ANES study) (see Section S2, Tables S3–S20 for full demographic statistics). We report on heterogenous effects by different demographic groups in Section S4.6 (Figs. S14–S45), however, more research may be warranted before generalizing to the full Twitter population.

Another concern pertains to the ecological validity of our study. We surveyed participants about each of the first 10 tweets in each timeline, a method that deviates from real-world Twitter usage where users can scroll past various tweets. Looking at the first 10 tweets might be a reasonable approximation though—Bouchaud et al. (2023) found that users viewed an average of 30 tweets in one session of using Twitter (22). In Section S4.4, we show that our results are robust to using a range of thresholds, spanning from the first five tweets to the first 10 tweets (Figs. S10–S12). Still, the effects of real-world social media use may not be accurately captured by averaging the impact of tweets shown to users. For instance, users may only be influenced by a few particularly notable tweets as they scroll through their timeline.

There is also the possibility that respondents experienced survey fatigue, as we repeatedly asked them about twenty tweets. To address this, we included attention checks in the survey and excluded participants who failed these checks. Nonetheless, we cannot completely rule out the effect of fatigue on the study’s results. Additionally, when evaluating certain tweet-related outcomes (e.g. perceived partisanship or emotional tone) based on reader perception, participants’ own backgrounds is likely to influence their preceptions (34). Although we conducted parallel analyses using GPT-4 to label these outcomes (see Section S4.5), LLMs have their own biases (35, 36) and are limited to analyzing only the textual content of the tweets, excluding images, videos, and linked websites. The similar results from both GPT-4 and reader assessments support the robustness of our findings. Still, it is essential to consider the complementary biases from both readers and GPT-4 when interpreting both sets of results.

Finally, throughout this article, we frequently discuss disentangling the effects of ranking algorithms from intentional human decisions such as which accounts to follow. However, over a longer period, ranking algorithms also shape these choices since users are more inclined to follow accounts that appear in their timelines. Consequently, even when using an engagement-based timeline, users’ follow decisions—shaped by the engagement-based algorithm’s recommendations—can gradually influence their reverse-chronological timeline, which is based on their followed accounts. This means that, over time, the engagement-based algorithm indirectly affects the content of the reverse-chronological timeline. Our study does not account for the potential long-term impact of the engagement-based algorithm on users’ choices of who to follow.

## Supplementary Material

pgaf062_Supplementary_Data

## Data Availability

The data and code needed to reproduce our results are available to the research community at http://github.com/smilli/twitter.

## Notes

a Twitter rebranded to “X” in July 2023. Our study took place before the rebranding, so we refer to the platform as “Twitter.”

b On Twitter, users can opt out of the engagement-based timeline and use the reverse-chronological baseline instead, making it an especially relevant baseline to test against

c The time periods for the waves (inclusive) were February 11–14, February 16–19, February 21–23, and February 25–27. Table S1 shows the distribution of the number of waves that participants took part in, and Table S2 details attrition rates across waves.

d See Sections S4.1 and S4.2 for descriptive statistics and information about the amplification of individual accounts (Tables S22–S24 and Figs. S2 and S3).

e See Section S4.3 for the distribution of responses to survey questions (Tables S25–S41 and Figs. S4–S9).

f Figure S1 shows an example of how tweets were displayed to participants.

g If a tweet was a quote tweet or a reply to another tweet, then we combined the two tweets into one “tweet object” and score it by the average of the users’ stated preferences for both tweets.
