# Supplementary material for: Engagement, user satisfaction, and the amplification of divisive content on social media
Source: PNAS Nexus. 2025 Mar 5;4(3):pgaf062. doi: 10.1093/pnasnexus/pgaf062 (PMC11894805; doi:10.1093/pnasnexus/pgaf062)
Supplement: pgaf062_Supplementary_Data [file pgaf062_supplementary_data.pdf]

Supplementary Materials

Table of Contents

S1 Materials and methods **20**

S1.1 Study procedure 20

S1.2 Pre-registration and deviations 22

S1.3 Estimation of average treatment effects 23

S2 User-level survey questions and demographics **25**

S2.1 Demographics of our study 25

S2.2 Demographics of Twitter users in ANES 2020 study 28

S3 Pre-registered analysis **31**

S4 Exploratory analysis **32**

S4.1 Descriptive statistics: tweet metadata 32

S4.2 Amplification of individual accounts 32

S4.3 Distribution of responses to survey questions 37

S4.4 Effects of Engagement Timeline with Varied Tweet Threshold 44

S4.5 Effects of engagement timeline with GPT-4 labels 46

S4.6 Heterogeneous effects 51

S4.7 Effects of stated preference timeline 74

S4.8 Effects of SP-OA timeline 75

S5 Survey questionnaires **78**

S1 Materials and methods

S1.1 Study procedure

We conducted our study between February 11 to February 27, 2023 on CloudResearch Connect[8], a crowd-working platform. Informed consent was obtained from all participants. The study was approved by UC Berkeley's IRB under the CPHS protocol ID number 2021-09-14618 and complies with all ethical regulations. Moreover, at the end of the study period, our rating (given by study participants) on CloudResearch was 4.9 stars, higher than 99 percent of other researchers on the platform.

The study period was broken into four waves and participants could complete the study once during each wave.[9] The time periods for the waves (inclusive) were 02/11-02/14, 02/16-02/19, 02/21-02/23, and 02/25-02/27. Every day, we recruited up to 150 eligible participants who lived in the United States, were at least 18 years old, and used Google Chrome. Furthermore, participants were required to use Twitter at least a few times a week and follow at least 50 people on Twitter (both gauged through self-reports). To collect data, participants were directed to download a Chrome extension that we developed which scraped their Twitter homepage to collect the top tweets from their personalized timeline. While scraping, the Chrome extension added an overlay to the homepage that prevented the user from seeing the tweets during collection. At the same time that the personalized tweets were collected, we queried the Twitter API to get the top tweets from the chronological timeline. Only public tweets were collected and no promoted tweets (advertisements) were collected.

After collecting both sets of tweets, participants were directed to complete a survey on Qualtrics that asked questions about each of the top ten tweets from their personalized and chronological timeline. All tweets are displayed in a randomized order (thus, tweets from both timelines are typically interwoven rather than, for example, first showing all the personalized and then all chronological tweets). If the same tweet was present in both the personalized and chronological timeline, then participants were only shown it once. Out of the 10 tweets in both timelines, on average, 2.35 of these tweets were common to both of the timelines. On each question, the tweet is displayed for reference as an embedded tweet, so it looks as similar as possible to the way it would on Twitter. If a tweet is a reply to another tweet, the user is shown both the replied tweet and the main tweet, and asked to answer the questions for both tweets. Similarly, if a tweet is a quote tweet, then the user is asked to answer the questions for both the quoted tweet and the main tweet.

The outcomes we measured concerned the emotions expressed by the author (on four axes: anger, sadness, anxiety, and happiness), the reader emotions (on four axes: anger, sadness, anxiety, and happiness), the author's expression of out-group animosity, the partisan leaning of the tweet, the readers' in-group and out-group perception after reading the tweet, and readers' explicit preference for the tweet. The full survey is provided in SM section S5, but the question phrasings and their possible answers are provided below for quicker reference:

- **Author emotions questions.** Participants are asked: "How is [@author-handle] feeling in their tweet?" For each emotion (angry, anxious, happy, sad), the participant responds on a Likert scale

[8]Prior academic research has found that participants recruited on CloudResearch or Prolific tend to provide higher-quality data than those recruited from Mechanical Turk or Qualtrics [23, 26]. We selected CloudResearch over Prolific because of its functionality that enables requesting that participants download a Chrome extension. CloudResearch enforces one account per participant, verifies that each IP address aligns with the reported location, and ensures that the bank or PayPal accounts for cashing out are unique to each participant. Participants are only allowed to sign up for the platform if they pass an onboarding process that checks for properties such as attention, language comprehension, honesty, and their ability to follow instructions. To maintain high data quality, CloudResearch continually monitors participants, using random attention checks and investigating those who are frequently rejected or flagged by researchers [32].

[9]To prevent repeat participation, we ensured that each Twitter user ID could only be used once per wave. For a participant to participate multiple times in a single wave, they would have needed to create multiple Twitter accounts and bypass CloudResearch's safeguards to register multiple CloudResearch Connect accounts.

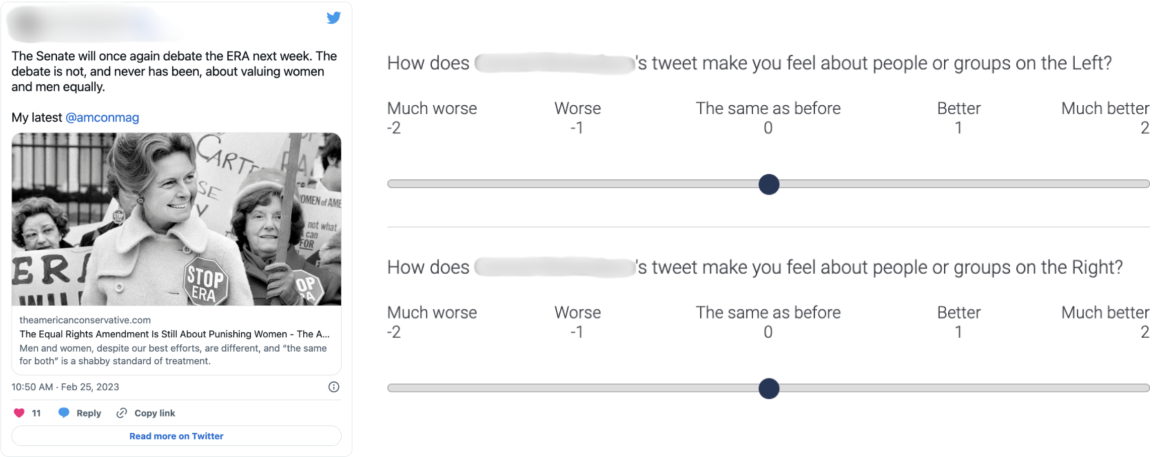

Figure S1: Sample view of the survey: users saw tweets embedded alongside each question for reference. The user's Twitter display name and username are blurred here but were not blurred to study participants. The photograph included in the tweet is in the public domain.

of "Not at all," "Slightly," "Somewhat," "Moderately," or "Extremely."

- **Reader emotions.** Participants are asked: "How did [@author-handle]'s tweet make you feel?" For each emotion (angry, anxious, happy, sad), the participant responds on a Likert scale of "Not at all," "Slightly," "Somewhat," "Moderately," or "Extremely."
- **Is tweet political.** Participants are asked: "Is [@author-handle]'s tweet about a political or social issue?" with a binary response of "Yes" or "No"
- **Political leaning.** Participants are asked: "How does [@author-handle]'s tweet lean politically?" Participants respond on a Likert scale of "Far left," "Left," "Moderate," "Right," or "Far right."
- **Political affect towards Left.** Participants are asked: "How does [@author-handle]'s tweet make you feel about people or groups on the Left?" Participants respond on a Likert scale of "Much worse," "Worse," "The same as before," "Better,", or "Much better."
- **Political affect towards Right.** Participants are asked: "How does [@author-handle]'s tweet make you feel about people or groups on the Right?" Participants respond on a Likert scale of "Much worse," "Worse," "The same as before,", "Better," or "Much better,"
- **Out-group animosity towards the Left.** Participants are asked: "Is [@author-handle]'s tweet expressing anger, frustration, or hostility towards a person or group on the Left?" Participants choose between a response of "Yes" or "No" This question is only asked if political leaning is "Right" or "Far right."
- **Out-group animosity towards the Right.** Participants are asked: "Is [@author-handle]'s tweet expressing anger, frustration, or hostility towards a person or group on the Right?" Participants choose between a response of "Yes" or "No." This question is only asked if political leaning is "Left" or "Far left."
- **Value.** Participants are asked: "When you use Twitter, do you want to be shown tweets like [@author-handle]'s tweet?" Participants choose between "No," "Indifferent," "Yes."

Data exclusions. Those who passed the pre-screen but did not complete data collection with the

Times Participant Completed Survey	Number of Unique Participants
1	359
2	139
3	139
4	169
**Total**	**806**

Table S1: The table displays the distribution of the number of times that each participant took part in the study. Most participants only participated once, but many participated two or more times.

Chrome extension were paid $0.75, while those who completed data collection and the full survey (which takes approximately 30 minutes) were paid $10 (a rate of $20/hr). Overall, 18 percent of participants who consented to the experiment did not complete data collection with the Chrome extension. This was either because they chose to not use the Chrome extension or ran into an error during data collection. Since the Chrome extension relies on scraping Twitter's homepage to retrieve tweets it may not work if, for example, the user has conflicting Chrome extensions or is in a Twitter A/B test that changes the UI of the Twitter homepage. Furthermore, we also surveyed participants about two attention-check tweets (that changed during each of the four waves) that were unambiguously either left-leaning or right-leaning. If a participant's survey response did not pass the attention checks, it was excluded from the final data set used for analysis. Furthermore, if a participant did not successfully complete the survey (e.g. did not complete data collection or pass the attention checks) in one wave, then they were excluded from participating in future waves. Our final data set consisted of 1730 responses from 806 unique participants. Table S2 shows statistics on the attrition of participants from consenting to passing the attention checks.

S1.2 Pre-registration and deviations

All our hypotheses, outcome measures, and statistical analyses were pre-registered on Open Science Framework (OSF) at https://osf.io/upw9a. However, we made the following deviations from the pre-registration. First, we opted to use CloudResearch Connect instead of CloudResearch's Mechanical Turk toolbelt for participant recruitment because CloudResearch Connect had features better suited for our study, such as the ability to flag our study as requiring a software download. Second, due to limitations in the number of unique Twitter users we could recruit on CloudResearch Connect, we divided our data collection period into four intervals and allowed participants to complete the study once in each interval. Third, we underestimated the study's costs, resulting in us stopping data collection when we reached our budget (at around 1700 pairs of timelines) instead of our initial target of 2000 pairs of timelines. And finally, we asked participants to rate out-group animosity on a five-point Likert scale from "Not at all" to "Extremely" for main tweets but accidentally left the response options for quoted and replied tweets as binary "Yes" or "No" options. Thus, for analysis, we collapse the Likert responses to a binary scale where "Not at all" corresponds to "No" and anything higher corresponds to "Yes."

Wave	Consented	Collected Data	Completed Survey	Passed Attention Check
**1**	816	599	586	508
**2**	688	585	574	454
**3**	514	440	434	405
**4**	486	441	432	363
**Total**	**2504**	**2065**	**2026**	**1730**

Table S2: The table displays, for each study period, the number of participants who consented to the study, successfully completed the data collection through the Chrome extension, completed the survey, and passed the attention check.

S1.3 Estimation of average treatment effects

As specified in our pre-registered analysis plan, our main estimation of the average treatment effect is through a simple difference in mean outcomes between the personalized and chronological timelines. Let $\mathcal{U} = \{1, 2, \dots, n\}$ be the set of participants and $\mathcal{W}_u \subseteq \{1, 2, 3, 4\}$ be the set of study waves that a user $u \in \mathcal{U}$ participates in. During study wave $w \in \mathcal{W}_u$, participant u rates both a set of tweets $\mathcal{T}_{w,u}(1)$ from their engagement-based timeline (denoted as timeline 1) and a set of tweets $\mathcal{T}_{w,u}(0)$ from their chronological timeline (denoted as timeline 0). The number of tweets that each participant rates is approximately ten from each timeline, i.e., $|\mathcal{T}_{w,u}(1)| \approx 10$ and $|\mathcal{T}_{w,u}(0)| \approx 10$. However, this number can be higher if a timeline contains quote tweets or replies. For both quote tweets and replies, participants rate two tweets: the quote tweet and the tweet being quoted or the reply and the tweet being replied to.

Let $Y_{w,u,t,q}(x)$ be the response of participant u to question q on tweet $t \in \mathcal{T}_{w,u}(x)$ from timeline $x \in \{0, 1\}$ in study wave w. Certain questions (partisanship, out-group animosity, in-group and out-group perception) are only asked if a participant labeled the tweet as being a political tweet. If the participant did not label the tweet as being political, then we let $Y_{w,u,t,q}(x) = 0$ for those questions. This corresponds to assuming that the tweet does not have a partisan leaning, does not contain out-group animosity, and did not impact the readers' perception of their in-group or out-group.

Then, let

$$\bar{Y}_{u,q}(x) = \sum_{w \in \mathcal{W}_u} \frac{1}{|\mathcal{W}_u|} \sum_{t \in \mathcal{T}_{w,u}(x)} \frac{Y_{w,u,t,q}(x)}{|\mathcal{T}_{w,u}(x)|} \tag{1}$$

be the mean outcome on question q across the tweets in timeline x for individual u. The individual effect for participant u on question q is estimated as $\bar{Y}_{u,q}(1) - \bar{Y}_{u,q}(0)$. Finally, the average treatment effect on question q across participants is estimated as

$$\widehat{ATE}(q) = \frac{\sum_u \bar{Y}_{u,q}(1) - \bar{Y}_{u,q}(0)}{|\mathcal{U}|}\,. \tag{2}$$

In addition to considering tweets overall, we also look at the effect on authors' emotions, readers' emotions, and the users' stated preference for political tweets only. Let $\mathcal{T}^{\text{pol}}_{w,u}(x)$ be the tweets that participant u labeled as being political in study wave w and timeline x. Furthermore, let $\mathcal{W}^{\text{pol}}_u$ be the set of waves in which participant u had at least one political tweet in both the chronological and engagement timeline. Then, let

$$\bar{Y}^{\text{pol}}_{u,q}(x) = \sum_{w \in \mathcal{W}^{\text{pol}}_u} \frac{1}{|\mathcal{W}^{\text{pol}}_u|} \sum_{t \in \mathcal{T}^{\text{pol}}_{w,u}(x)} \frac{Y_{w,u,t,q}(x)}{|\mathcal{T}^{\text{pol}}_{w,u}(x)|} \tag{3}$$

be the participant's mean outcome for question q when considering only political tweets. Then, the average treatment effect for political tweets only is

$$\widehat{ATE}_{\text{pol}}(q) = \frac{\sum_{u \in \mathcal{U}_{\text{pol}}} \bar{Y}^{\text{pol}}_{u,q}(1) - \bar{Y}^{\text{pol}}_{u,q}(0)}{|\mathcal{U}_{\text{pol}}|}\,, \tag{4}$$

where $\mathcal{U}_{\text{pol}}$ is the set of participants with at least one political tweet in each timeline.

Significance and multiple testing. To compute p-values, we conducted two-tailed paired permutation tests. A participant's responses to their personalized tweets and responses to their chronological tweets are considered paired data, i.e., $\bar{Y}_{u,q}(1)$ and $\bar{Y}_{u,q}(0)$, or $\bar{Y}^{\text{pol}}_{u,q}(1)$ and $\bar{Y}^{\text{pol}}_{u,q}(0)$ are considered paired data. We used bootstrap sampling to calculate the 95% confidence intervals displayed in Figure 1. To adjust for the possibility of chance findings when conducting multiple tests, we used the Benjamini-Krieger-Yekutieli two-stage method [8], as described by [4], to compute false discovery rate (FDR) adjusted p-values. The FDR-adjusted p-value (sometimes called a *q-value*) for each test is the lowest false discovery rate such that the test would still be rejected at a significance level of 0.05. We test 26 outcomes in total, and all significant results (significant at a p-value threshold of 0.05) remain significant at a false discovery rate of 0.01. Thus, on expectation, none of our discoveries are false discoveries. The full table of standardized and unstandardized effect sizes, p-values, and FDR-adjusted p-values can be found in SM section S3.

Effect size standardization. When displaying the effect sizes for all outcomes in Figure 1, we standardize using the standard deviation of the outcome in the chronological timeline, i.e., $\sigma(\bar{Y}_{u,q}(0))$. Note that we do not use the pooled standard deviation between the chronological and engagement-based timeline as would be done in Cohen's d. The standardized effect size we present is also known as Glass's Δ and uses the standard deviation of the control group in order to allow comparison between multiple treatments, i.e., not just Twitter's engagement-based algorithm but also our alternative ranking algorithm based on stated preferences. One exception is that when calculating the standardized effect sizes for author emotion, reader emotion, and explicit value for *political* tweets only, we standardize the effect using the standard deviation of that outcome across *all* tweets. This was done to ensure that the effect sizes found when restricting to political tweets could be compared with the effect sizes observed for all tweets, i.e., we use $\sigma(\bar{Y}_{u,q}(0))$ instead of $\sigma(\bar{Y}^{\text{pol}}_{u,q}(0))$.

S2 User-level survey questions and demographics

In this section, we report on the demographic distribution of our participants and compare our distribution to that of Twitter users from the 2020 ANES study [3]. The primary differences are that our population is younger (53 percent of our study are aged 18-34 years old, compared to 33 percent in the ANES study) and more likely to affiliate with the Democratic Party (56 percent Democrat in our study versus 43 percent in the ANES study).

S2.1 Demographics of our study

Participants in our study had the opportunity to take part in up to four waves, with demographic information collected at each wave. In total, 806 unique users participated in our study 1730 times across waves. The following tables report demographics across all users. For users that participated in multiple waves, we report on their demographic information from their first wave. In addition to demographic questions, we also asked two user-level survey questions about the primary reason participants use Twitter and the primary type of content they saw in the tweets we showed them (Table S14).

**Race (Our Study)**	**n**	**Percent**
White	537	66.63
Black or African American	89	11.04
Hispanic	81	10.05
Asian or Native Hawaiian/other Pacific Islander	63	7.82
American Indian/Alaska Native or Other	8	0.99
Multiple races, non-Hispanic	28	3.47
**Total**	**806**	**100**

Table S3: We ask two separate questions about race and ethnicity to participants, and for comparison, we combine them together in the same way that the 2020 ANES study does.

**Gender (Our Study)**	**n**	**Percent**
Man	410	50.87
Woman	371	46.03
Non-binary	21	2.61
Other	4	0.50
**Total**	**806**	**100**

Table S4: Distribution of participants' genders.

**Ideological Leaning (Our Study)**	**n**	**Percent**
Far left	170	21.09
Left	322	39.95
Moderate	196	24.32
Right	86	10.67
Far right	25	3.10
Other	7	0.87
**Total**	**806**	**100**

Table S5: Distribution of participants' ideological leanings.

**Political Leaning Further (Our Study)**	**n**	**Percent**
Towards the Left	136	62.67
Towards the Right	81	37.33
**Total**	**217**	**100**

Table S6: Those who identified as being moderate were asked whether they leaned more toward the Left/Right.

**Summary Leaning (Our Study)**	**n**	**Percent**
Left-leaning	618	76.67
Right-leaning	188	23.33
**Total**	**806**	**100**

Table S7: Participants who selected "Moderate" or "Other" in response to the ideological leaning question were asked a follow-up question about whether they lean towards left or right more as of today. The table shows aggregate counts of those who identified as being on the Left/Right or that they leaned more towards the Left/Right.

**Political Party (Our Study)**	**n**	**Percent**
Democrat	451	55.96
Republican	111	13.77
Independent	205	25.43
Something else	39	4.84
**Total**	**806**	**100**

Table S8: Distribution of participants' political party affiliations.

**Political Party Further (Our Study)**	**n**	**Percent**
Republican	72	29.27
Democrat	174	70.73
**Total**	**246**	**100**

Table S9: Participants who selected "Independent" or "Something else" in response to the political party question were asked a follow-up question about whether they lean towards the Democrat or Republican party more as of today.

**Summary Party (Our Study)**	**n**	**Percent**
Republican	182	22.58
Democrat	624	77.42
**Total**	**806**	**100**

Table S10: Participants who selected "Independent" or "Something else" in response to the political party question were asked a follow-up question about whether they lean towards the Democrat or Republican party more as of today. The table shows aggregate counts of those who said they were Democrat/Republican or lean Democrat/Republican.

**Education Levels (Our Study)**	**n**	**Percent**
Some high school	5	0.62
High school graduate	242	30.02
Associate degree	102	12.66
Bachelor's degree	310	38.46
Master's degree or above	128	15.88
Other	17	2.11
Prefer not to answer	2	0.25
**Total**	**806**	**100**

Table S11: Distribution of participants' education levels.

**Age Group (Our Study)**	**n**	**Percent**
18-24 years old	120	14.89
25-34 years old	307	38.09
35-44 years old	211	26.18
45-54 years old	93	11.54
55-64 years old	51	6.33
65-74 years old	23	2.85
75 years or older	1	0.12
**Total**	**806**	**100**

Table S12: Distribution of participants' ages.

Annual Household Income (Our Study)	n	Percent
Less than $25,000	118	14.64
$25,000-$50,000	188	23.33
$50,000-$100,000	300	37.22
$100,000-$200,000	149	18.49
More than $200,000	35	4.34
Prefer not to say	16	1.99
**Total**	**806**	**100**

Table S13: Distribution of participants' income.

Primary Category of Content (Our Study)	n	Percent
Entertainment	416	28.75
Other	108	7.46
Politics	483	33.38
News	272	18.80
Hobbies	152	10.50
Work	16	1.11
**Total**	**1447**	**100**

Table S14: At the end of the study, we ask participants about the content shown to them: "What were the tweets we showed you today predominantly about? Select a maximum of two."

Primary Reason for Using Twitter (Our Study)	n	Percent
A way to stay informed	293	36.35
Entertainment	393	48.76
Keeping me connected to other people	53	6.58
It's useful for my job or school	28	3.47
Lets me see different points of view	18	2.23
A way to express my opinions	21	2.61
**Total**	**806**	**100**

Table S15: We ask participants about the primary reason they use Twitter: "What would you say is the main reason you use Twitter?"

S2.2 Demographics of Twitter users in ANES 2020 study

In this subsection, we present demographic distributions for the 1030 participants in the 2020 ANES study [3] who reported using Twitter at least a few times a week (only those who reported using Twitter at least a few times a week were allowed to participate in our study). For certain demographic questions, participants were not given the option to opt out in our study, but they were in the ANES study. Thus, for those cases, we do not include the ANES participants who opted out of the question.

Comparing the demographics of users in the ANES 2020 study to those of users in our study, the largest differences are that our population is younger (53 percent of our study are aged 18-34 years old, compared to 33 percent in the ANES study) and more likely to affiliate with the Democratic Party (56 percent Democrat in our study versus 43 percent in the ANES study). That said, all distributions over

demographic attributes are significantly different (when using a chi-squared test), apart from sex/gender.

**Race/Ethnicity**	**ANES**		**Our Study**	
	n	Percent	n	Percent
White	729	71.19	537	66.63
Black or African American	71	6.93	89	11.04
Hispanic	113	11.04	81	10.05
Asian or Native Hawaiian/other Pacific Islander	49	4.79	63	7.82
American Indian/Alaska Native or Other	25	2.44	8	0.99
Multiple races, non-Hispanic	37	3.61	28	3.47
**Total**	**1024**	**100.00**	**806**	**100.00**

Table S16: Distribution of race and ethnicity comparing 2020 ANES Twitter population with our study population ($\chi^2(5) = 22.53$, $p < .001$).

**Sex/Gender**	**ANES**		**Our Study**	
	n	Percent	n	Percent
Male/Man	558	54.39	410	52.50
Female/Woman	468	45.61	371	47.50
**Total**	**1026**	**100.00**	**781**	**100.00**

Table S17: Distribution of sex/gender comparing 2020 ANES Twitter population with our study population ($\chi^2(1) = 0.56$, $p = .453$).

**Political Party**	**ANES**		**Our Study**	
	n	Percent	n	Percent
Democrat	439	42.75	451	55.96
Republican	222	21.62	111	13.77
Independent	338	32.91	205	25.43
Other	28	2.73	39	4.84
**Total**	**1027**	**100.00**	**806**	**100.00**

Table S18: Distribution of political party affiliation comparing 2020 ANES Twitter population with our study population ($\chi^2(3) = 45.56$, $p < .001$).

Education Level	ANES		Our Study	
	n	Percent	n	Percent
Some high school	20	1.94	5	0.62
High school graduate	285	27.67	242	30.02
Associate degree	97	9.42	102	12.66
Bachelor's degree	341	33.11	310	38.46
Master's degree or above	275	26.70	128	15.88
Other	12	1.17	17	2.11
Prefer not to answer	0	0.00	2	0.25
**Total**	**1030**	**100.00**	**806**	**100.00**

Table S19: Distribution of educational attainment in the 2020 ANES Twitter population compared to our study population ($\chi^2(6) = 43.92$, $p < .001$). For comparison purposes, we bin the ANES education categories, which are more fine-grained than ours, into our coarser categories.

Age Group	ANES		Our Study	
	n	Percent	n	Percent
18-24 years old	131	13.14	120	14.89
25-34 years old	201	20.16	307	38.09
35-44 years old	235	23.57	211	26.18
45-54 years old	164	16.45	93	11.54
55-64 years old	137	13.74	51	6.33
65-74 years old	98	9.83	23	2.85
75 years or older	31	3.11	1	0.12
**Total**	**997**	**100.00**	**806**	**100.00**

Table S20: Distribution of ages in the 2020 ANES Twitter population compared to our study population ($\chi^2(6) = 138.78$, $p < .001$). For comparison purposes, we group ages to match the age groups that we use in our study.

S3 Pre-registered analysis

Outcome	Standardized Effect	Unstandardized Effect	Chron. Mean	Eng. Mean	p-value	Adjusted p-value
**Emotional effects (all tweets)**						
Author Angry	0.473	0.188	0.352	0.545	0.0002	0.0005
Author Sad	0.220	0.077	0.293	0.378	0.0002	0.0005
Author Anxious	0.232	0.100	0.391	0.492	0.0002	0.0005
Author Happy	0.016	0.013	1.307	1.330	0.5125	0.1340
Reader Angry	0.266	0.107	0.306	0.412	0.0002	0.0005
Reader Sad	0.086	0.032	0.316	0.356	0.0032	0.0025
Reader Anxious	0.119	0.051	0.346	0.398	0.0002	0.0005
Reader Happy	0.119	0.085	0.941	1.030	0.0002	0.0005
**Emotional effects (political tweets only)**						
Author Angry	0.754	0.299	1.128	1.438	0.0002	0.0005
Author Sad	0.309	0.108	0.688	0.782	0.0056	0.0035
Author Anxious	0.175	0.075	0.824	0.873	0.0316	0.0129
Author Happy	-0.061	-0.048	0.513	0.532	0.2138	0.0620
Reader Angry	0.377	0.152	1.042	1.192	0.0008	0.0009
Reader Sad	0.007	0.003	0.835	0.840	0.9363	0.2288
Reader Anxious	-0.002	-0.001	0.867	0.863	0.9679	0.2288
Reader Happy	-0.015	-0.011	0.421	0.471	0.7761	0.1929
**Political effects**						
Partisanship	0.244	0.051	0.151	0.202	0.0002	0.0005
Out-group Animosity	0.236	0.031	0.085	0.116	0.0002	0.0005
In-group Perc. (all users)	0.081	0.015	0.060	0.074	0.0014	0.0014
Out-group Perc. (all users)	-0.171	-0.037	-0.108	-0.147	0.0002	0.0005
In-group Perc. (left users)	0.052	0.009	0.056	0.066	0.0954	0.0329
Out-group Perc. (left users)	-0.157	-0.031	-0.097	-0.132	0.0002	0.0005
In-group Perc. (right users)	0.151	0.031	0.072	0.102	0.0012	0.0013
Out-group Perc. (right users)	-0.204	-0.052	-0.145	-0.200	0.0002	0.0005
**Reader Preference**						
Reader Pref (all tweets)	0.065	0.023	0.507	0.526	0.0226	0.0097
Reader Pref (political tweets)	-0.180	-0.065	0.519	0.465	0.0054	0.0035

Table S21: Our pre-registered analysis measuring the effects of Twitter's engagement-based timeline. The table shows the average treatment effects (standardized and unstandardized), p-values, and FDR-adjusted p-values for all 26 pre-registered outcomes. The way that all statistics are calculated is described in SM section S1.3.

S4 Exploratory analysis

Next, we describe additional, exploratory analyses that were not pre-registered.

S4.1 Descriptive statistics: tweet metadata

Table S22 compares the metadata of the top 10 tweets in the engagement-based and chronological timelines, pooled across all participants and waves. We include both the mean and median for each property. The median may be a more appropriate summary for attributes with extreme outliers, i.e., the tweet age (the amount of time since the tweet was created), the author's number of followers, the number of likes, and the number of retweets. Histograms for these properties are provided in Figure S2.

Unsurprisingly, the number of likes and retweets that each tweet has is much higher in the engagement-based timeline. And as expected, the reader is less likely to follow the authors of tweets shown by the engagement-based algorithm compared to the chronological ranking. Interestingly, the average number of links is almost halved, which is consistent with the results of a prior study that used eight sock puppets to audit properties of Twitter's algorithm [6].

The algorithm does not necessarily favor the most "popular" accounts: authors shown by the algorithm have a lower median number of followers and are less likely to be verified. It is important to clarify that our research was conducted before Twitter's changed its verification policy on April 1, 2023. The new policy limits verification (indicated by a blue checkmark on Twitter profiles) to Twitter Blue subscribers who pay for the service. Our data was collected under the previous policy which required users to be "active", "notable", and "authentic" to get verified status. Consequently, the verified users in our data set tend to be official organizational accounts, celebrities, journalists, or other well-known individuals.

Finally, Figure S3 shows the distribution of the number of political tweets in the user timelines. There was little difference between the distribution of political tweets in the engagement-based timelines and the chronological timelines. About 70% of participants' timelines contained at least one political tweet (out of approximately ten tweets total).

S4.2 Amplification of individual accounts

In this section, we investigate which individual user accounts are the most amplified or de-amplified by Twitter's engagement-based algorithm.

First, in Table S23, we show the accounts that appear most frequently in our study participants' chronological and engagement timelines. The most common accounts are quite different in the two timelines, with news outlets having much greater dominance in the chronological timeline.

Next, in Table S24, we show which accounts were most and least amplified by Twitter's algorithm, where the amplification of an individual account is simply calculated as the number of tweets by that author in users' engagement-based timelines minus the number of tweets by that author in users' chronological timelines. Consistent with prior findings [6], the accounts that were most *de-amplified* were news outlets. This is likely because news outlets post more frequently than ordinary accounts. Though the chronological timeline is susceptible to this kind of spam, the engagement-based timeline is not [46]. Thus, whether users are using their chronlogical or engagement-based timeline may have a large influence on their exposure to traditional news outlets [61].

Elon Musk also stands out as a significant outlier, receiving higher amplification than any other account by a wide margin. Notably, during our study period, Platformer alleged that Twitter deployed code to artificially boost the reach of Musk's tweets [53]. Though Musk denied such claims [25], our data seems to suggest that he did receive an abnormally high amount of amplification, relative to other accounts.

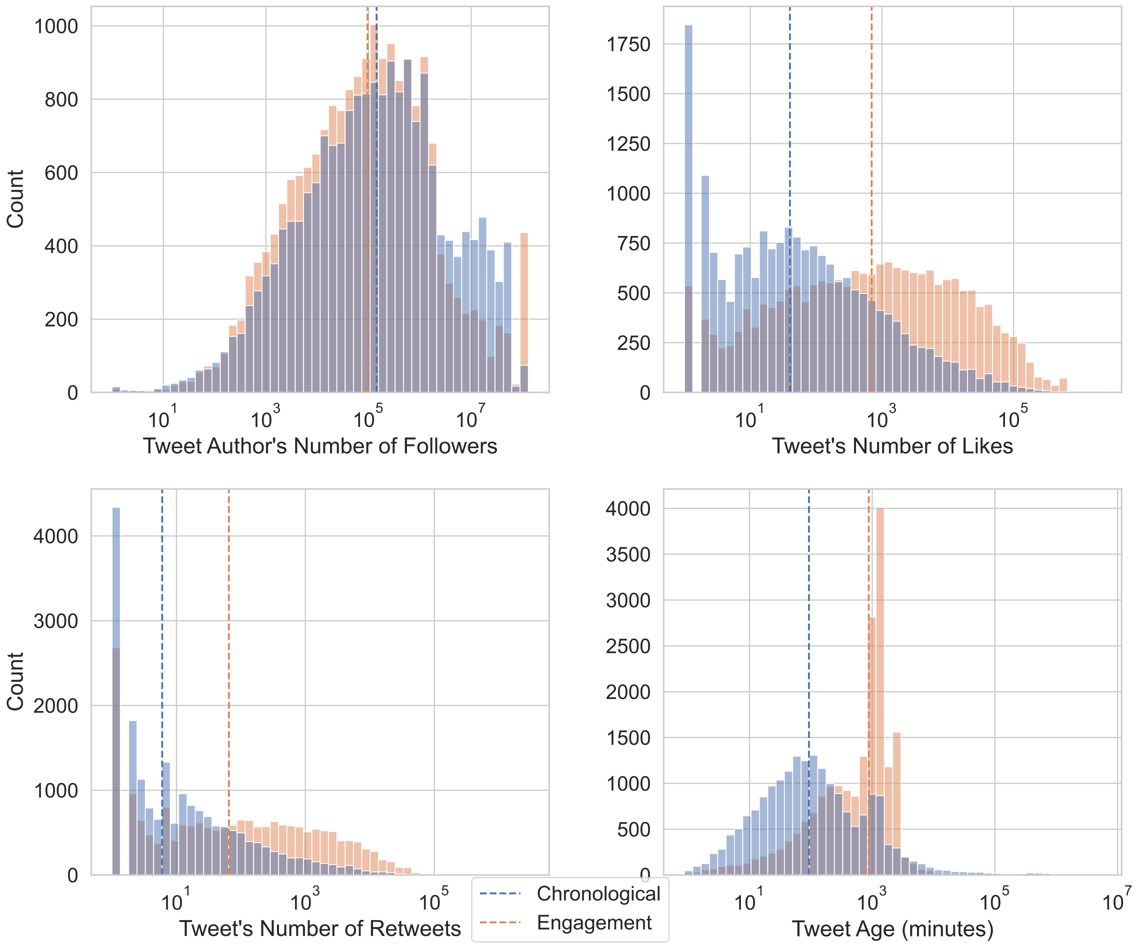

Figure S2: The graphs compare the histogram of tweets in the engagement-based and chronological timelines along four properties: the author's number of followers, the tweet's number of likes, the tweet's number of retweets, and the tweet's age. All graphs are plotted on a log-scale x-axis because all properties have a long-tail of extreme outliers. The dashed lines show the median value for each timeline.

Table S22: Summary of metadata in users' engagement and chronological timelines

	Timeline	p5	p50	p95	Avg.	SD	Diff
Tweet age (minutes)	Chronological	3.8	92	2,937	7,812	126,667	
	Engagement	18.4	899	2,722	3,846	94,308	$p < 0.001$
Number of likes	Chronological	0	39	7,557	2,500	18,565	
	Engagement	2	744	83,658	16,912	62,158	$p < 0.001$
Number of retweets	Chronological	0	6	1,081	389	3,752	
	Engagement	0	68	8,875	1,787	13,415	$p < 0.001$
Number of links in tweet	Chronological	0	0	1	0.397	0.552	
	Engagement	0	0	1	0.105	0.326	$p < 0.001$
Number of photos in tweet	Chronological	0	0	2	0.457	0.755	
	Engagement	0	0	2	0.496	0.752	$p < 0.001$
Number of videos in tweet	Chronological	0	0	1	0.130	0.336	
	Engagement	0	0	1	0.184	0.387	$p < 0.001$
Author's # followers	Chronological	500	139,874	25,745,780	3,905,313	12,059,120	
	Engagement	487	92,959	18,788,850	4,877,343	20,868,580	$p < 0.001$
Is the author verified?	Chronological	0	1	1	0.504	0.500	
	Engagement	0	0	1	0.391	0.488	$p < 0.001$
Does the reader follow the author?	Chronological	0	1	1	0.694	0.461	
	Engagement	0	1	1	0.569	0.495	$p < 0.001$

The table compares the top ten tweets in the chronological and engagement-based timelines. Both the mean and median are shown for each attribute. The median is a more representative summary when the mean of the attribute is dominated by extreme outliers. In gray, we have highlighted which summary statistic, the mean or median, is more representative based on how long-tailed the distribution is.

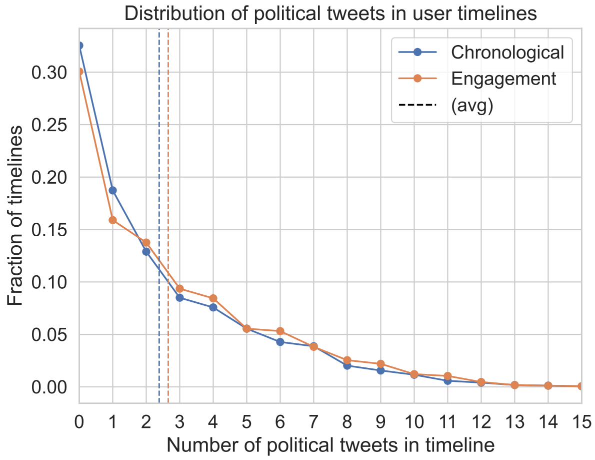

Figure S3: The distribution of political tweets in user timelines. The number of political tweets in each timeline is calculated using the participant's response to the binary question, "Is [@author-handle]'s tweet about a political or social issue?". Notably, about 30% of participants' timelines contain no political tweets.

Chronological		Engagement	
**Top Accounts**	**Count**	**Top Accounts**	**Count**
@Reuters	532	@elonmusk	1387
@nytimes	529	@POTUS	295
@FatKidDeals	407	@RonFilipkowski	210
@Wario64	365	@JackPosobiec	171
@CNN	365	@fasc1nate	152
@NBA	280	@nytimes	135
@Forbes	280	@barstoolsports	134
@FoxNews	266	@DailyLoud	133
@AP	251	@hodgetwins	126
@washingtonpost	250	@CNN	119
@POTUS	206	@NBA	115
@IGN	204	@stillgray	107
@videogamedeals	185	@vidsthatgohard	103
@TMZ	179	@ClownWorld_	102
@elonmusk	151	@catturd2	97
@business	151	@BornAKang	96
@TheOnion	148	@krassenstein	95
@barstoolsports	128	@Wario64	93
@PlayStation	127	@HumansNoContext	90
@NFL	126	@Dexerto	84
@JackPosobiec	123	@CalltoActivism	84
@guardian	122	@mmpadellan	82
@Independent	119	@NFL	82
@ABC	114	@joncoopertweets	81
@nypost	108	@jordanbpeterson	79

Table S23: The most common accounts seen in our study participants' chronological and engagement timelines. In both timelines, only the first ten tweets are considered.

Most Amplified	Eng-Chron Diff	Least Amplified	Eng-Chron Diff
@elonmusk	1236.0	@Reuters	-518.0
@RonFilipkowski	144.0	@nytimes	-394.0
@fasc1nate	119.0	@Wario64	-272.0
@DailyLoud	114.0	@Forbes	-265.0
@hodgetwins	102.0	@CNN	-246.0
@vidsthatgohard	97.0	@AP	-214.0
@stillgray	89.0	@FoxNews	-198.0
@POTUS	89.0	@washingtonpost	-188.0
@BornAKang	87.0	@NBA	-165.0
@ClownWorld_	83.0	@videogamedeals	-156.0
@krassenstein	83.0	@IGN	-148.0
@HumansNoContext	80.0	@TMZ	-148.0
@Dexerto	73.0	@business	-139.0
@rawsalerts	68.0	@TheOnion	-136.0
@joncoopertweets	67.0	@guardian	-117.0
@buitengebieden	60.0	@Independent	-108.0
@Acyn	59.0	@PlayStation	-107.0
@mmpadellan	58.0	@TheEconomist	-85.0
@EndWokeness	58.0	@people	-82.0
@CollinRugg	58.0	@ABC	-79.0
@historyinmemes	57.0	@WSJ	-79.0
@CalltoActivism	53.0	@BBCWorld	-69.0
@libsoftiktok	52.0	@netflix	-68.0
@FightHaven	49.0	@HuffPost	-65.0
@JackPosobiec	48.0	@thehill	-63.0

Table S24: The accounts that were most and least amplified in the engagement timeline, relative to the chronological timeline. In both timelines, only the first 10 tweets are considered.

S4.3 Distribution of responses to survey questions

In SM section S3, we reported our pre-registered results on the effects of the engagement-based timeline, relative to the chronological timeline. Here, we also additionally show how the full distribution of responses to the Likert survey questions differs between users' chronological and engagement-based timelines.

S4.3.1 Emotions

First, we show the distribution of survey responses for questions gauging the author and reader's emotions (sad, happy, anxious, and angry), considering both the distribution for tweets overall and for only political tweets, in Figure S4 and Tables S25-S28. For further ease of interpretation, in Figure S5, we also show the mean level of author and reader emotions in the chronological and engagement-based timelines after binarizing responses. In particular, if a reader responds "Not at all" to an emotion, that is coded as zero, while any response between "Slightly" and "Extremely" is coded as one. Notably, there was a large increase in tweets expressing anger between the engagement and chronological timeline. In particular, 62 percent of political tweets in the engagement timeline expressed anger, compared to 52 percent in the chronological timeline.

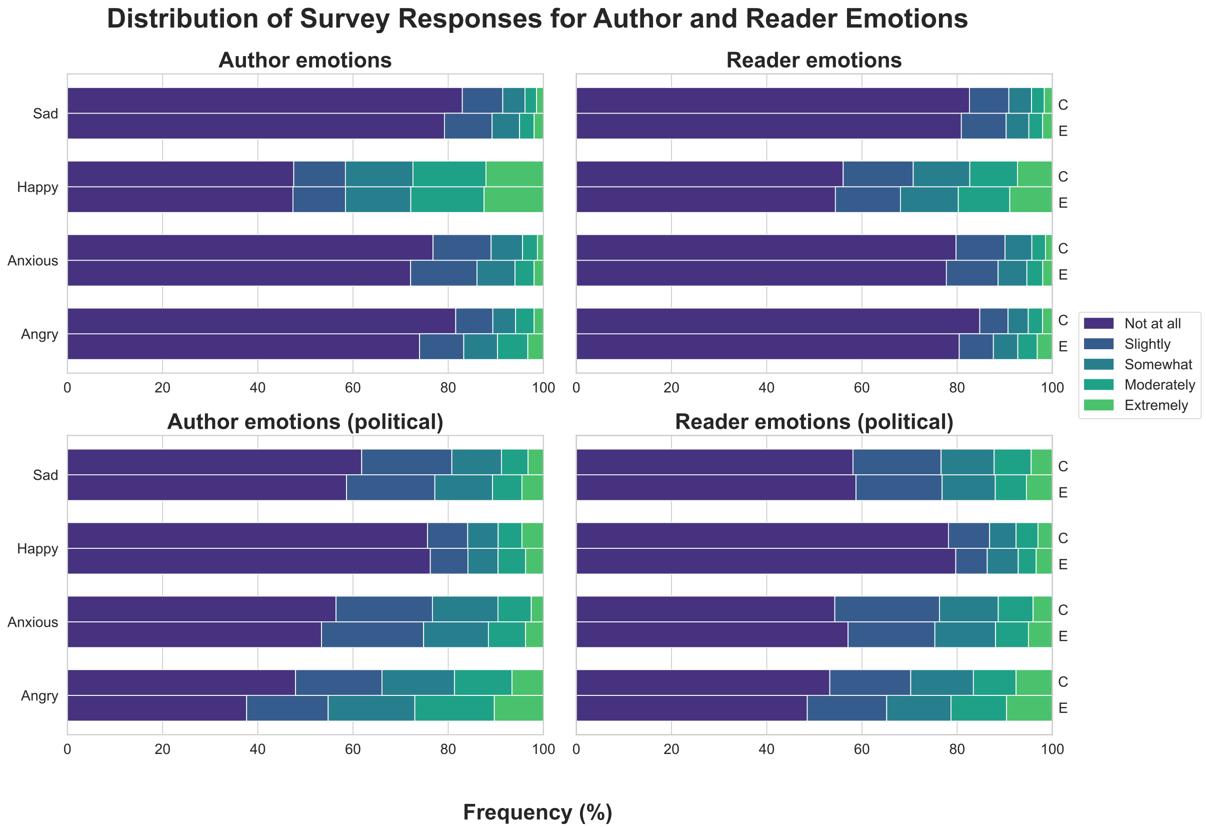

Figure S4: Distribution of author and reader emotions in the chronological (C) and engagement-based (E) timelines

Answer	Angry		Anxious		Happy		Sad	
	**% Chron**	**% Eng**	**% Chron**	**% Eng**	**% Chron**	**% Eng**	**% Chron**	**% Eng**
Not at all (0)	81.57	73.99	76.81	72.10	47.55	47.38	82.96	79.21
Slightly (1)	7.80	9.28	12.20	13.94	10.85	11.04	8.50	10.01
Somewhat (2)	4.79	7.09	6.61	7.99	14.18	13.72	4.67	5.78
Moderately (3)	3.89	6.36	3.12	4.01	15.33	15.40	2.43	3.06
Extremely (4)	1.95	3.28	1.26	1.96	12.08	12.47	1.44	1.95

Table S25: Author Emotions (Overall)

Answer	Angry		Anxious		Happy		Sad	
	**% Chron**	**% Eng**	**% Chron**	**% Eng**	**% Chron**	**% Eng**	**% Chron**	**% Eng**
Not at all (0)	84.75	80.44	79.76	77.73	56.06	54.42	82.58	80.88
Slightly (1)	5.94	7.17	10.30	10.87	14.71	13.66	8.28	9.42
Somewhat (2)	4.25	5.13	5.63	6.04	11.88	12.16	4.77	4.77
Moderately (3)	3.01	4.09	2.86	3.34	10.04	10.81	2.67	2.89
Extremely (4)	2.05	3.17	1.45	2.02	7.31	8.95	1.70	2.04

Table S26: Reader Emotions (Overall)

Answer	Angry		Anxious		Happy		Sad	
	**% Chron**	**% Eng**	**% Chron**	**% Eng**	**% Chron**	**% Eng**	**% Chron**	**% Eng**
Not at all (0)	47.93	37.65	56.43	53.38	75.66	76.24	61.82	58.64
Slightly (1)	18.15	17.13	20.26	21.45	8.44	7.90	18.94	18.55
Somewhat (2)	15.23	18.21	13.76	13.61	6.40	6.33	10.43	12.12
Moderately (3)	12.09	16.70	7.00	7.80	4.99	5.79	5.59	6.17
Extremely (4)	6.60	10.31	2.55	3.76	4.52	3.74	3.23	4.52

Table S27: Author Emotions (Political)

Answer	Angry		Anxious		Happy		Sad	
	**% Chron**	**% Eng**	**% Chron**	**% Eng**	**% Chron**	**% Eng**	**% Chron**	**% Eng**
Not at all (0)	53.24	48.51	54.30	57.09	78.17	79.70	58.10	58.75
Slightly (1)	17.00	16.68	21.99	18.22	8.65	6.61	18.52	18.09
Somewhat (2)	13.16	13.51	12.35	12.76	5.55	6.51	11.15	11.17
Moderately (3)	8.96	11.68	7.32	6.91	4.61	3.77	7.77	6.54
Extremely (4)	7.63	9.62	4.04	5.03	3.02	3.40	4.45	5.45

Table S28: Reader Emotions (Political)

Timeline	Author				Reader			
	**Angry**	**Anxious**	**Happy**	**Sad**	**Angry**	**Anxious**	**Happy**	**Sad**
Chronological	18.396	23.186	52.448	17.020	15.243	20.225	43.973	17.414
Engagement	25.963	27.897	52.687	20.786	19.540	22.272	45.642	19.119

Table S29: Binarized Emotions (Overall). The mean percent of each binarized emotion for readers and authors across each timeline.

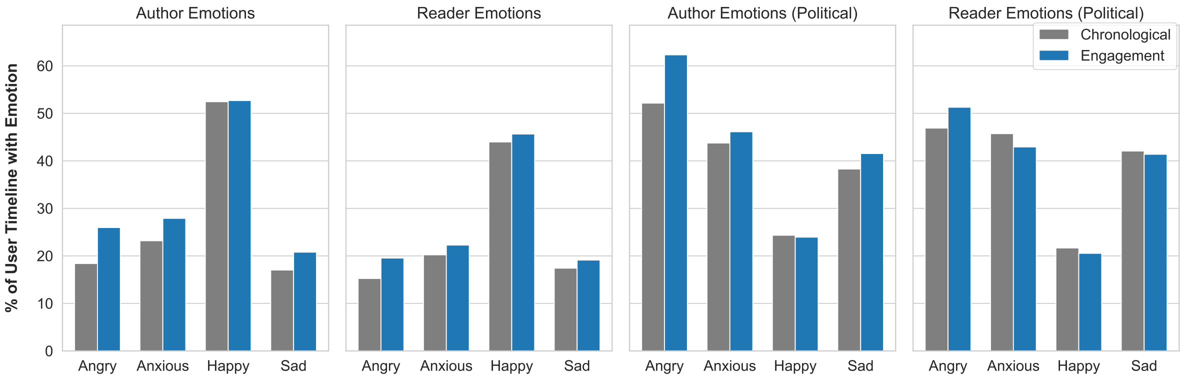

Figure S5: The percentage of each (binarized) emotion in the engagement and chronological timelines.

**Timeline**	**Author**				**Reader**			
	**Angry**	**Anxious**	**Happy**	**Sad**	**Angry**	**Anxious**	**Happy**	**Sad**
Chronological	52.151	43.752	24.341	38.281	46.893	45.729	21.663	42.065
Engagement	62.312	46.123	23.944	41.538	51.279	42.929	20.536	41.383

Table S30: Binarized Emotions (Political). The mean percent of each binarized emotion for readers and authors across each timeline, limited to just political tweets.

S4.3.2 Political outcomes

Next, we show the distribution of survey responses for political questions (political leaning, out-group animosity, in-group perception, and out-group perception). We show the distributions for users overall (Figure S6, Tables S31-S33) as well as separately for left-leaning (Figure S7, Tables S34-S36) and right-leaning users (Figure S8, Tables S37-S39).

Interestingly, in the chronological timeline, right-leaning users are exposed to much more cross-cutting content than left-leaning users; 21 percent of the political tweets shown to right-leaning users are left-leaning while only 8 percent of the political tweets shown to left-leaning users are right-leaning. This may reflect the fact the population of Twitter users tends to be predominantly left-leaning (see SM section S2). The engagement-based algorithm doubles the amount of cross-cutting content that left-leaning users see (16 percent compared to 8 percent in the chronological timeline). For right-leaning users, the proportion of cross-cutting content remains the same, i.e., 21 percent in both timelines.

**Answer**	**% Chronological**	**% Engagement**
Far left (-2)	8.48	10.64
Left (-1)	34.20	32.70
Moderate (0)	41.27	33.94
Right (1)	12.20	15.28
Far right (2)	3.85	7.44

Table S31: Political leaning (all users)

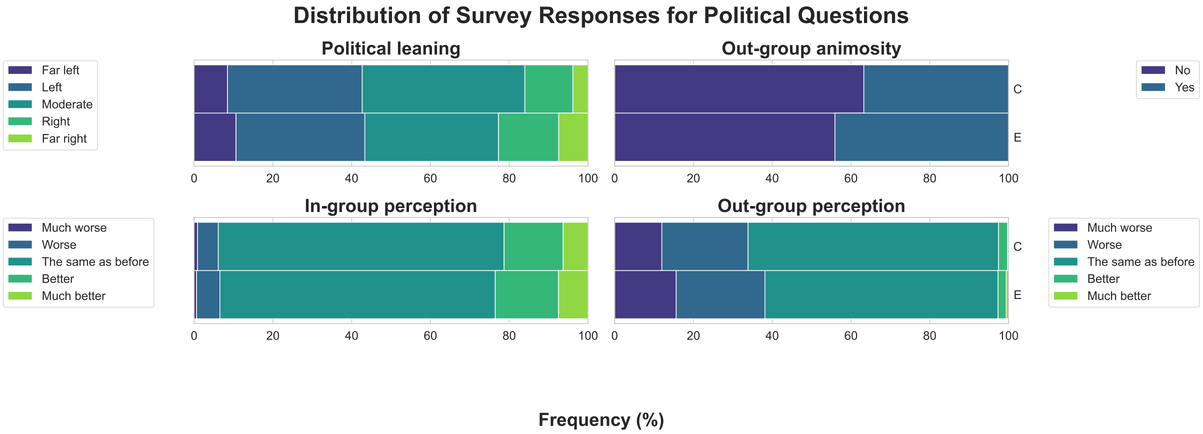

Figure S6: Distribution of political outcomes outcomes in the chronological (C) and engagement-based (E) timelines for all users

**Answer**	**In-group perc.**		**Out-group perc.**	
	**% Chron**	**% Eng**	**% Chron**	**% Eng**
Much worse (-2)	0.87	0.64	11.97	15.63
Worse (-1)	5.27	5.91	21.90	22.51
The same as before (0)	72.56	69.91	63.58	59.21
Better (1)	15.00	16.07	2.33	2.07
Much better (2)	6.30	7.47	0.22	0.58

Table S32: In-group and out-group perception (all users)

**Answer**	**% Chronological**	**% Engagement**
No (0)	63.26	55.92
Yes (1)	36.74	44.08

Table S33: Out-group animosity (all users)

**Answer**	**% Chronological**	**% Engagement**
Far left (-2)	9.77	11.42
Left (-1)	39.09	39.05
Moderate (0)	42.76	33.85
Right (1)	6.12	9.54
Far right (2)	2.26	6.15

Table S34: Political leaning for left-leaning users only

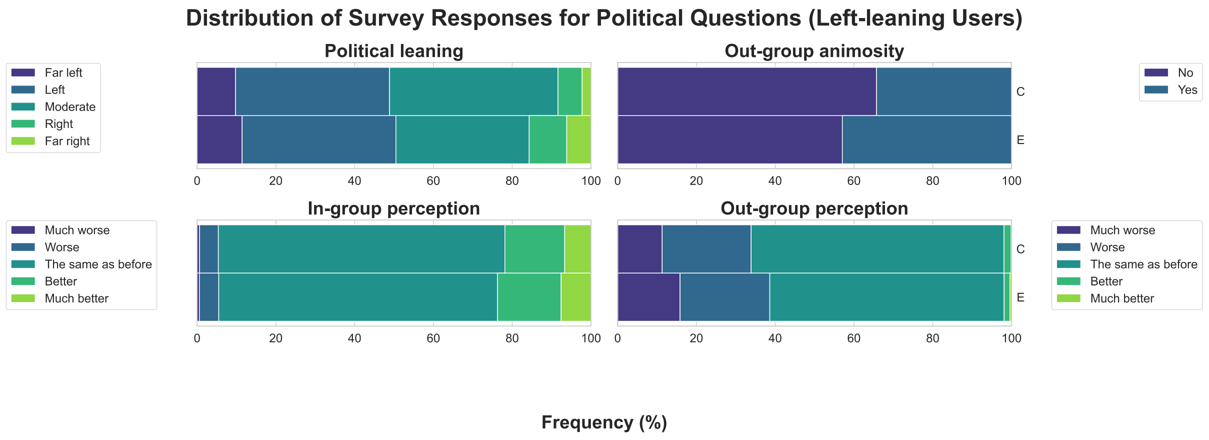

Figure S7: Distribution of political outcomes in the chronological (C) and engagement-based (E) timelines for left-leaning users only

**Answer**	**In-group perc. (left users)**		**Out-group perc. (left users)**	
	**% Chron**	**% Eng**	**% Chron**	**% Eng**
Much worse (-2)	0.64	0.58	11.30	15.84
Worse (-1)	4.74	4.91	22.59	22.78
The same as before (0)	72.76	70.76	64.19	59.49
Better (1)	15.19	16.16	1.82	1.48
Much better (2)	6.68	7.59	0.10	0.41

Table S35: In-group and out-group perception for left-leaning users only

**Answer**	**% Chronological**	**% Engagement**
No (0)	65.72	57.04
Yes (1)	34.28	42.96

Table S36: Out-group animosity for left-leaning users only

**Answer**	**% Chronological**	**% Engagement**
Far left (-2)	4.11	8.19
Left (-1)	17.37	12.65
Moderate (0)	36.96	34.74
Right (1)	32.46	33.17
Far right (2)	9.10	11.24

Table S37: Political leaning for right-leaning users only

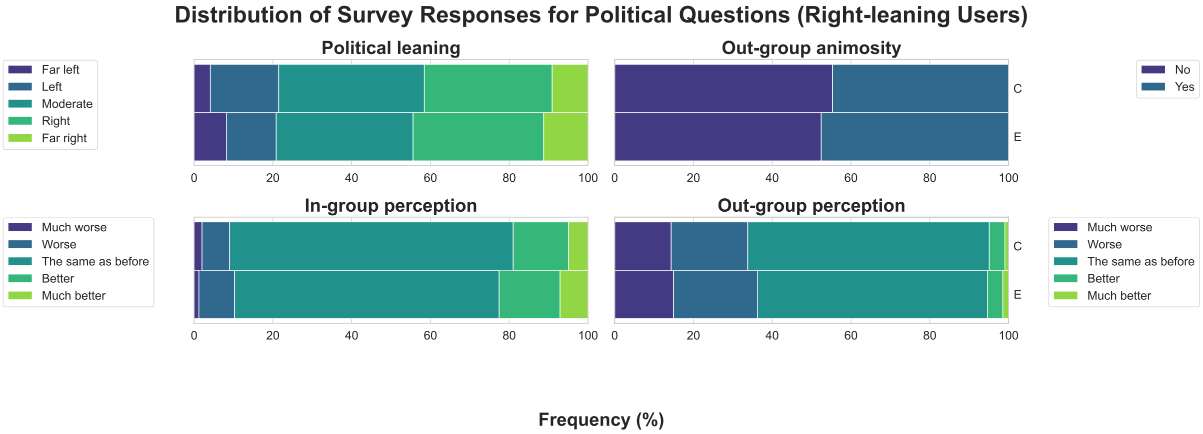

Figure S8: Distribution of political outcomes in the chronological (C) and engagement-based (E) timelines for right-leaning users only

**Answer**	**In-group**		**Out-group**	
	**% Chron**	**% Eng**	**% Chron**	**% Eng**
Much worse (-2)	2.01	1.21	14.35	14.94
Worse (-1)	7.02	9.06	19.44	21.29
The same as before (0)	71.97	67.17	61.32	58.40
Better (1)	14.07	15.47	3.97	3.91
Much better (2)	4.92	7.09	0.93	1.46

Table S38: In-group and out-group perception for right-leaning users only

**Answer**	**% Chronological**	**% Engagement**
No (0)	55.33	52.44
Yes (1)	44.67	47.56

Table S39: Out-group animosity for right-leaning users only

S4.3.3 Reader's stated preference

Next, we show how the distribution of users' stated preference for tweets in their chronological and engagement-based timelines differs. Here we examine stated preference across all tweets and just political tweets. Notably, when considering all tweets, readers see similar percentages of unwanted tweets in both timelines (13 percent for both the chronological and engagement timelines). However, when we restrict to only political tweets, 22 percent of tweets in the engagement timeline are unwanted while only 16 percent of tweets in the chronological timeline are unwanted.

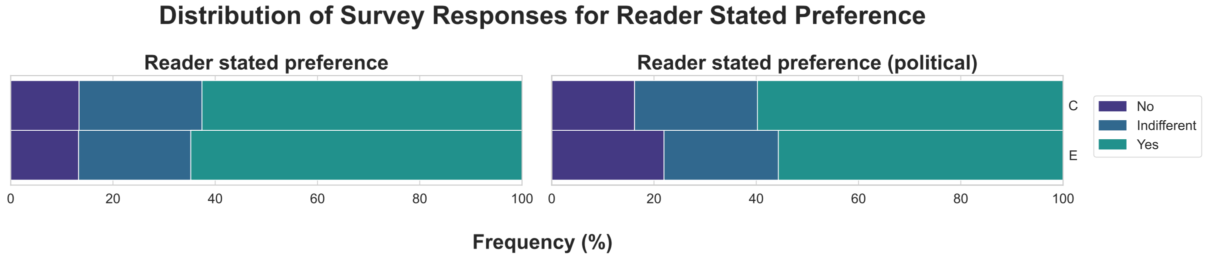

Figure S9: Distribution of readers' stated preference in the chronological (C) and engagement-based (E) timelines across all tweets (left figure) and political tweets only (right figure)

Answer	% Chronological	% Engagement
No (-1)	13.38	13.29
Indifferent (0)	24.03	21.94
Yes (1)	62.59	64.78

Table S40: Reader stated preference

Answer	% Chronological	% Engagement
No (-1)	16.21	21.96
Indifferent (0)	24.02	22.37
Yes (1)	59.77	55.68

Table S41: Reader stated preference after restricting to political tweets only

S4.4 Effects of Engagement Timeline with Varied Tweet Threshold

We only survey users about the first 10 tweets in their engagement-based timeline and in their chronological timeline. Thus, when computing the average treatment effect (ATE) of Twitter's engagement-based timeline, we only consider the first 10 tweets in both. To evaluate the robustness of our results to this choice of threshold, here we also calculate the ATE across a range of thresholds, spanning from 5 to 10 tweets. Across these thresholds, our findings were consistently robust. Nonetheless, potential differences could emerge for tweets beyond the top ten. As future work, it would be interesting to execute a broader robustness analysis that encompasses thresholds beyond the initial ten tweets.

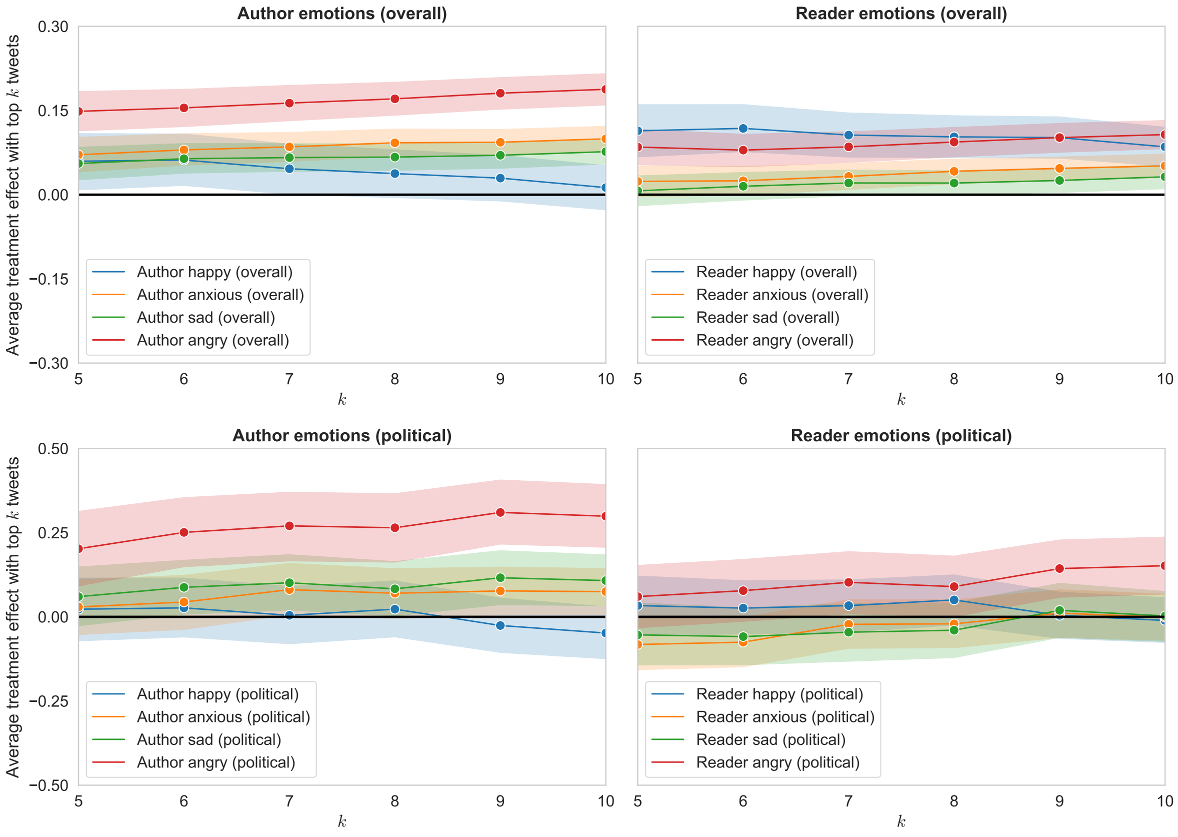

Figure S10: The ATE, based on different tweet thresholds, for the author and readers' emotions. The top two plots show these outcomes for all tweets, and the bottom plots restrict to only political tweets. The effect sizes are in the original units and are not standardized.

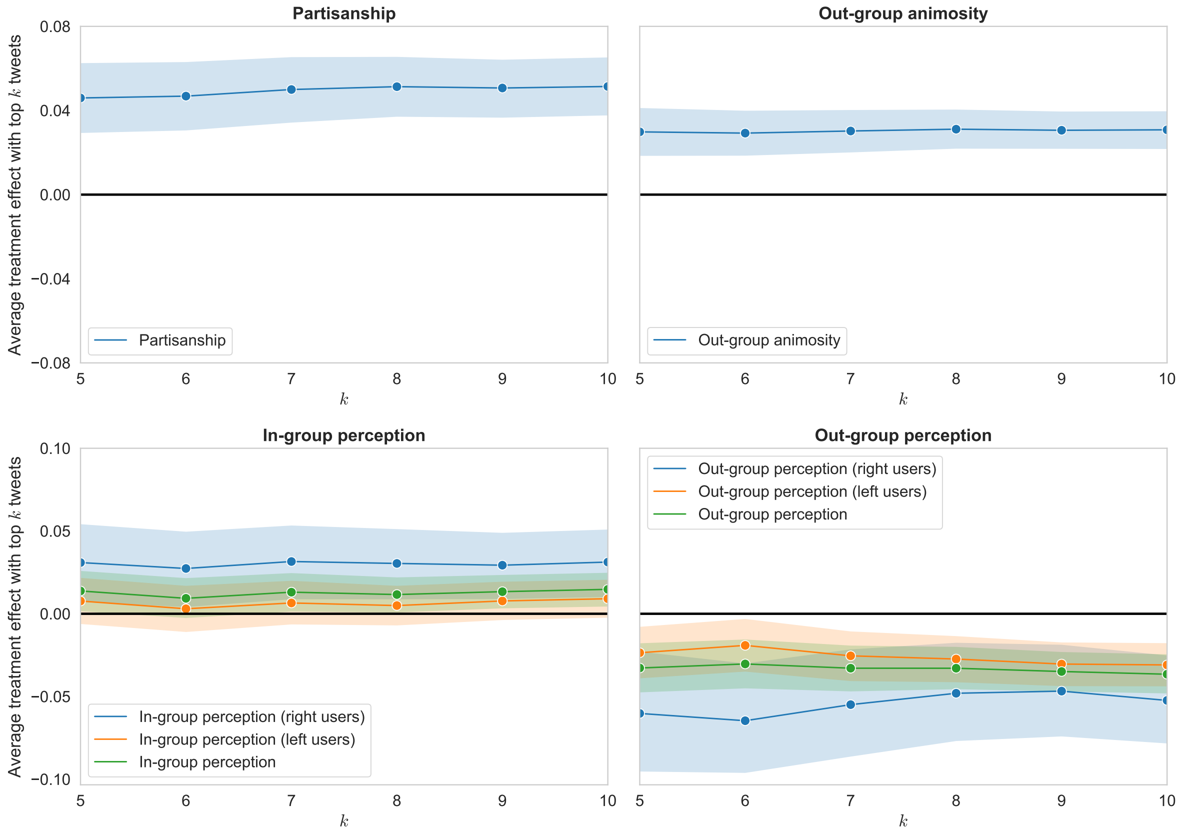

Figure S11: The ATE, based on different tweet thresholds, for partisanship, out-group animosity, in-group perception, and out-group perception. The effect sizes are in the original units and are not standardized.

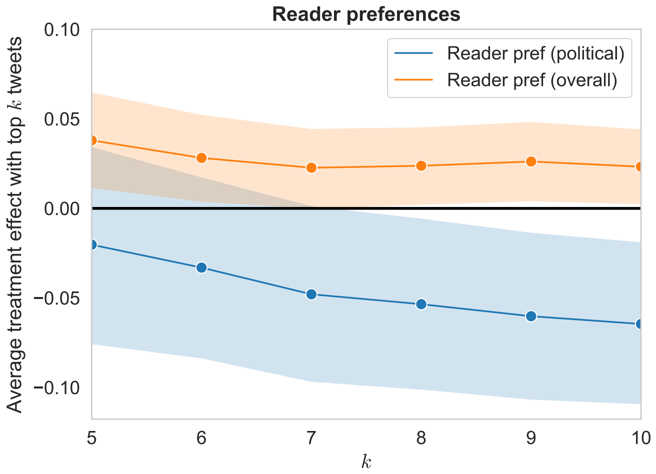

Figure S12: The ATE, based on different tweet thresholds, for the readers' explicit preference for the tweet. The effect sizes are in the original units and are not standardized.

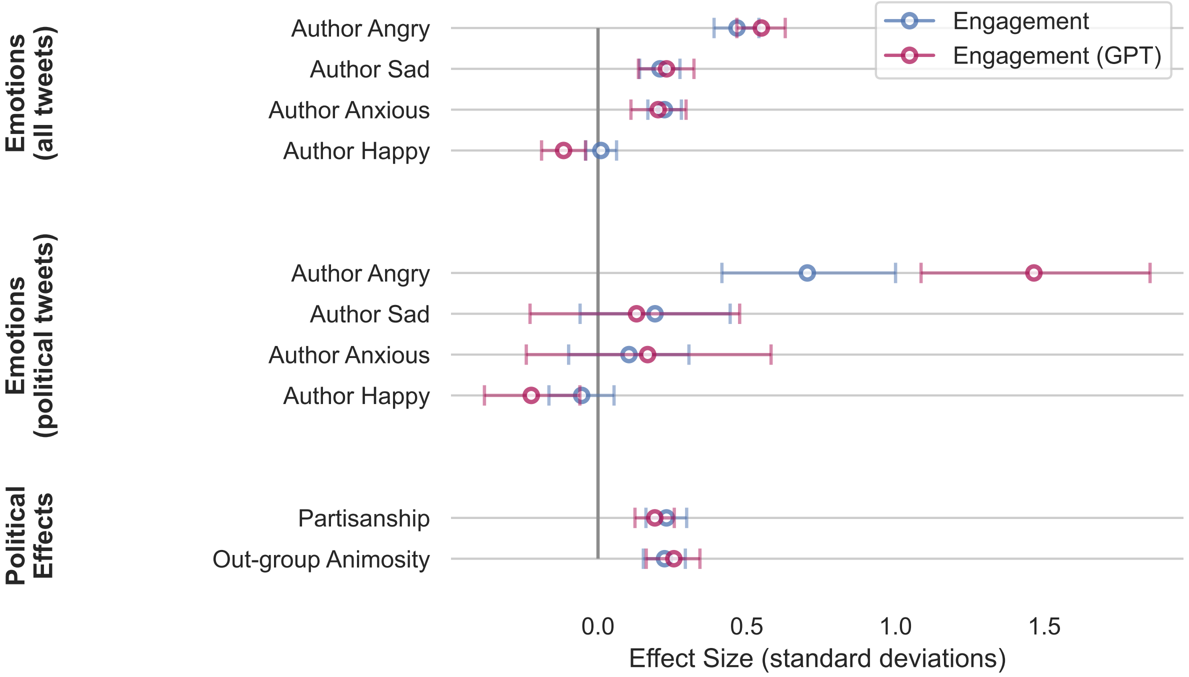

Figure S13: A comparison of the average treatment effects for tweet-based outcomes, using both the labels given by our participants and labels given by GPT-4. For this comparison, only tweets that GPT-4 provided a validly formatted response to were used to estimate the average treatment effects (for both the ATEs calculated with the human labels and the ATEs calculated with the GPT-4 labels).

S4.5 Effects of engagement timeline with GPT-4 labels

In our main analysis, all outcomes are measured through survey questions given to readers; this includes both outcomes that explicitly ask about the reader's perspective (i.e., the reader's emotions, in-group perceptions, out-group perceptions, and stated preferences) as well as outcomes that pertain to the tweet itself (the author's emotions, the ideological leaning of the tweet, and whether the tweet contains out-group animosity). Here, we elicit labels for the tweet-based outcomes from GPT-4 [45] and report effects based on these machine learning labels (Table S42 and Figure S13).

All the results based on GPT-4 judgments are notably similar to the results based on reader judgments. Even under the GPT-4 labels, the algorithm is found to amplify content that is more emotional (especially angry), partisan, and likely to contain out-group animosity. The main difference in results is that GPT-4 judges the political tweets chosen by the engagement-based algorithm to be even angrier than how humans judged the tweets to be.

S4.5.1 Prompts and method

We had GPT-4 (`gpt-4-1106-preview` in the OpenAI API) label the 28,301 unique tweets shown to users in our study. We asked GPT-4 to label the author's emotions in the tweet (along the four dimensions of angry, sad, happiness, and anxiety), whether the tweet was political, and if so, to label the ideological

**Outcome**	**Standardized Effect**	**Unstandardized Effect**	**Chronological Mean**	**Engagement Mean**	***p*-value**
**Emotional effects (all tweets)**					
Author Angry	0.5490	0.1710	0.2495	0.4200	0.0002
Author Sad	0.2302	0.0508	0.2285	0.2845	0.0002
Author Anxious	0.2021	0.0283	0.1067	0.1372	0.0004
Author Happy	-0.1158	-0.0670	1.3921	1.3303	0.0012
**Emotional effects (political tweets only)**					
Author Angry	1.4653	0.4565	1.2030	1.6045	0.0002
Author Sad	0.1294	0.0285	0.5129	0.5338	0.4938
Author Anxious	0.1668	0.0233	0.2060	0.2169	0.4408
Author Happy	-0.2244	-0.1299	0.6099	0.4841	0.0066
**Political effects**					
Partisanship	0.1909	0.0283	0.0950	0.1264	0.0002
Out-group Animosity	0.2548	0.0311	0.0627	0.0922	0.0002

Table S42: Average treatment effects on tweet-based outcomes using GPT-4 judgments.

leaning of the tweet and whether the tweet had out-group animosity. All questions were phrased in the same way as the questions asked to our study participants.

Out of the 28,301 unique tweets that we submitted to GPT-4 to label, it returned validly formatted responses for 24,998 tweets. To estimate the average treatment effects displayed in Figure S13, we used only the tweets with valid GPT-4 responses (for both the ATEs based on human labels and those based on GPT-4 labels). This was done to ensure that any observed difference in ATEs between the human and GPT-4 labels was not influenced by using different sets of tweets for each. After filtering to tweets with valid GPT-4 responses, the ATEs were computed through the procedure described in SM section S1.3, simply replacing human labels with GPT-4 labels when relevant.

Below is the prompt given to GPT-4 when a tweet is not a quote tweet or a reply to another tweet.

```
Consider the given tweet by @{author_main}: ‘{main_tweet_text}‘

1. How is @{author_main} feeling in their tweet?
    - Angry
    - Anxious
    - Happy
    - Sad
2. Is @{author_main}’s tweet about a political or social issue?

Answer NULL to the subsequent questions if your response to the previous
question is "No".
3. How does @{author_main}’s tweet lean politically?
4. Is @{author_main}’s tweet expressing anger, frustration, or hostility
    towards a person or group on the Left?
5. Is @{author_main}’s tweet expressing anger, frustration, or hostility
    towards a person or group on the Right?

Respond with a JSON, of the format:
{{
    "author_emotions": {{
        "angry": [VALUE],
```

```
        "anxious": [VALUE],
        "happy": [VALUE],
        "sad": [VALUE]
    }},
    "is_political": [VALUE],
    "political_leaning": [VALUE],
    "animosity_left": [VALUE],
    "animosity_right": [VALUE]
}}
where [VALUE] is an integer key from:
1. {{
        "Not at all": 0,
        "Slightly": 1,
        "Somewhat": 2,
        "Moderately": 3,
        "Extremely": 4
    }}
2. {{"No": 0, "Yes": 1}}
3. {{
        "Far Left": -2,
        "Left": -1,
        "Moderate": 0,
        "Right": 1,
        "Far Right": 2,
        NULL
    }}
4. {{"No": 0, "Yes": 1, NULL}}
5. {{"No": 0, "Yes": 1, NULL}}
```

Below is the prompt used when a tweet is a quote tweet or a reply to another tweet. Like our participants, GPT-4 is given both tweets for context and asked to label both of them. In the following prompt, the variable `other_tweet_type` is either equal to "quote tweet of" or "reply to."

```
Consider the following tweets, where @{author_main}'s tweet is a
{other_tweet_type} @{author_other}'s tweet:
@{author_main}: `{main_tweet_text}`
@{author_other}: `{other_tweet_text}`

Note that there are two tweets.
We will first be asking you questions about @{author_main}'s tweet.
You can use @{author_other}'s tweet for context, but answer the following
questions while focusing on @{author_main}'s tweet.
1. How is @{author_main} feeling in their tweet?
    - Angry
    - Anxious
    - Happy
    - Sad
2. Is @{author_main}'s tweet about a political or social issue?

Answer NULL to the subsequent questions if your response to the previous
question is "No".
3. How does @{author_main}'s tweet lean politically?
4. Is @{author_main}'s tweet expressing anger, frustration, or hostility
towards a person or group on the Left?
```

```
5. Is @{author_main}'s tweet expressing anger, frustration, or hostility
towards a person or group on the Right?

Next, we will be asking you questions about @{author_other}'s tweet.
6. How is @{author_other} feeling in their tweet?
    - Angry
    - Anxious
    - Happy
    - Sad
7. Is @{author_other}'s tweet about a political or social issue?

Answer NULL to the subsequent questions if your response to the previous
question is "No".
8. How does @{author_other}'s tweet lean politically?
9. Is @{author_other}'s tweet expressing anger, frustration, or hostility
towards a person or group on the Left?
10. Is @{author_other}'s tweet expressing anger, frustration, or hostility
towards a person or group on the Right?

Respond with a JSON, of the format:
{{
    "@{author_main}'s tweet": {{
        "author_emotions": {{
            "angry": [VALUE],
            "anxious": [VALUE],
            "happy": [VALUE],
            "sad": [VALUE]
        }},
        "is_political": [VALUE],
        "political_leaning": [VALUE],
        "animosity_left": [VALUE],
        "animosity_right": [VALUE]
    }},
    "@{author_other}'s tweet": {{
        "author_emotions": {{
            "angry": [VALUE],
            "anxious": [VALUE],
            "happy": [VALUE],
            "sad": [VALUE]
        }},
        "is_political": [VALUE],
        "political_leaning": [VALUE],
        "animosity_left": [VALUE],
        "animosity_right": [VALUE]
    }}
}}
where [VALUE] is an integer key from:
1. {{
        "Not at all": 0,
        "Slightly": 1,
        "Somewhat": 2,
        "Moderately": 3,
        "Extremely": 4
    }}
```

```
2. {{"No": 0, "Yes": 1}}
3. {{
        "Far Left": -2,
        "Left": -1,
        "Moderate": 0,
        "Right": 1,
        "Far Right": 2,
        NULL
    }}
4. {{"No": 0, "Yes": 1, NULL}}
5. {{"No": 0, "Yes": 1, NULL}}
6. {{
        "Not at all": 0,
        "Slightly": 1,
        "Somewhat": 2,
        "Moderately": 3,
        "Extremely": 4
    }}
7. {{"No": 0, "Yes": 1}}
8. {{
        "Far Left": -2,
        "Left": -1,
        "Moderate": 0,
        "Right": 1,
        "Far Right": 2,
        NULL
    }}
9. {{"No": 0, "Yes": 1, NULL}}
10. {{"No": 0, "Yes": 1, NULL}}
```

S4.6 Heterogeneous effects

Here, we explore how the effects shown in Figure 1 change when restricting our analysis to subpopulations of users, among demographic lines (or using other of our survey questions). We subset users the demographic subgroups described in SM section S2, and additionally by two survey questions (the users' self-reported main reason for using Twitter and main category of content seen during the user study):

- User political leaning ("Political Leaning")
- User political party ("Political Party")
- User age group ("Age")
- User gender ("Gender")
- User race ("Race")
- User ethicity ("Ethnicity")
- User education level ("Education Level")
- User annual household income ("Household Income")
- User primary reason for using Twitter ("Why Twitter")
- User category of content seen ("Primary Category of Content")

For all questions we consider here that allow multiple answers (race, ethnicity, category of content), we double-count participants as being present in each subpopulation that they selected.

The phrasings of all questions, and answer formats can be found in SM section S5. For easier reference we include below that of the non-demographic survey questions.

We ask participants about the primary reason they use Twitter as follows: "What would you say is the main reason you use Twitter?" The options to select from are: "A way to stay informed," "Entertainment," "Keeping me connected to other people," "It's useful for my job or school," "Let's me see different points of view," "A way to express my opinions."

We ask participants about the content shown to them as follows: "What were the tweets we showed you today predominantly about? Select a maximum of two." The options to select from are "News," "Politics," "Work," "Entertainment," "Hobbies."

We report these effects by first displaying them for each outcome (SM section S4.6.1) and then for each demographic attribute or survey question response (SM section S4.6.2).

S4.6.1 Outcome effects for each subgroup, grouped by outcome

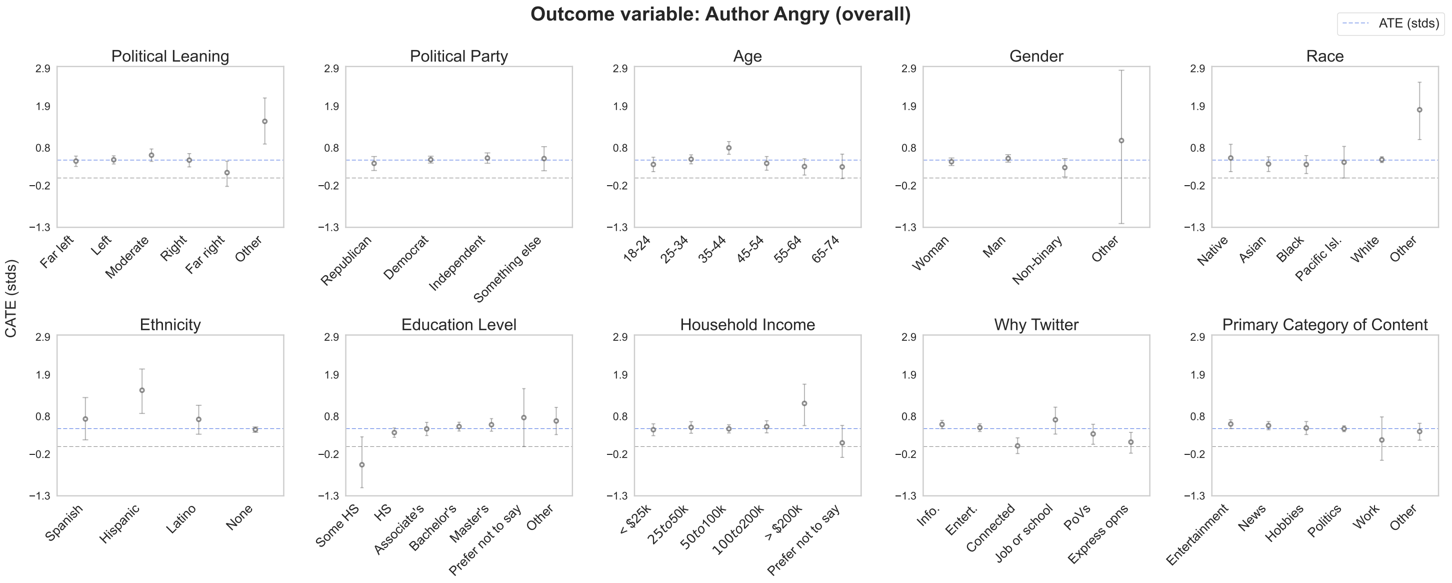

Figure S14: Conditional average treatment effect (CATE) across subgroups for the outcome variable 'author angry.' The blue line shows the average treatment effect (ATE).

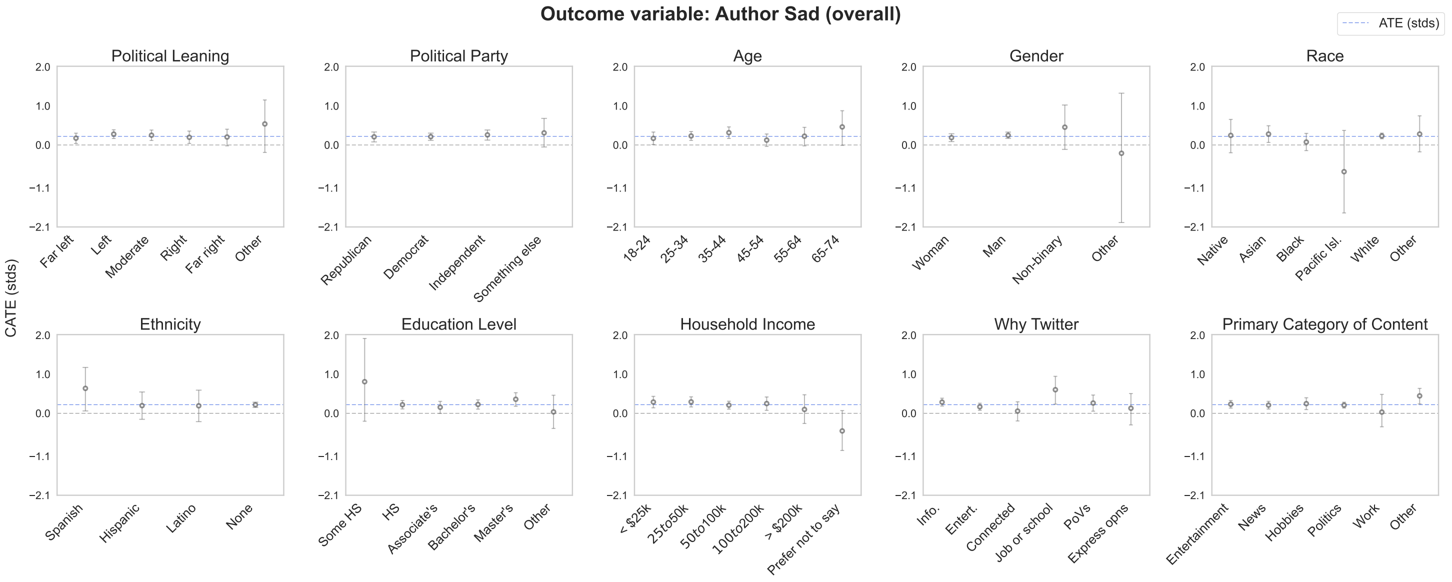

Figure S15: Conditional average treatment effect (CATE) across subgroups for the outcome variable 'author sad.' The blue line shows the average treatment effect (ATE).

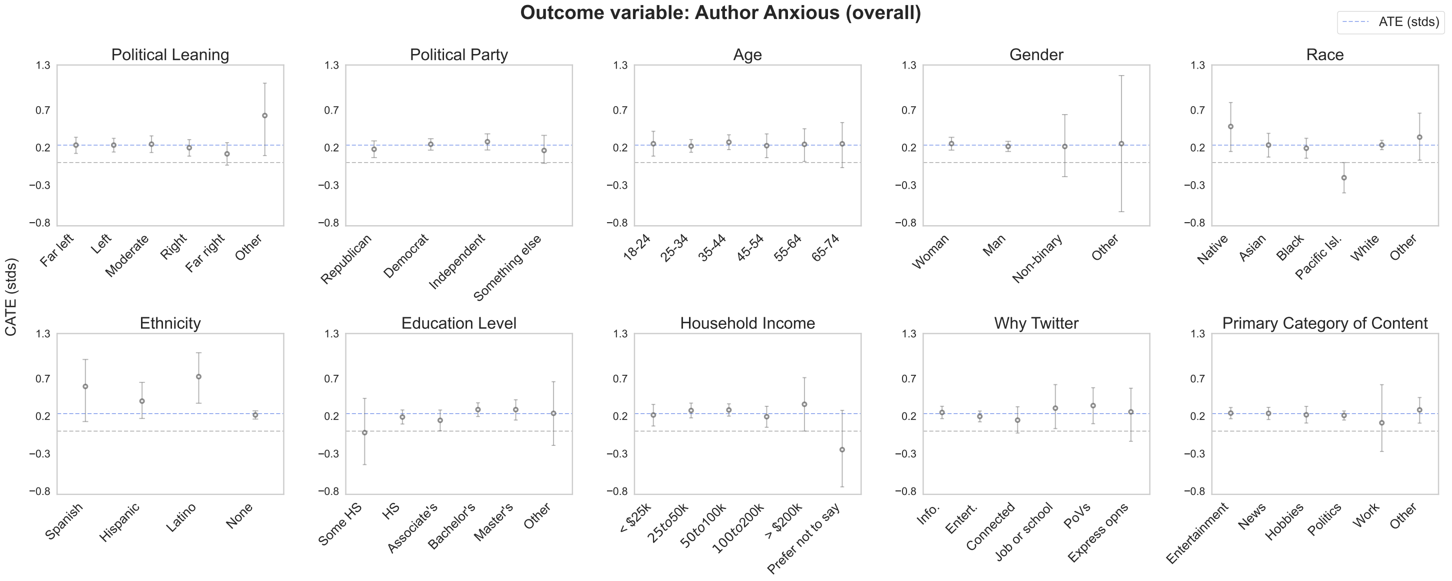

Figure S16: Conditional average treatment effect (CATE) across subgroups for the outcome variable 'author anxious.' The blue line shows the average treatment effect (ATE).

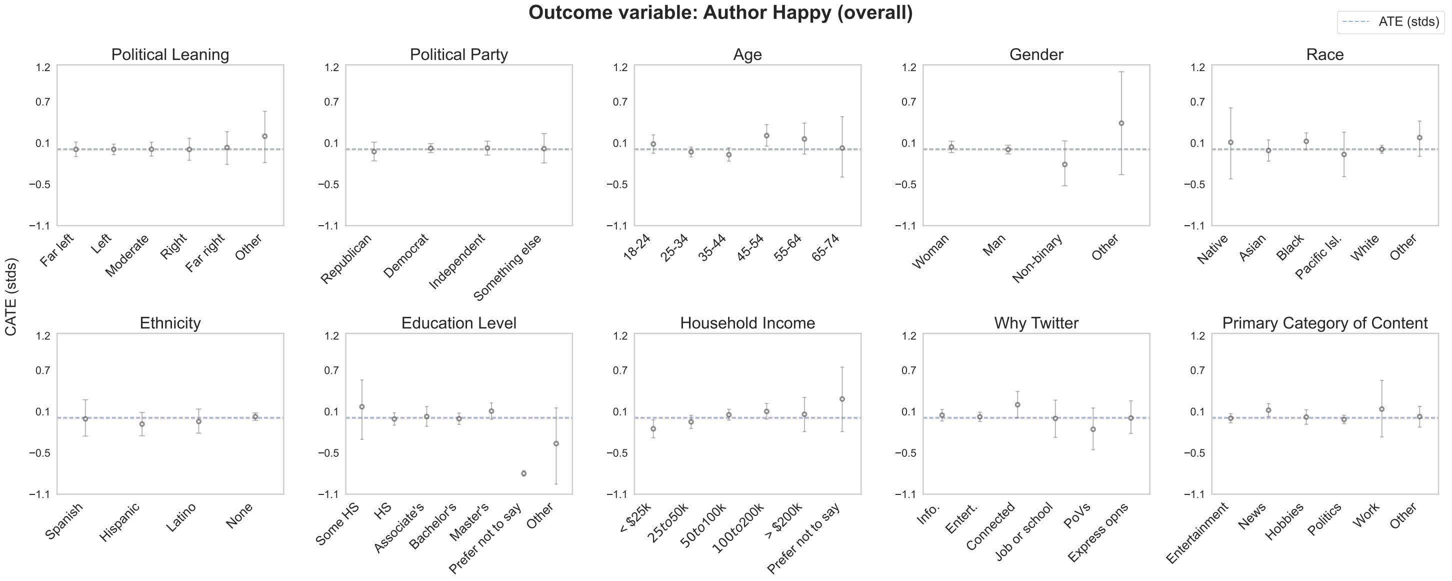

Figure S17: Conditional average treatment effect (CATE) across subgroups for the outcome variable 'author happy.' The blue line shows the average treatment effect (ATE).

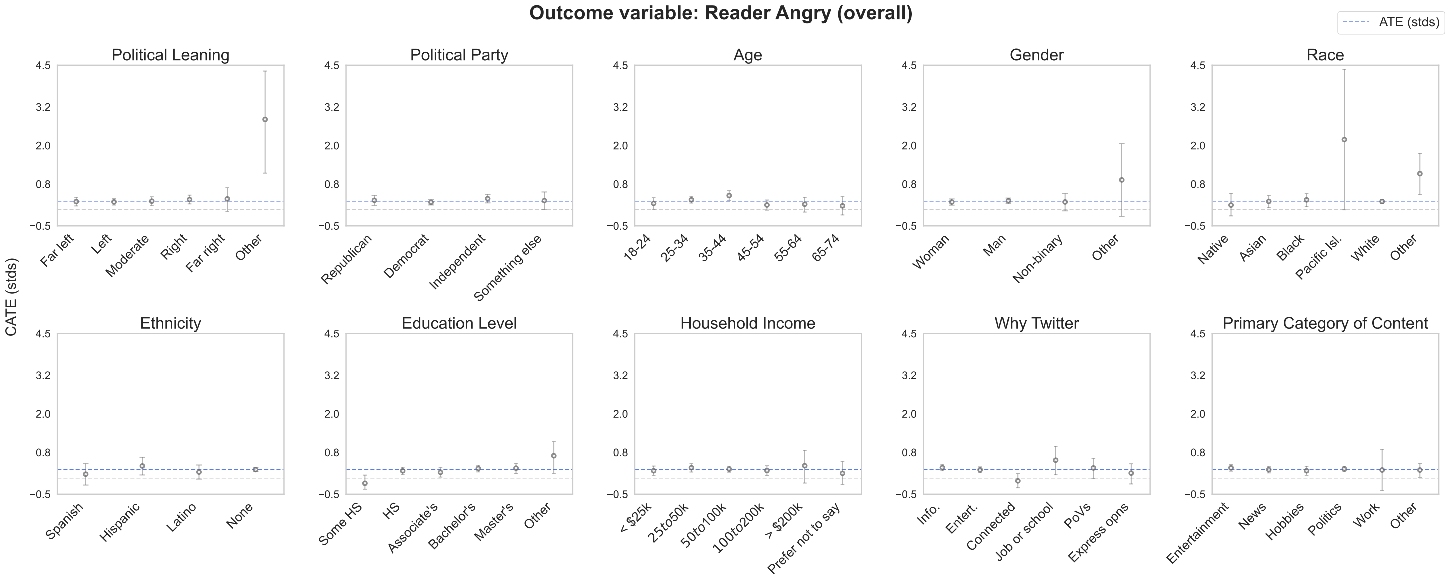

Figure S18: Conditional average treatment effect (CATE) across subgroups for the outcome variable 'reader angry (overall)'. The blue line shows the average treatment effect (ATE).

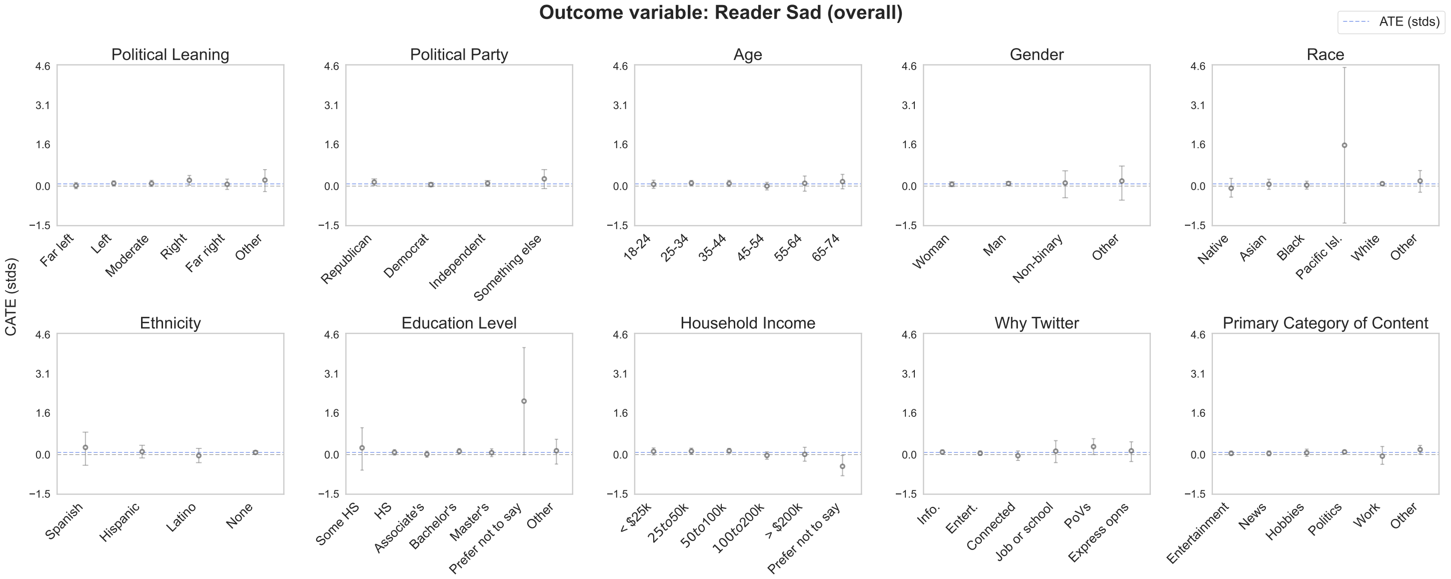

Figure S19: Conditional average treatment effect (CATE) across subgroups for the outcome variable 'reader sad (overall).' The blue line shows the average treatment effect (ATE).

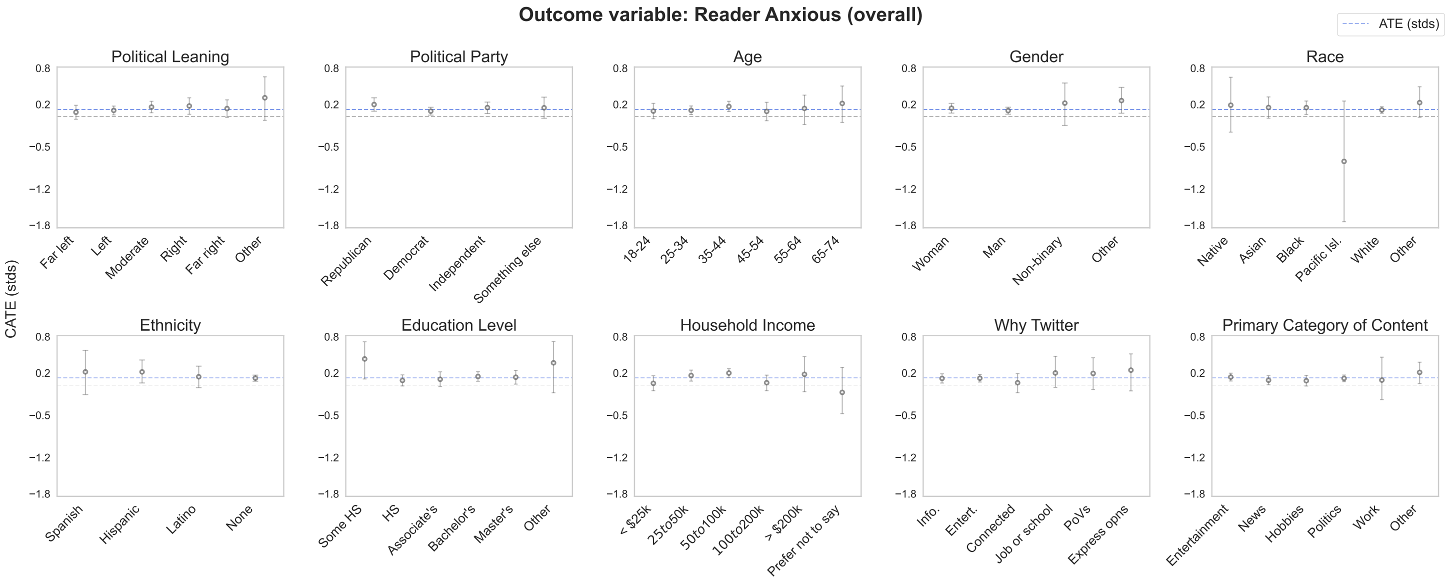

Figure S20: Conditional average treatment effect (CATE) across subgroups for the outcome variable 'reader anxious (overall).' The blue line shows the average treatment effect (ATE).

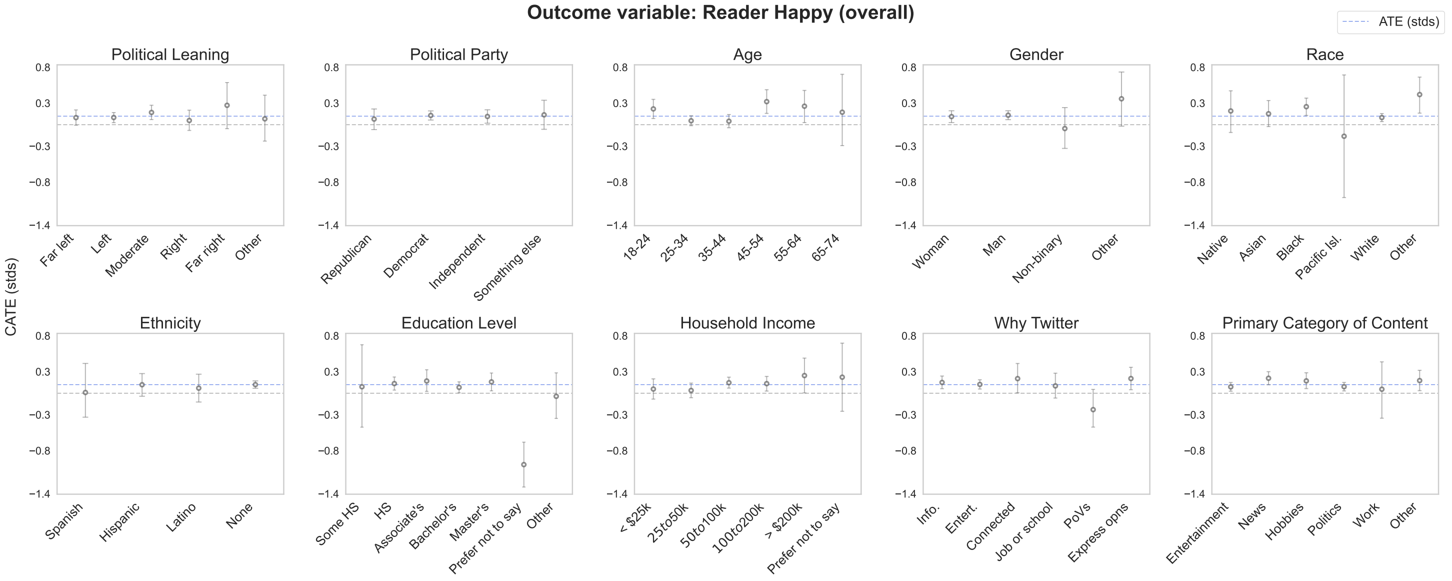

Figure S21: Conditional average treatment effect (CATE) across subgroups for the outcome variable 'reader happy (overall).' The blue line shows the average treatment effect (ATE).

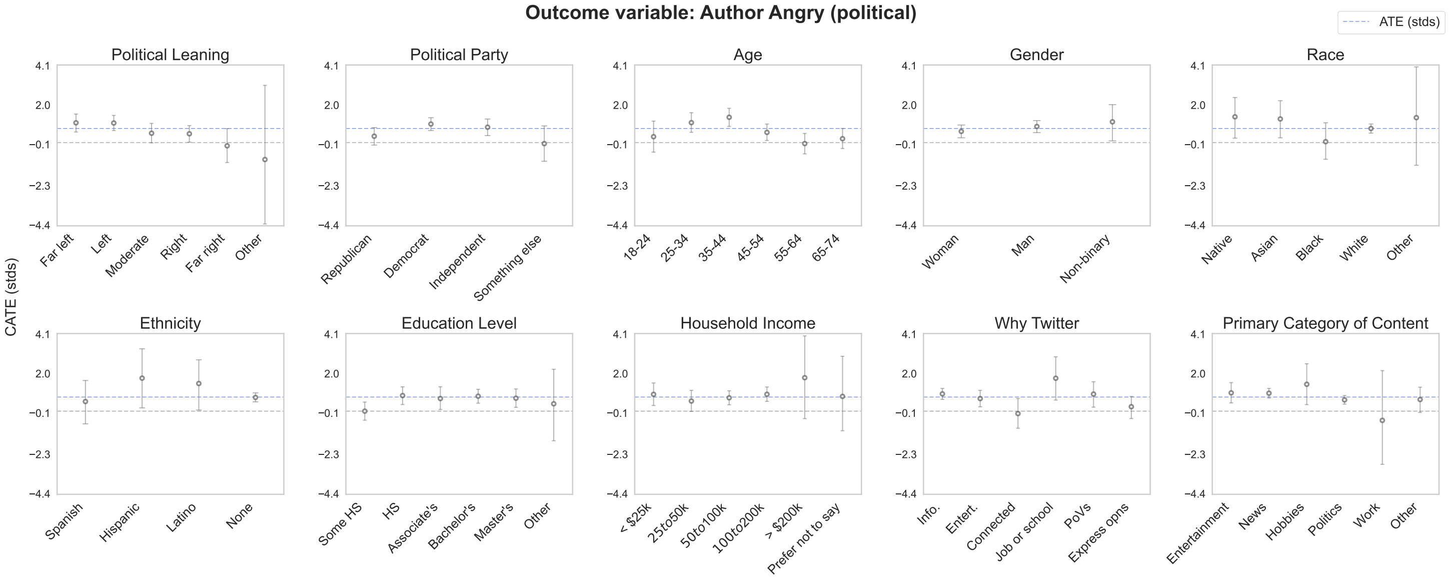

Figure S22: Conditional average treatment effect (CATE) across subgroups for the outcome variable 'author angry (political).' The blue line shows the average treatment effect (ATE).

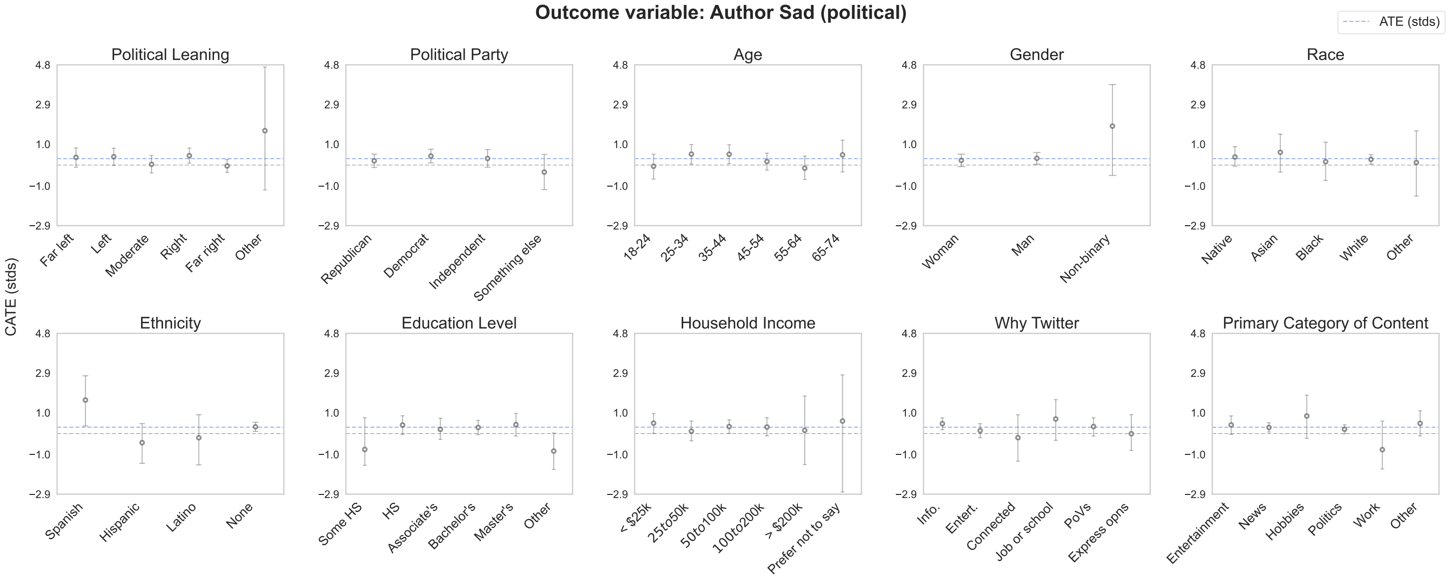

Figure S23: Conditional average treatment effect (CATE) across subgroups for the outcome variable 'author sad (political).' The blue line shows the average treatment effect (ATE).

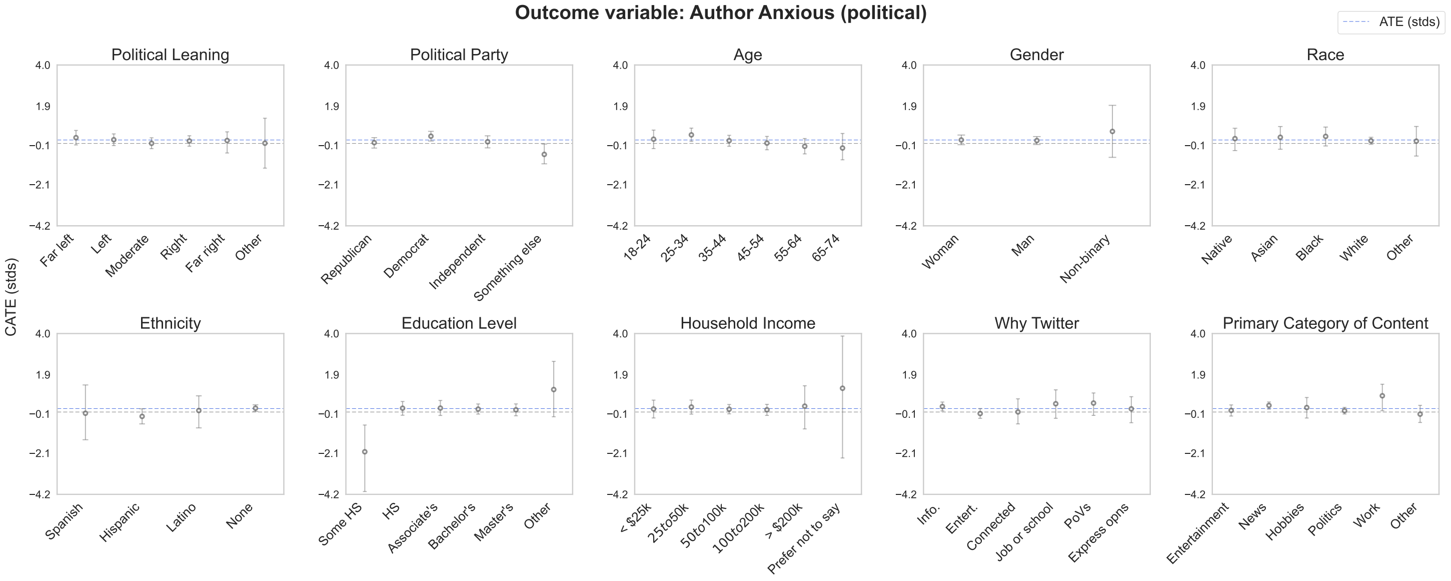

Figure S24: Conditional average treatment effect (CATE) across subgroups for the outcome variable 'author anxious (political).' The blue line shows the average treatment effect (ATE).

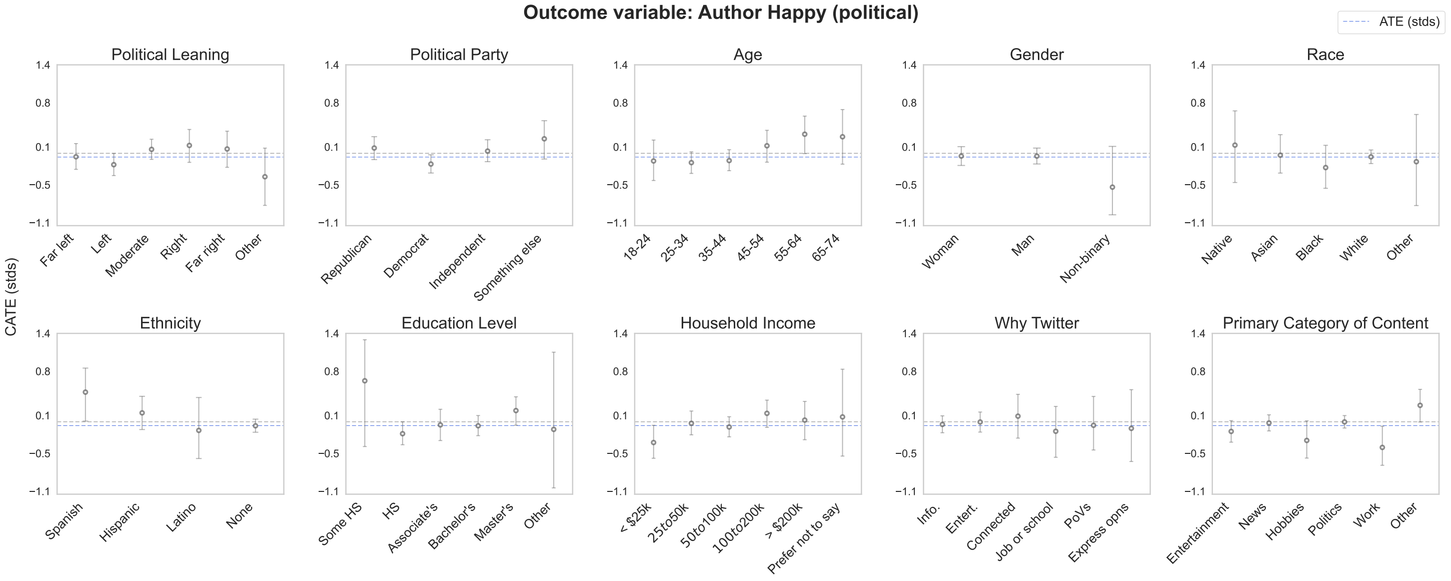

Figure S25: Conditional average treatment effect (CATE) across subgroups for the outcome variable 'author happy (political).' The blue line shows the average treatment effect (ATE).

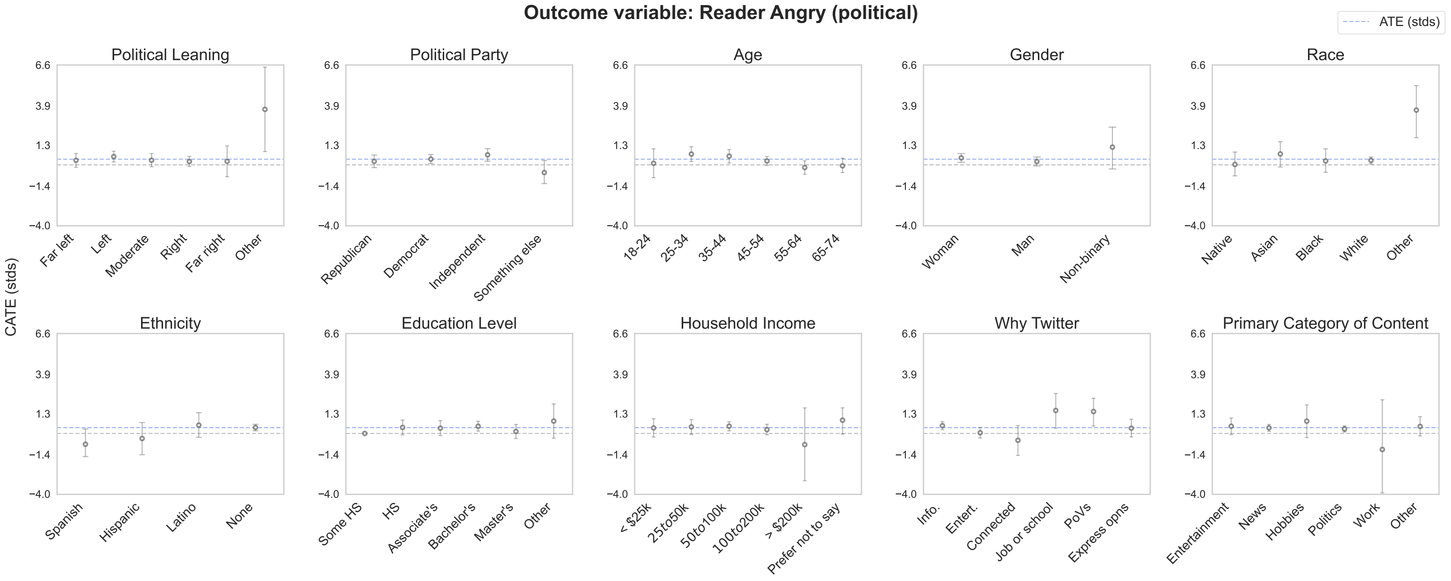

Figure S26: Conditional average treatment effect (CATE) across subgroups for the outcome variable 'reader angry (political).' The blue line shows the average treatment effect (ATE).

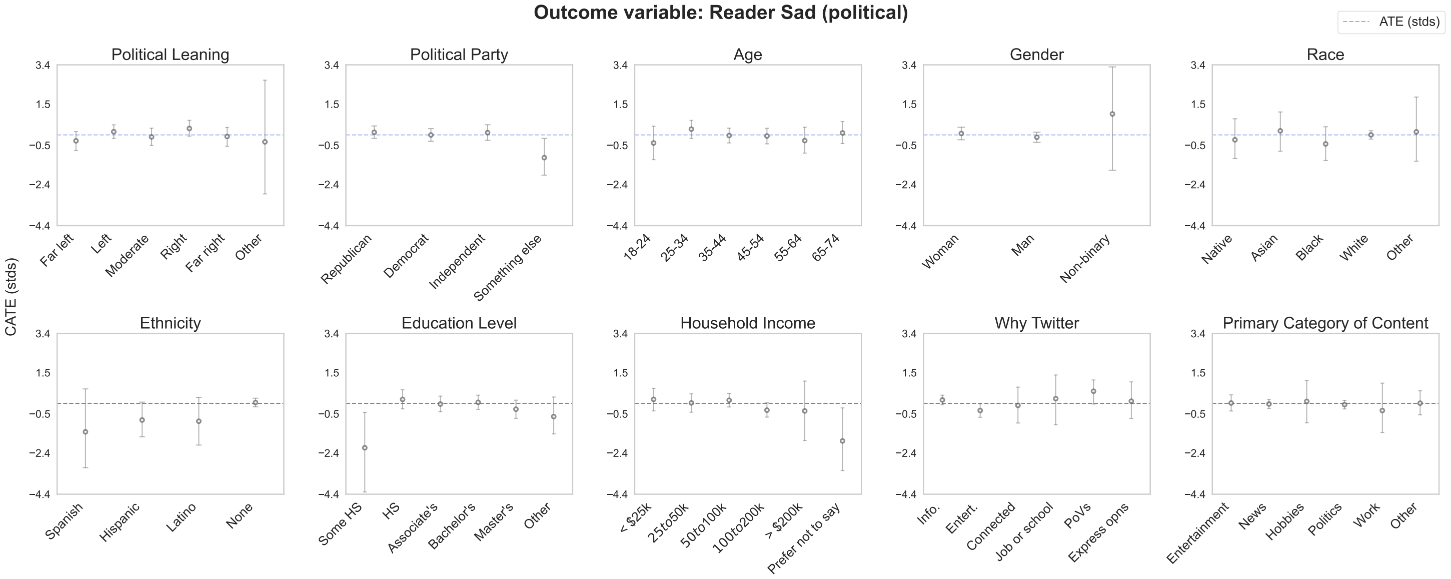

Figure S27: Conditional average treatment effect (CATE) across subgroups for the outcome variable 'reader sad (political).' The blue line shows the average treatment effect (ATE).

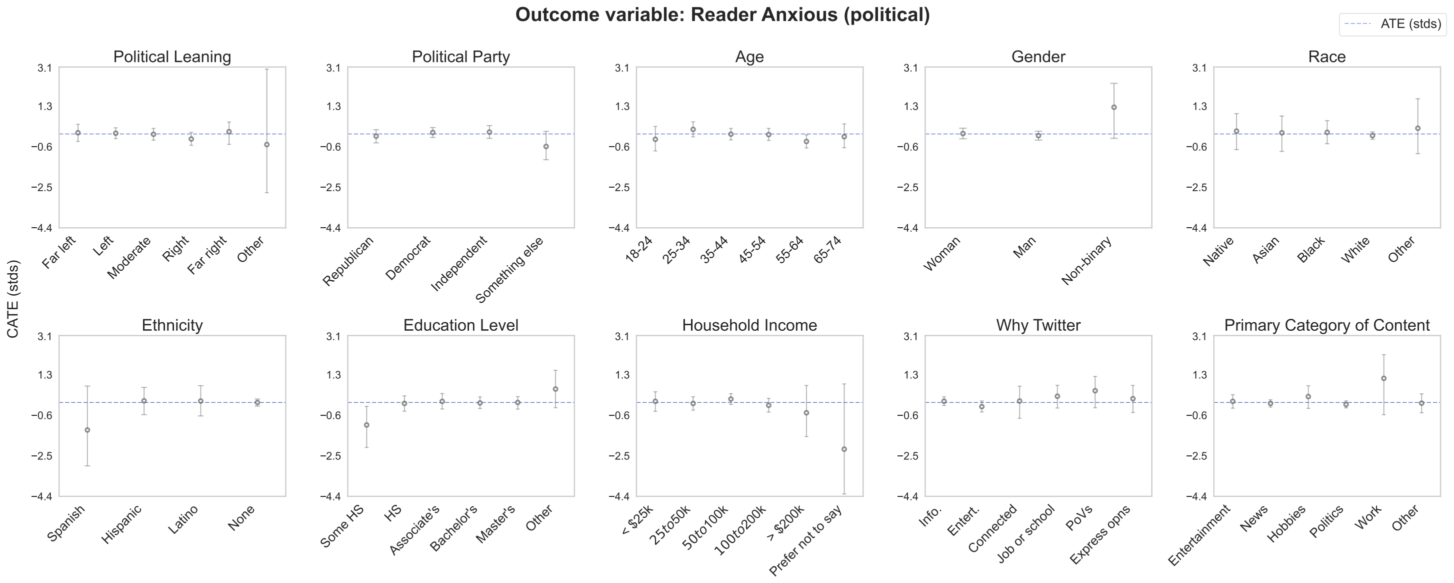

Figure S28: Conditional average treatment effect (CATE) across subgroups for the outcome variable 'reader anxious (political).' The blue line shows the average treatment effect (ATE).

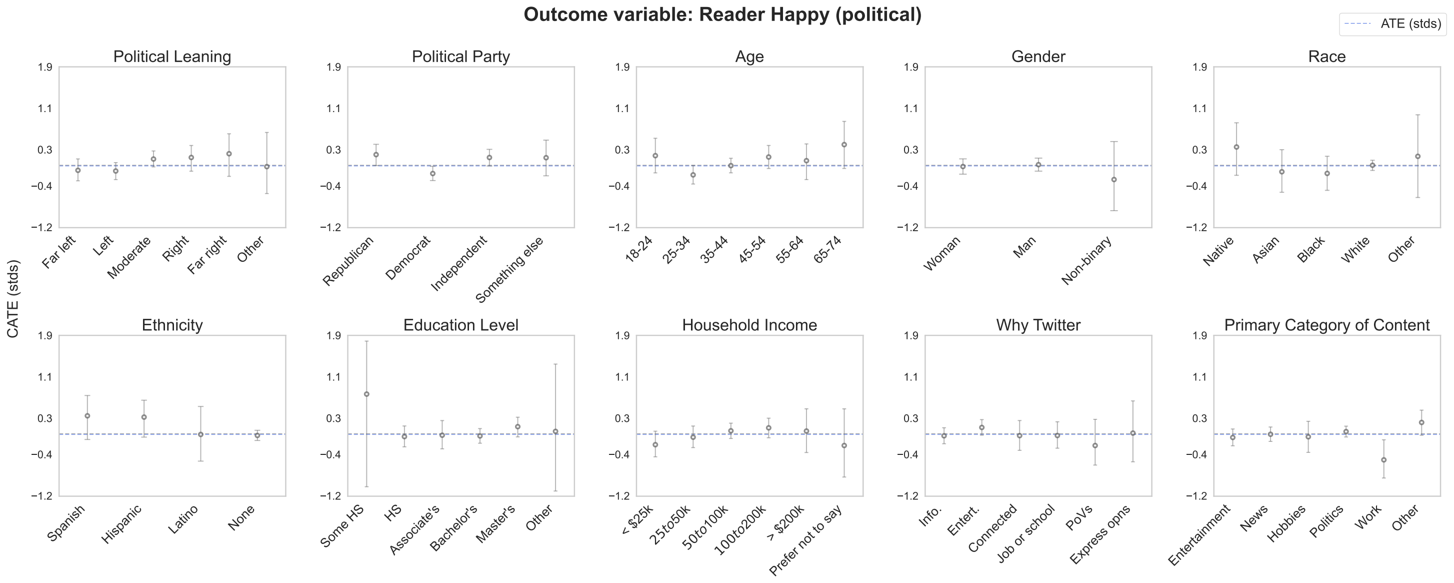

Figure S29: Conditional average treatment effect (CATE) across subgroups for the outcome variable 'reader happy (political).' The blue line shows the average treatment effect (ATE).

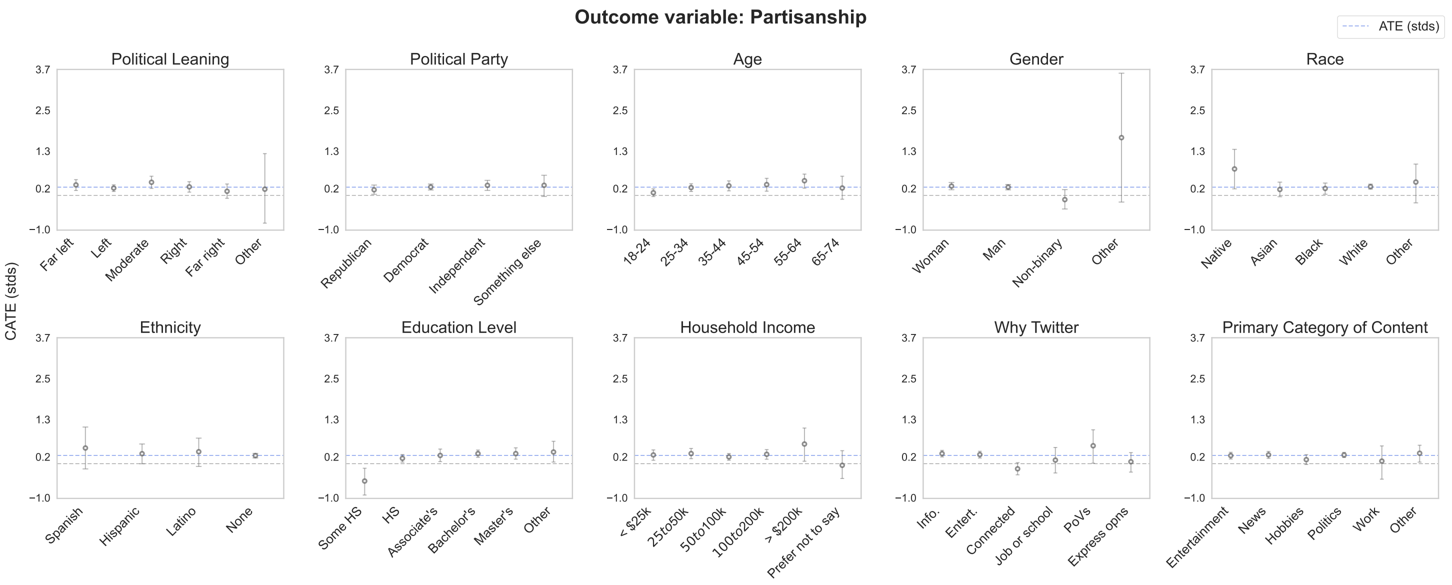

Figure S30: Conditional average treatment effect (CATE) for the outcome variable ‘partisanship.’ The blue line shows the average treatment effect (ATE).

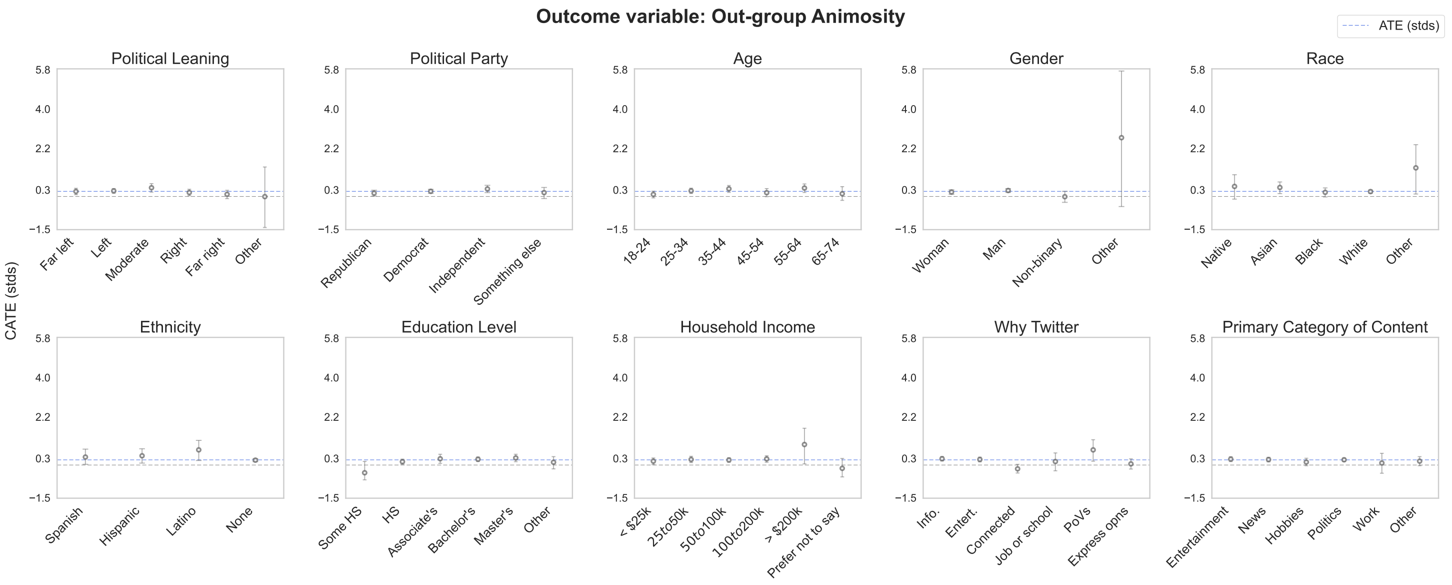

Figure S31: Conditional average treatment effect (CATE) for the outcome variable ‘out-group animosity.’ The blue line shows the average treatment effect (ATE).

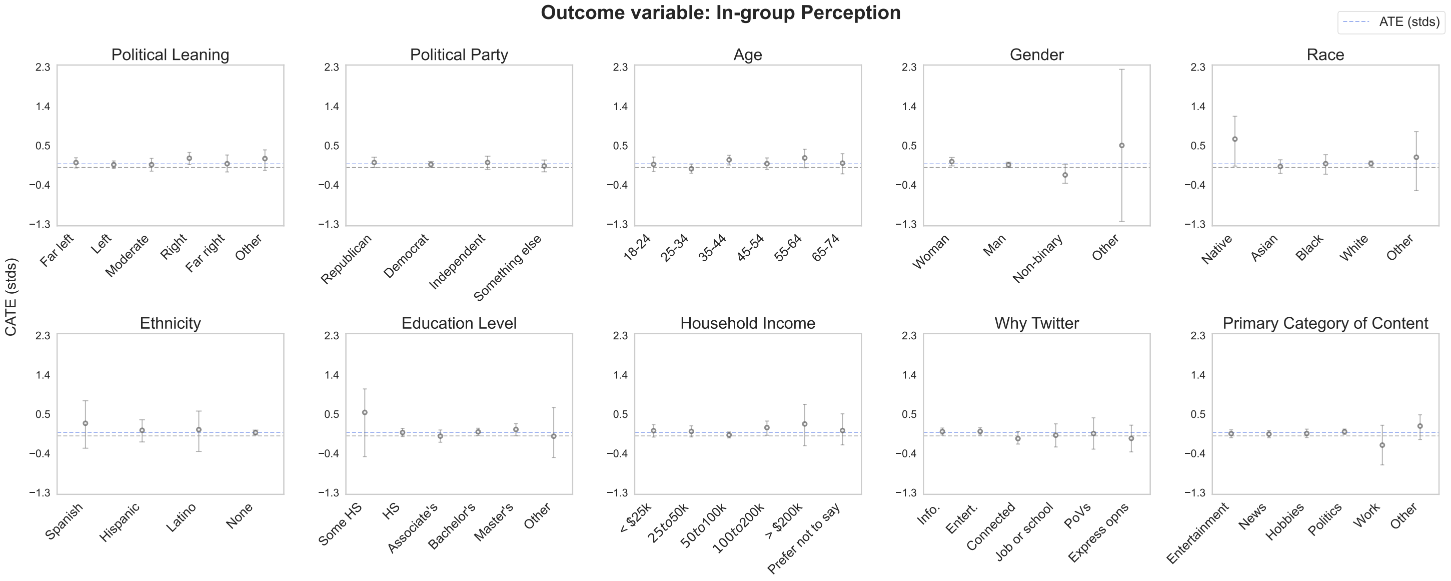

Figure S32: Conditional average treatment effect (CATE) for the outcome variable 'in-group perception.' The blue line shows the average treatment effect (ATE).

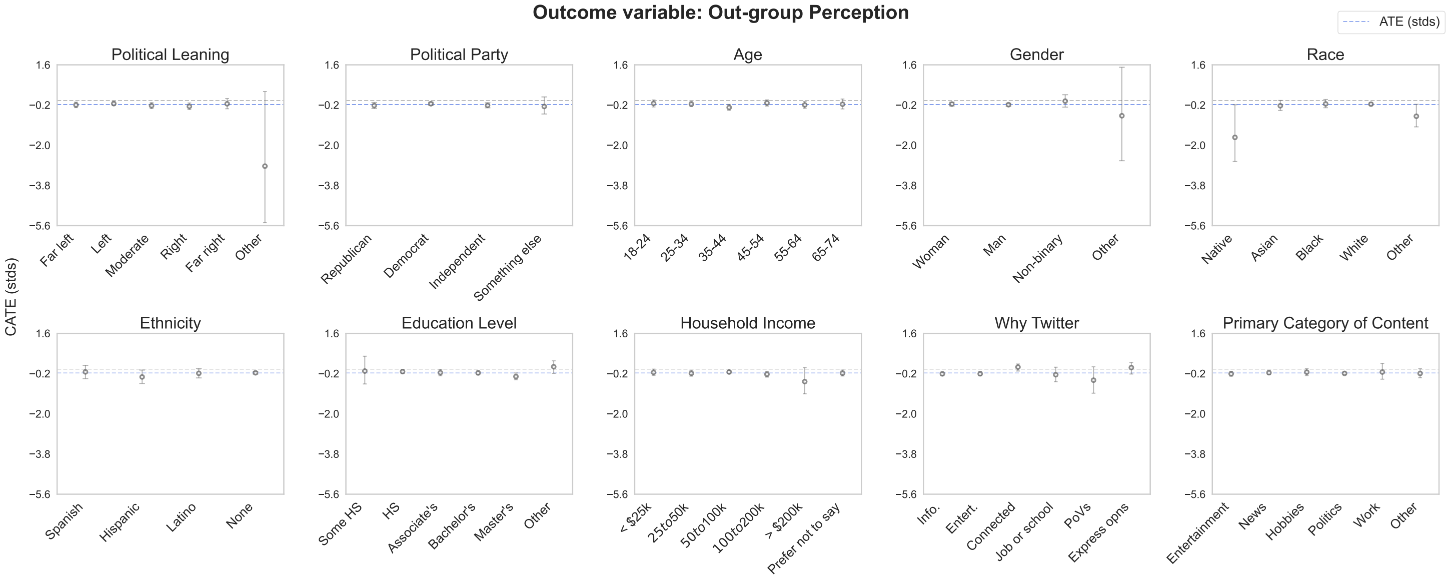

Figure S33: Conditional average treatment effect (CATE) for the outcome variable 'out-group perception.' The blue line shows the average treatment effect (ATE).

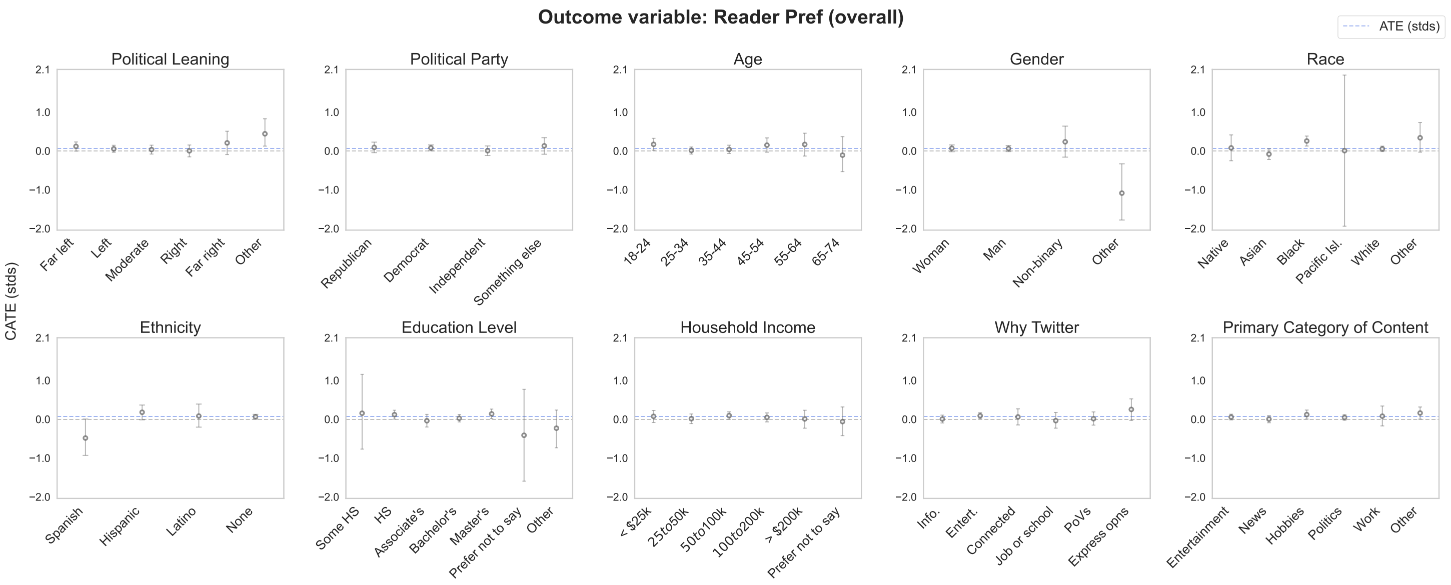

Figure S34: Conditional average treatment effect (CATE) across subgroups for the outcome variable 'reader preference (overall).' The blue line shows the average treatment effect (ATE).

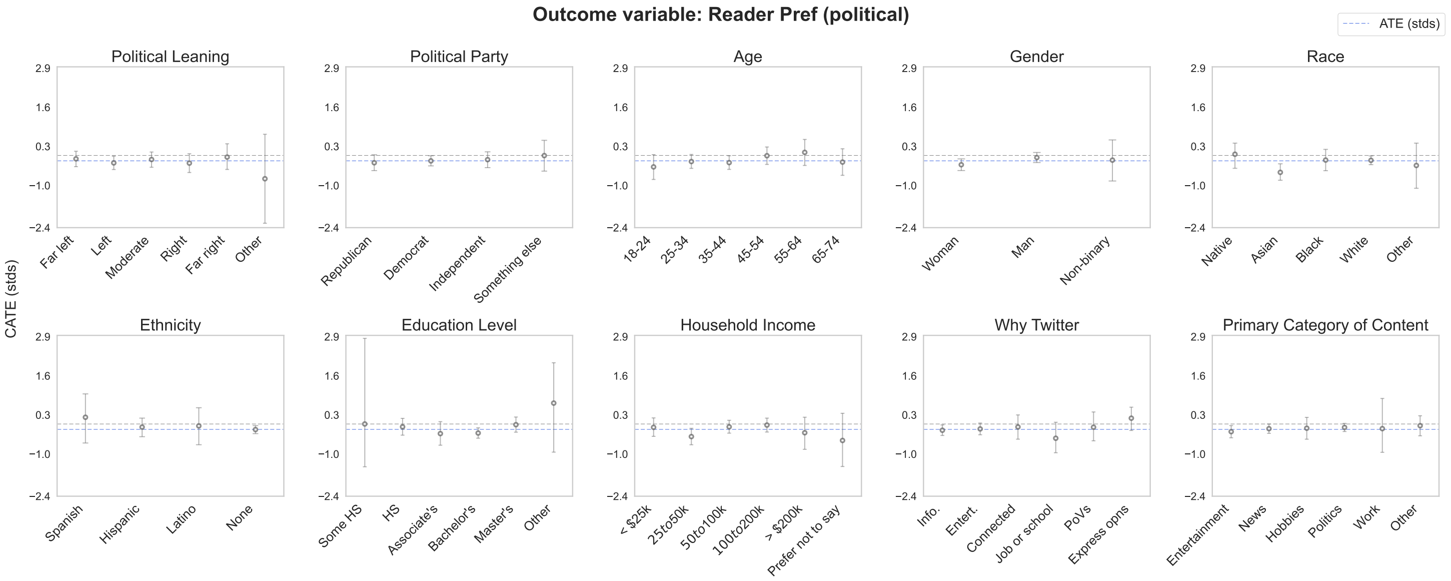

Figure S35: Conditional average treatment effect (CATE) across subgroups for the outcome variable 'reader preference (political).' The blue line shows the average treatment effect (ATE).

S4.6.2 Outcome effects for each subgroup, grouped by subgroup

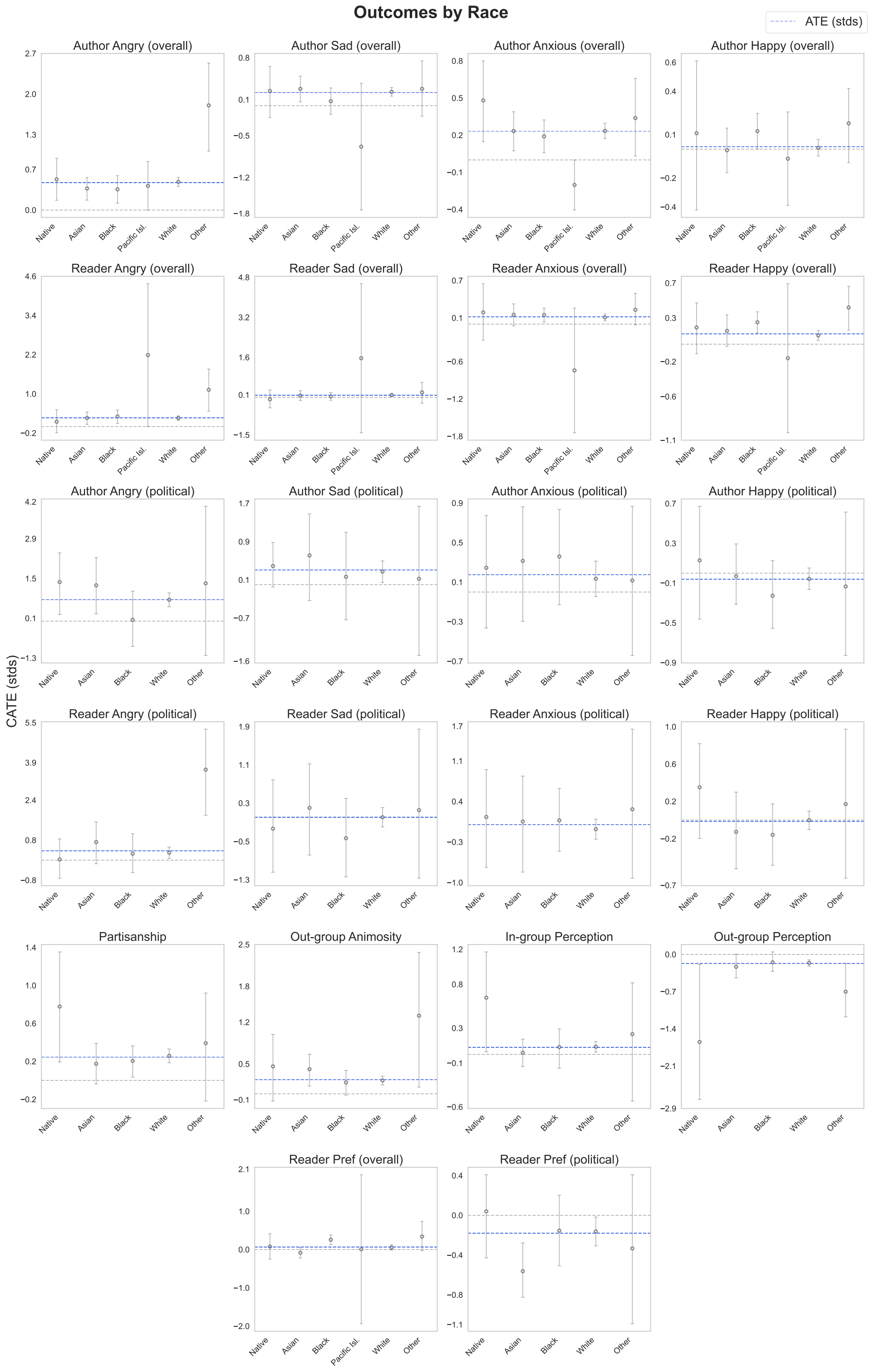

Figure S36: Conditional average treatment effect (CATE) for all outcomes when conditioned on different races. The blue line shows the average treatment effect (ATE).

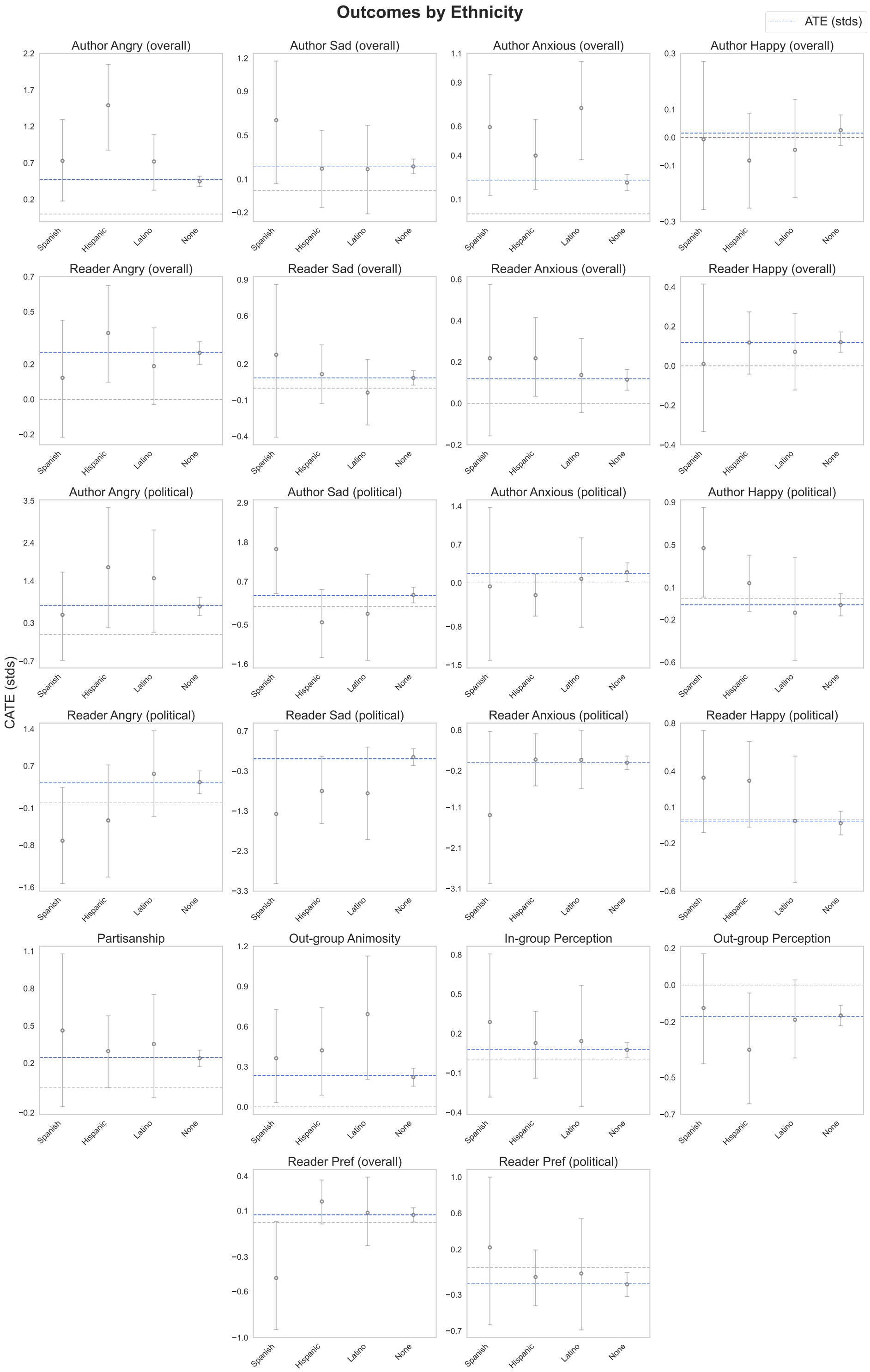

Figure S37: Conditional average treatment effect (CATE) for all outcomes when conditioned on different ethnicities. The blue line shows the average treatment effect (ATE).

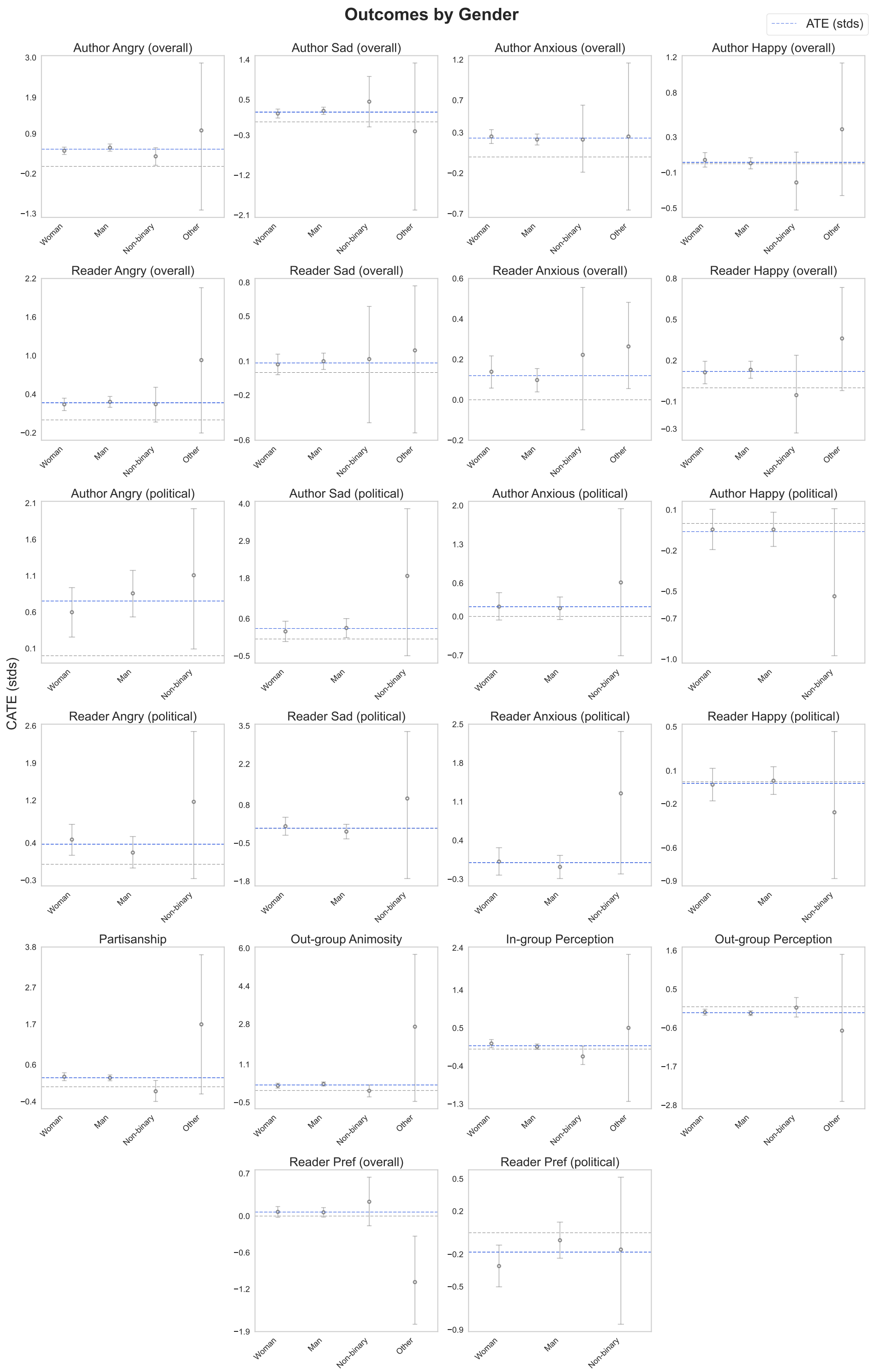

Figure S38: Conditional average treatment effect (CATE) for all outcomes when conditioned on different genders. The blue line shows the average treatment effect (ATE).

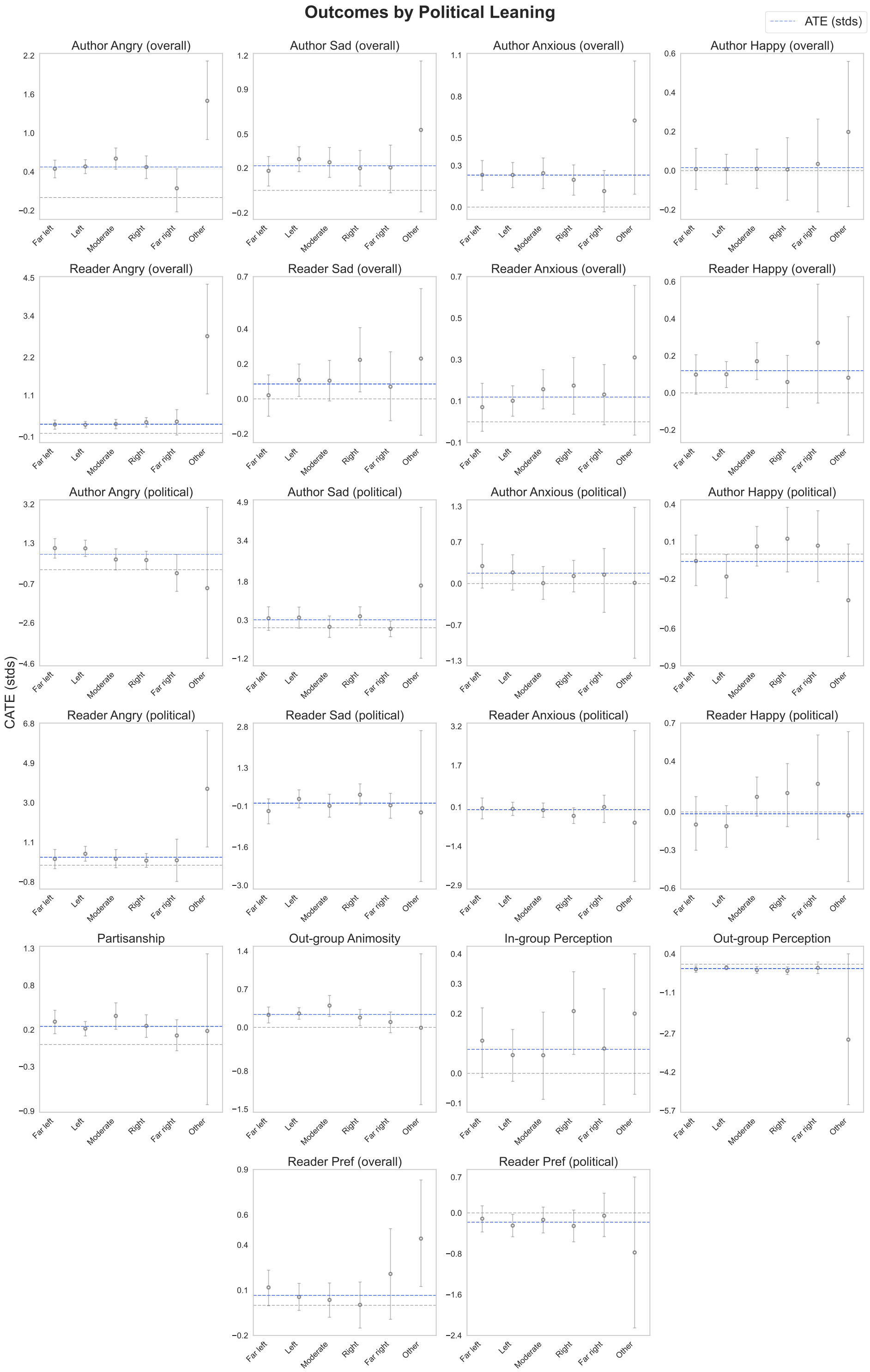

Figure S39: Conditional average treatment effect (CATE) for all outcomes when conditioned on different ideological political leanings. The blue line shows the average treatment effect (ATE).

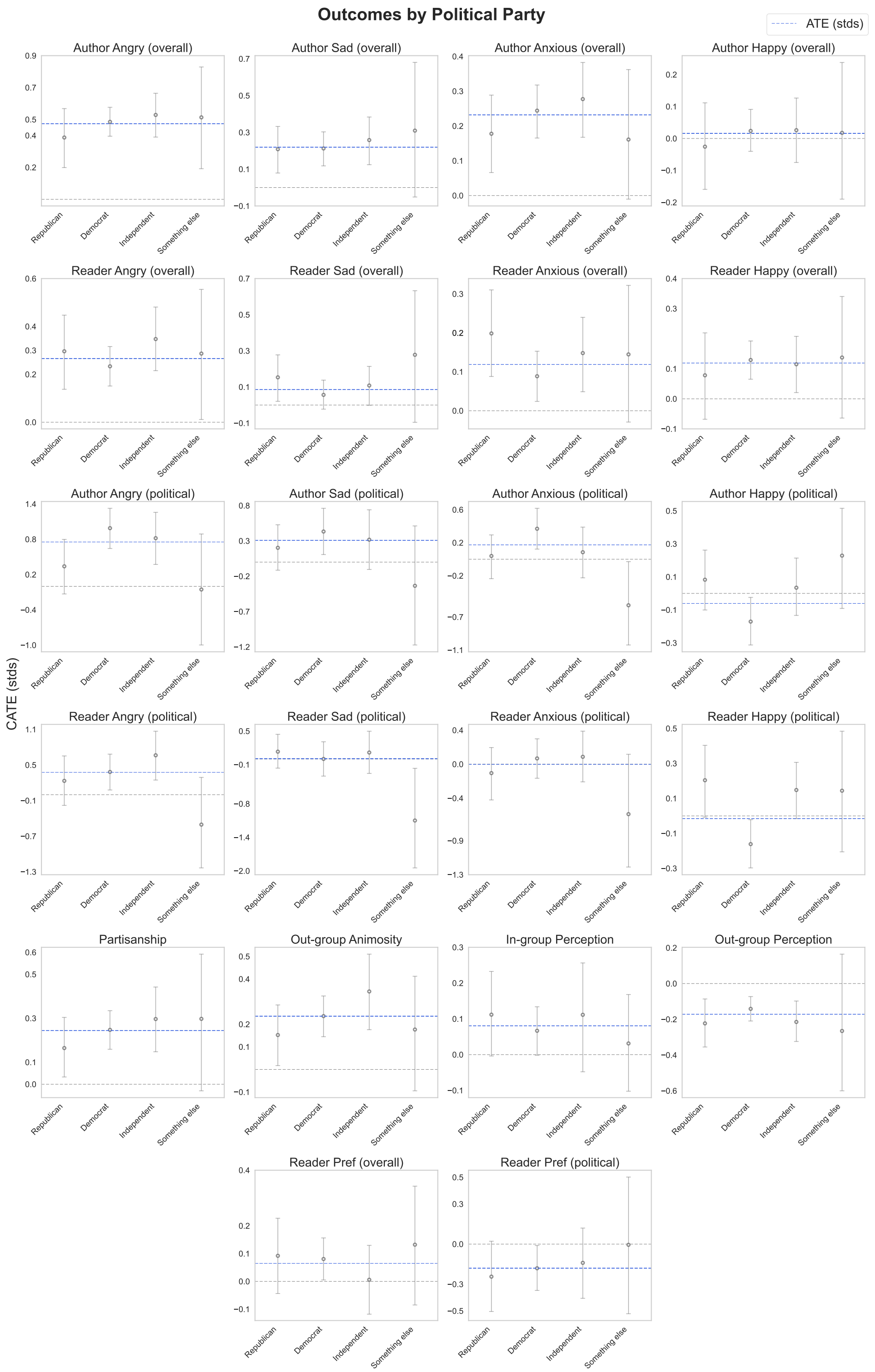

Figure S40: Conditional average treatment effect (CATE) for all outcomes when conditioned on different ideological political parties. The blue line shows the average treatment effect (ATE).

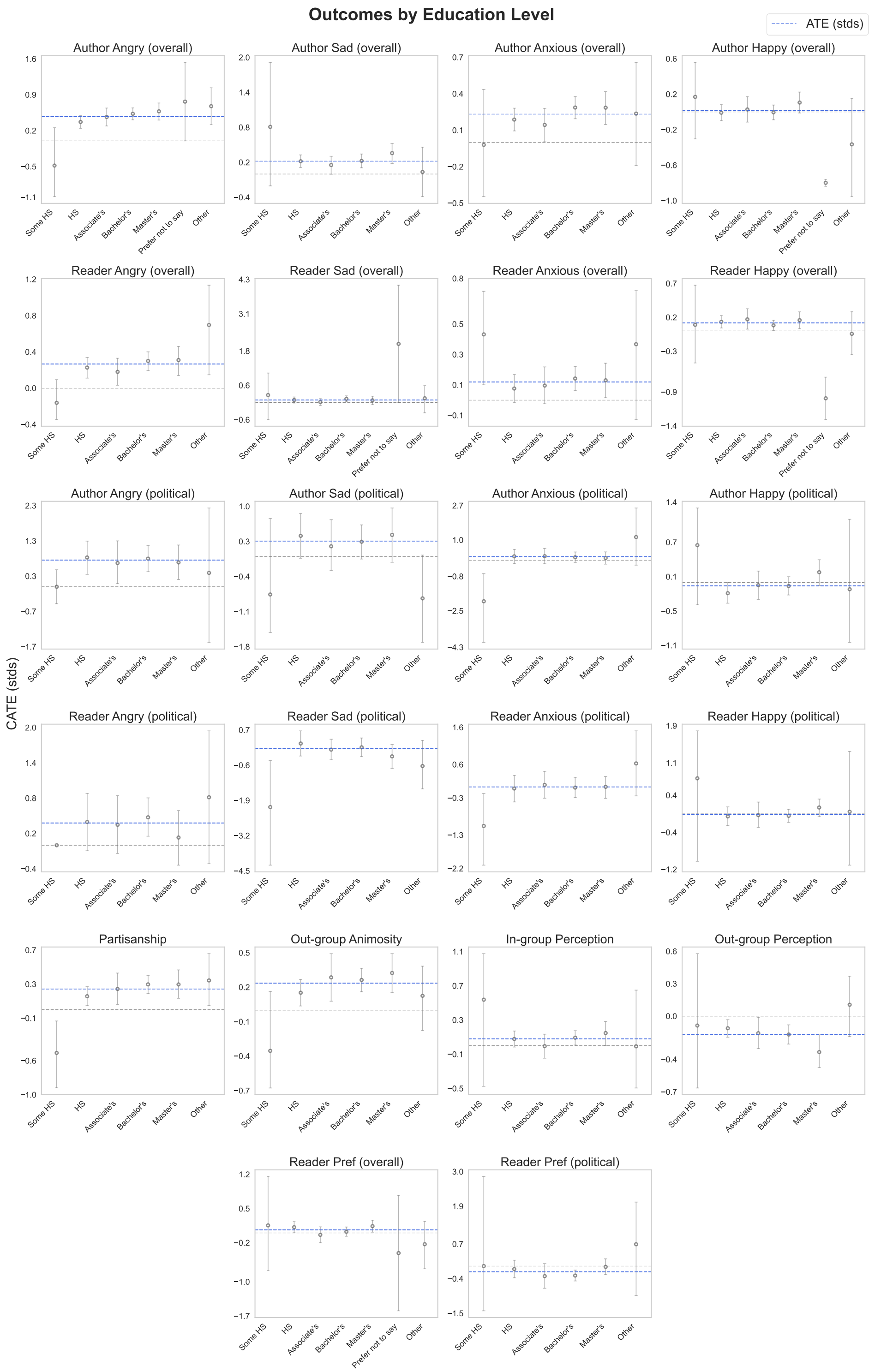

Figure S41: Conditional average treatment effect (CATE) for all outcomes when conditioned on different education levels. The blue line shows the average treatment effect (ATE).

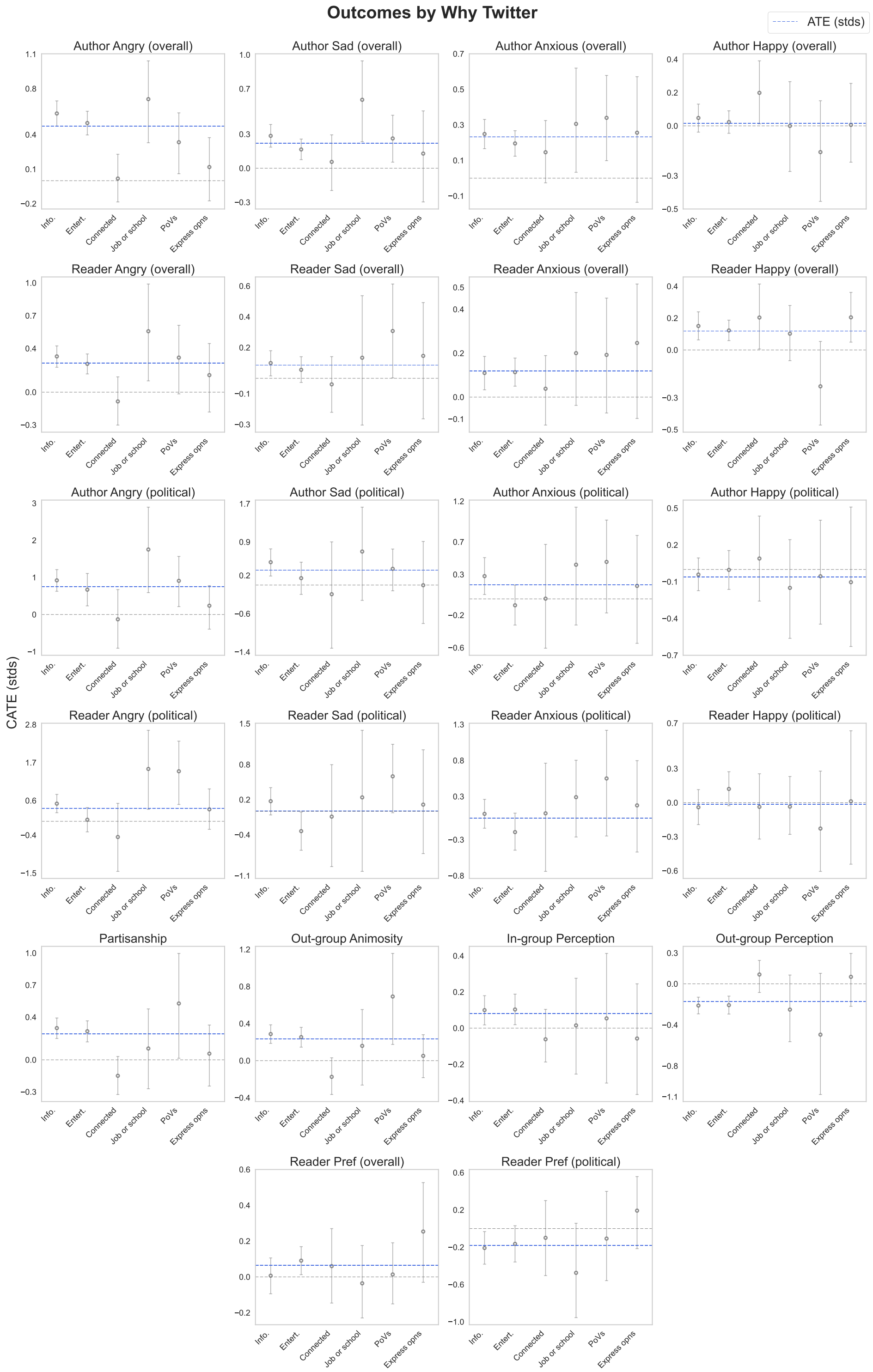

Figure S42: Conditional average treatment effect (CATE) for all outcomes when conditioned on different main reasons for using Twitter. The blue line shows the average treatment effect (ATE). We ask participants about the main reason they use Twitter as follows: "What would you say is the main reason you use Twitter?" (SM section S5). The options to select from are: "A way to stay informed," "Entertainment" "Keeping me connected to other people," "It's useful for my job or school," "Let's me see different points of view," "A way to express my opinions".

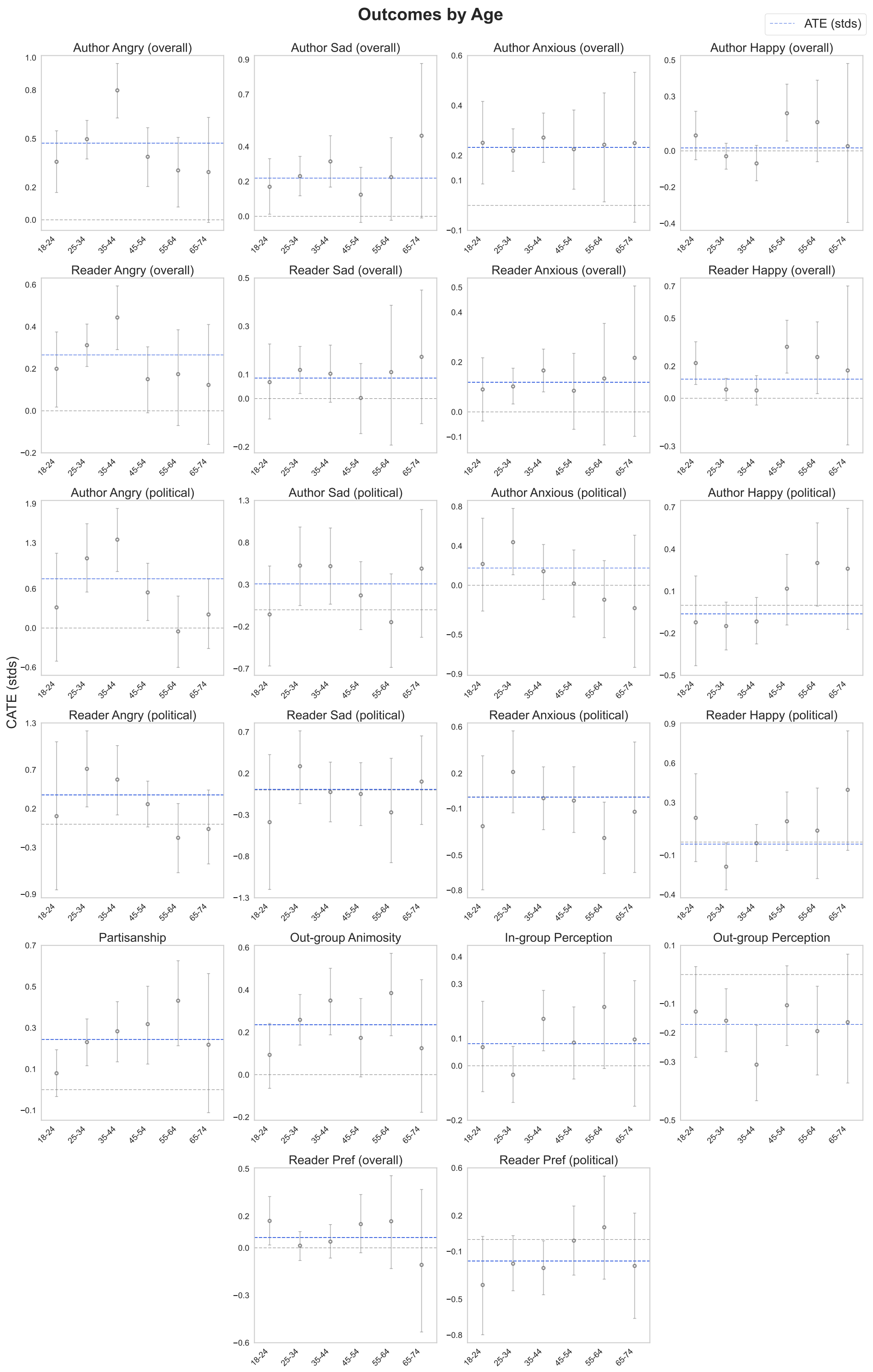

Figure S43: Conditional average treatment effect (CATE) for all outcomes when conditioned on different age groups. The blue line shows the average treatment effect (ATE).

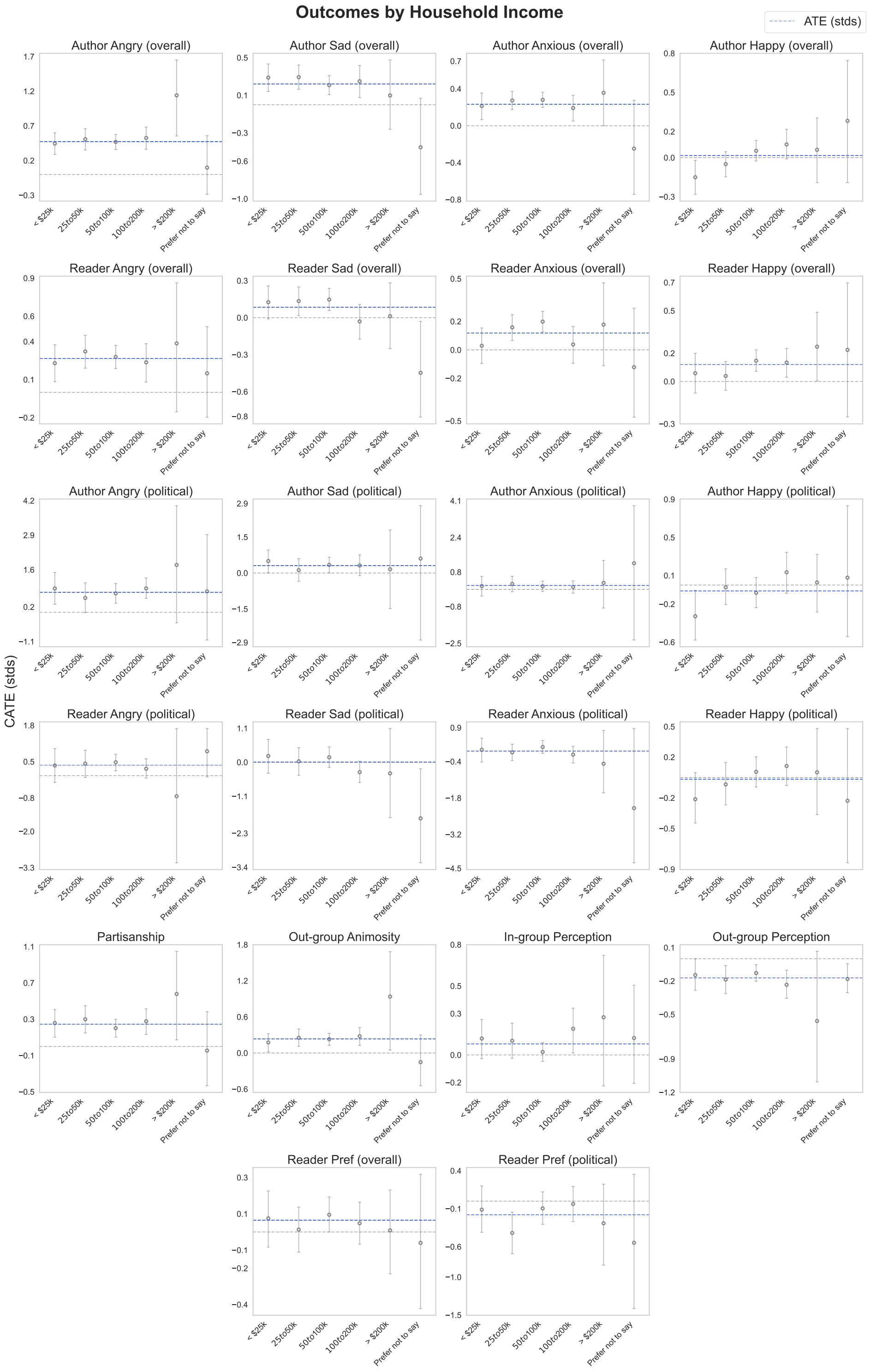

Figure S44: Conditional average treatment effect (CATE) for all outcomes when conditioned on different annual household income levels. The blue line shows the average treatment effect (ATE).

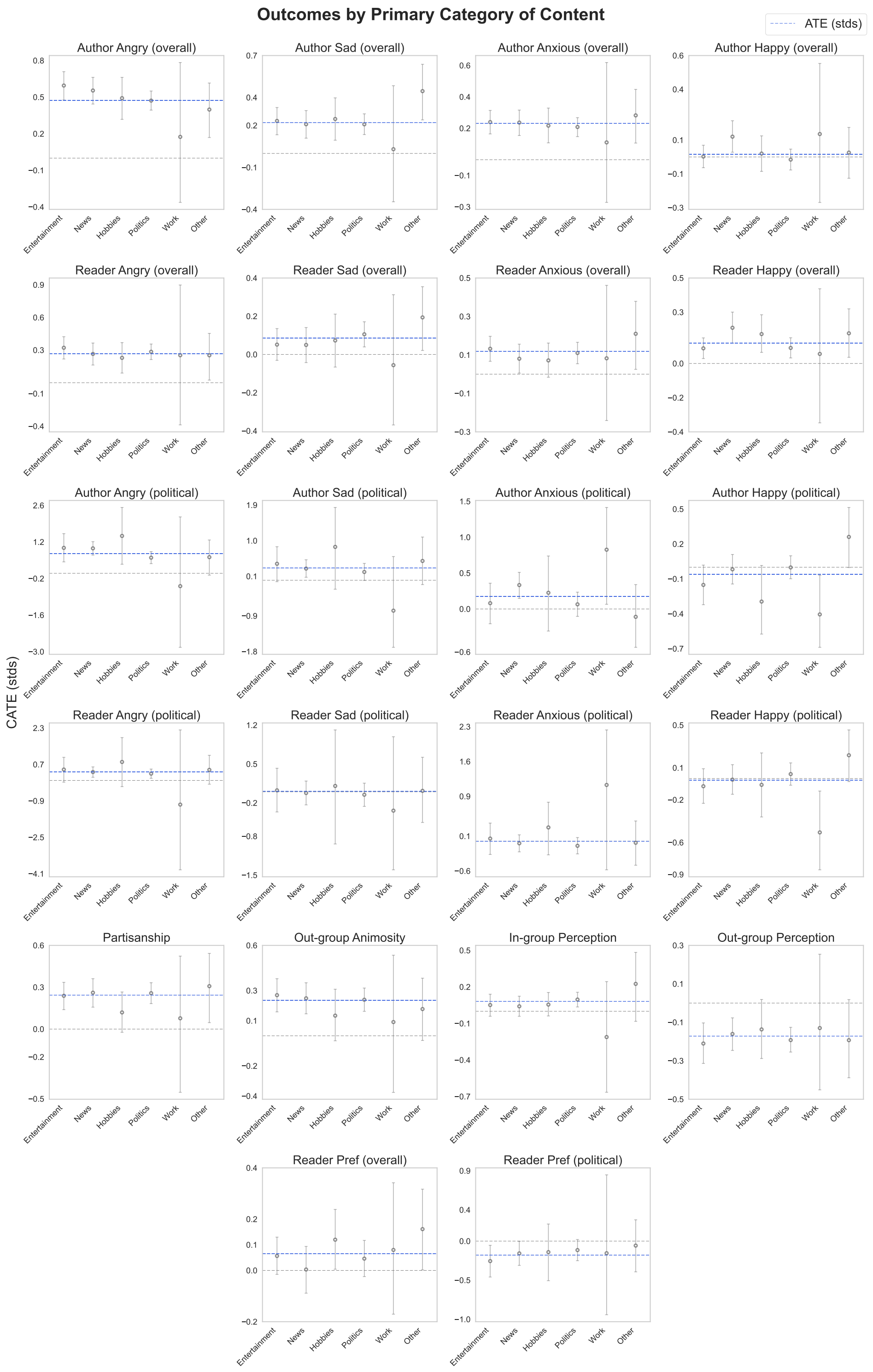

Figure S45: Conditional average treatment effect (CATE) for all outcomes when conditioned on different types of primary content category shown. The blue line shows the average treatment effect (ATE). We ask participants about the content shown to them as follows: "What were the tweets we showed you today predominantly about? Select a maximum of two" (SM section S5). The options to select from are "News," "Politics," "Work," "Entertainment," "Hobbies."

S4.7 Effects of stated preference timeline

Outcome	Standardized Effect	Unstandardized Effect	Chron. Mean	Eng. Mean	p-value
**Emotional effects (all tweets)**					
Author Angry	0.110	0.044	0.352	0.402	0.0002
Author Sad	0.070	0.024	0.293	0.326	0.0008
Author Anxious	0.082	0.035	0.391	0.427	0.0002
Author Happy	0.189	0.150	1.307	1.455	0.0002
Reader Angry	0.095	0.038	0.306	0.350	0.0002
Reader Sad	0.056	0.021	0.316	0.344	0.0018
Reader Anxious	0.082	0.035	0.346	0.384	0.0002
Reader Happy	0.439	0.314	0.941	1.249	0.0002
**Emotional effects (political tweets only)**					
Author Angry	0.205	0.081	1.084	1.173	0.0040
Author Sad	0.170	0.059	0.675	0.731	0.0090
Author Anxious	0.043	0.018	0.804	0.821	0.3762
Author Happy	0.062	0.049	0.523	0.589	0.0336
Reader Angry	0.142	0.057	1.014	1.087	0.0240
Reader Sad	0.088	0.033	0.814	0.858	0.1718
Reader Anxious	0.058	0.025	0.839	0.872	0.2546
Reader Happy	0.111	0.079	0.450	0.548	0.0002
**Political effects**					
Partisanship	0.042	0.009	0.151	0.163	0.0342
Out-group Animosity	0.045	0.006	0.085	0.093	0.0242
In-group Perception (all users)	0.094	0.017	0.060	0.077	0.0002
Out-group Perception (all users)	-0.099	-0.021	-0.108	-0.132	0.0002
In-group Perception (left users)	0.083	0.015	0.056	0.071	0.0002
Out-group Perception (left users)	-0.100	-0.020	-0.097	-0.120	0.0002
In-group Perception (right users)	0.111	0.023	0.072	0.100	0.0002
Out-group Perception (right users)	-0.093	-0.024	-0.145	-0.175	0.0018
**Reader Preference**					
Reader Pref (all tweets)	1.105	0.396	0.507	0.892	0.0002
Reader Pref (political tweets)	0.788	0.283	0.581	0.854	0.0002

Table S43: The effects of our alternative, exploratory ranking of content based on users' stated preferences (SP). The table shows the average treatment effects (standardized and unstandardized), p-values, and p-values for all outcomes. All statistics are calculated as described in SM section S1.3 except we replace the engagement-based timeline with the SP timeline as the treatment of interest.

Outcome	Standardized Effect	Unstandardized Effect	Chron. Mean	Eng. Mean	p-value
**Emotional effects (all tweets)**					
Author Angry	-0.058	-0.023	0.352	0.338	0.0138
Author Sad	-0.011	-0.004	0.293	0.299	0.6243
Author Anxious	0.022	0.009	0.391	0.402	0.2250
Author Happy	0.242	0.192	1.307	1.497	0.0002
Reader Angry	-0.036	-0.015	0.306	0.298	0.0750
Reader Sad	-0.015	-0.006	0.316	0.319	0.4522
Reader Anxious	0.025	0.011	0.346	0.360	0.1466
Reader Happy	0.490	0.350	0.941	1.283	0.0002
**Emotional effects (political tweets only)**					
Author Angry	-0.275	-0.109	1.036	0.938	0.0010
Author Sad	0.015	0.005	0.653	0.657	0.8191
Author Anxious	-0.052	-0.022	0.790	0.761	0.3370
Author Happy	0.145	0.115	0.536	0.668	0.0002
Reader Angry	-0.158	-0.063	0.991	0.942	0.0300
Reader Sad	-0.002	-0.001	0.800	0.810	0.9695
Reader Anxious	0.011	0.005	0.837	0.841	0.8471
Reader Happy	0.195	0.139	0.471	0.628	0.0002
**Political effects**					
Partisanship	-0.166	-0.035	0.151	0.119	0.0002
Out-group Animosity	-0.252	-0.033	0.085	0.054	0.0002
In-group Perception (all users)	-0.004	-0.001	0.060	0.059	0.8345
Out-group Perception (all users)	0.046	0.010	-0.108	-0.101	0.0180
In-group Perception (left users)	-0.021	-0.004	0.056	0.053	0.3364
Out-group Perception (left users)	0.053	0.010	-0.097	-0.091	0.0170
In-group Perception (right users)	0.035	0.007	0.072	0.077	0.2524
Out-group Perception (right users)	0.034	0.009	-0.145	-0.137	0.3616
**Reader Preference**					
Reader Pref (all tweets)	1.117	0.400	0.507	0.896	0.0002
Reader Pref (political tweets)	0.811	0.291	0.585	0.870	0.0002

Table S44: The effects of the SP-OA timeline, the timeline that ranks by users' stated preference and uses the presence of out-group animosity to break ties. The table shows the average treatment effects (standardized and unstandardized), p-values, and p-values for all outcomes. All statistics are calculated as described in SM section S1.3 except we replace the engagement-based timeline with the SP-OA timeline as the treatment of interest.

S4.8 Effects of SP-OA timeline

Ranking by stated preferences (SP) reduced the amount of partisan animosity, relative to the engagement-based timeline. However, this primarily occurred through a reduction in animosity towards the reader's in-group, rather than animosity towards the reader's out-group. In other words, there was an asymmetry in which readers tended to be tolerant of animosity towards their out-group but not their own in-group. The SP timeline had the highest proportion of in-group content and the lowest proportion of out-group content (relative to both the engagement and chronological timeline). Is it possible to satisfy users' stated preferences without inducing in-group bias? To investigate this, we considered a variant of the SP timeline that used the presence of out-group animosity to break ties between tweets. We call this new

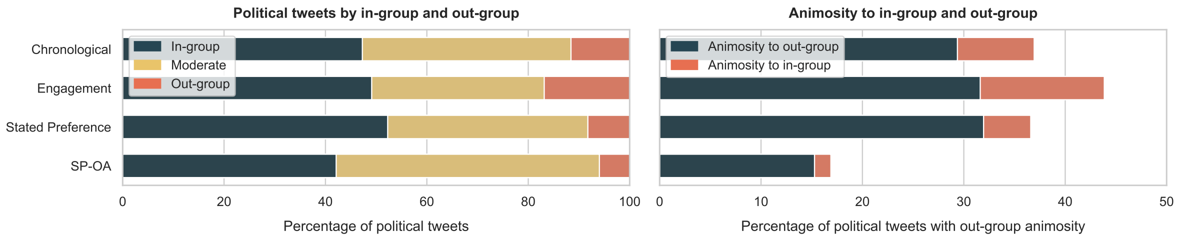

Figure S46: On the left, the graph illustrates the distribution of political tweets in each timeline, categorized by whether they align with the reader's in-group, out-group, or are moderate. Meanwhile, the right graph delineates the proportion of political tweets that express out-group animosity, broken down by whether they target the reader's in-group or out-group. Compared to the engagement and chronological timeline, the stated preference (SP) timeline reduces animosity, but only by reducing animosity towards the reader's *in-group*. In contrast, the SP-OA timeline reduces animosity towards both the reader's in-group and out-group.

timeline the **SP-OA timeline**.

We constructed the SP-OA timeline by adding down-ranking for out-group animosity to the SP timeline. In particular, in the SP timeline, we scored the approximately twenty unique tweets for each user by the users' stated preference for the tweet: 1 = "Yes," 0 = "Indifferent," -1 = "No." In the SP-OA timeline, if a tweet was labeled as having out-group animosity, then we bumped its score down by 0.5 points. The SP-OA timeline for each user consists of their top ten tweets as ranked by this modified score. Note that even if a tweet has out-group animosity, if the user stated that they valued the tweet, it will always be ranked higher than a tweet that they are indifferent to or do not value. Since the presence of out-group animosity is effectively only used to break ties among tweets with the same stated preference, the SP-OA timeline will satisfy users' preferences to the same extent that the regular SP timeline does.

Table S44 shows results for the SP-OA timeline, and Figure S47 compares the effects of all three timelines: the engagement-based timeline, the SP timeline, and the SP-OA timeline. The SP-OA yielded the lowest instances of angry, partisan, and out-group hostile content when stacked against the chronological, engagement, and SP timelines. Furthermore, the SP-OA timeline was the most effective at reducing animosity towards the reader's *out-group* (Figure S46). In the engagement timeline and SP timeline, respectively 34 percent and 33 percent of political tweets contained animosity towards the users' out-group. In contrast, the SP-OA timeline halved this proportion: only 17 percent contained animosity towards the users' out-group.

In conclusion, the SP-OA timeline has high satisfaction of users' stated preferences, mitigates the amplification of divisive content, and does so without reinforcing in-group bias.

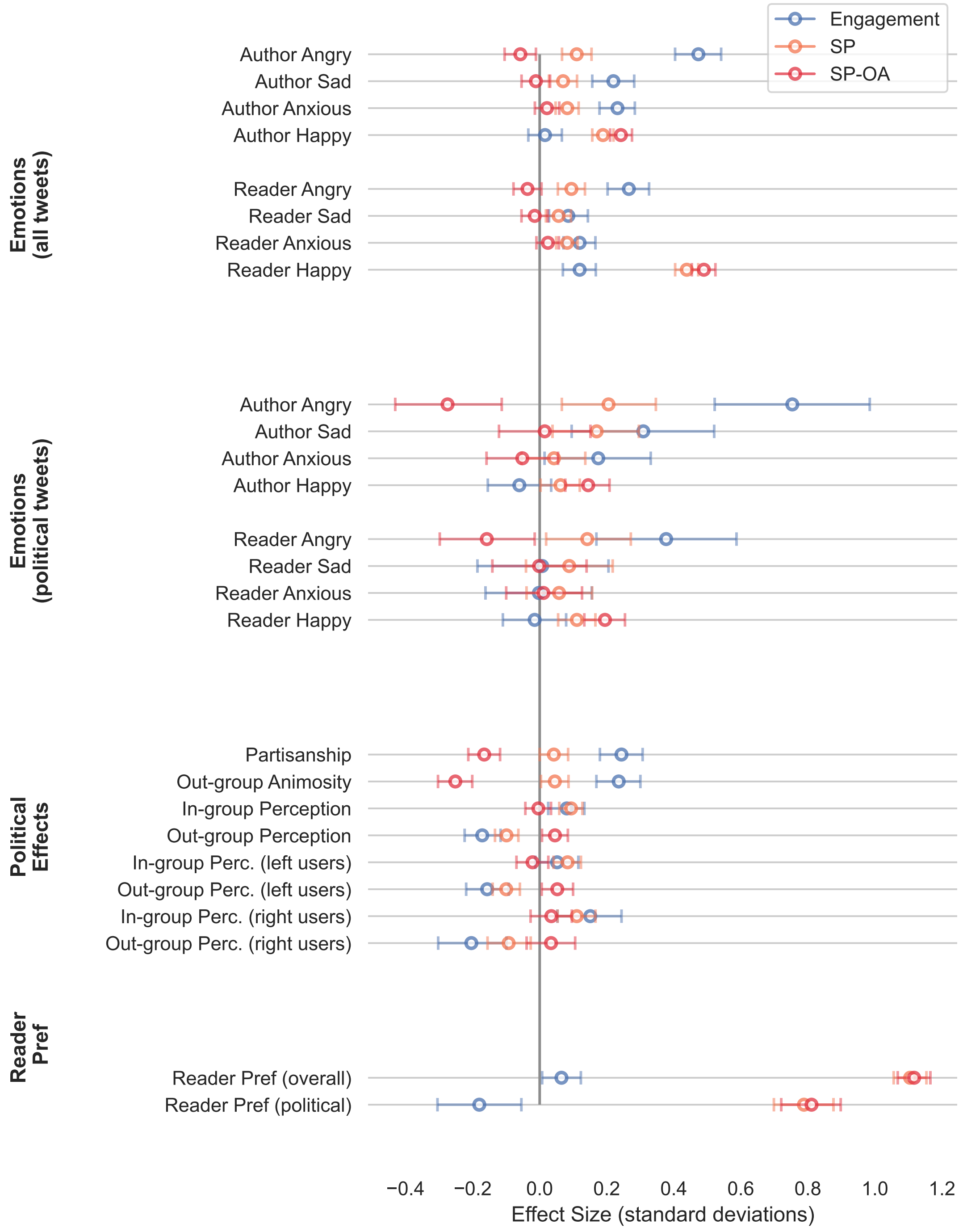

Figure S47: The average treatment effect for all pre-registered outcomes along with their 95% Bootstrap confidence intervals (unadjusted for multiple testing). The effects of three different timelines are shown: (1) Twitter's own engagement-based timeline, (2) our exploratory timeline that ranks based on users' stated preferences (SP), (3) a variant that tie-breaks based on the presence of out-group animosity. The effect sizes for both timelines are relative to the reverse-chronological timeline (the zero line). Average treatment effects are standardized using the standard deviation of outcomes in the chronological timeline (see SM section S1.3 for details).

S5 Survey questionnaires

Not Using Chrome

You must be using Google Chrome on your laptop or desktop (not on your phone) in order to complete this survey. Please try again with Google Chrome.

Screen

Thanks for considering participating in our study!

Our study aims to investigate the effects of Twitter's personalization algorithm.

As part of our study, we will ask you to download a Chrome extension to collect **public** tweets from your own Twitter timeline (no other data will be collected from the Chrome extension). Data collection will only take a couple of minutes and the Chrome extension will automatically uninstall afterwards.

Afterwards, we will show you the tweets and ask you some questions about how you feel about them.

First, we will ask you a few questions to determine your eligibility for the study.

Are you located in the United States?

Yes

No

Are you 18 or older?

Yes

No

About how often do you use or visit Twitter?

Several times a day

About once a day

A few times a week

Once a week or less

How many people do you follow on Twitter?

Less than 50 users

50 users or more

Failed screen

Thank you for considering participating in our study, however based on your answers to the questions we asked, you are not eligible. The survey will end now.

Consent

Consent Form for Investigating Twitter experience

Introduction

My name is Professor Anca Dragan. I am a faculty member at the University of California, Berkeley, in the Electrical Engineering and Computer Science (EECS) Department. I am planning to conduct a research study, which I invite you to take part in.

Purpose

The purpose of this study is to collect data in order to assess the impact of social media algorithms on users.

Procedures

If you agree to be in this study, you will be asked to download a Chrome extension that will collect public tweets from your Twitter timeline. The only data we will collect using the Chrome extension are public tweets, no other data (e.g. account credentials, messages, likes, bookmarks, etc) will be collected. Data collection will only take a few seconds and the Chrome extension will automatically uninstall after collecting data. Afterwards, we will survey you about the tweets collected from your timeline.

Study time: The whole study should take about 30 minutes.

Study location: You will participate online, from the comfort of your current location.

Benefits
There is no direct benefit to you (other than compensation) from participating in this study. We hope that the information gained from the study will help us design better social media algorithms.

Risks/Discomforts
This study represents minimal risk to you. As with all research, there is the risk of an unintended breach of confidentiality. However, we are taking precautions to minimize this risk (see below).

Confidentiality
The data we collect will be stored on password-protected servers. Once the research is complete, we intend to scrub the data of all identifiable information. We will keep only the recorded survey responses, as well as a freshly generated identifier for each subject. The de-identified data will be retained indefinitely for possible use in future research done by ourselves or others. This cleaned dataset may be made public as part of the publishing process. No guarantees can be made regarding the interception of data sent via the Internet by any third parties.

Compensation
We compensate workers based on the estimated duration of completing the study. The study will be prorated to $20/hour for the anticipated duration of completing the study, which is posted for your job on the CloudResearch interface you used to view the job (duration includes reviewing instructions, completing the task, and filling an exit survey). The payment is arranged by CloudResearch via credit to subjects' accounts.

Rights
Participation in research is completely voluntary. You have the right to decline to participate or to withdraw at any point in this study without penalty or loss of benefits to which you are otherwise entitled.

Questions
If you have any questions or concerns about this study, or in case anything goes wrong with the online interface, you can contact Anca Dragan at effects.of.twitter.algorithm@gmail.com.

If you have any questions or concerns about your rights and treatment as a research subject, you may contact the office of UC Berkeley's Committee for the Protection of Human Subjects, at 510-642-7461 or subjects@berkeley.edu.

IRB review:
This study was approved by an IRB review under the CPHS protocol ID number 2021-09-14618

You should save a copy of this consent form for your records.

If you wish to participate in this study, please click the “I consent” button below

I consent

Connect ID

What is your **Cloudresearch Connect ID**?

Chrome extension instructions

We will now have you install a Chrome extension to collect public tweets from your timeline so that we can ask you about them. No other information, including your username or other personally identifying information, will be collected. The data collection will only take a couple of minutes.

Data collection will not work for everyone. As long as you have tried it, we will still pay you 0.75. If it does work for you, and you complete the rest of the survey, you will receive $10.

Follow the steps below:

1. Download the Chrome extension from this link
2. The Chrome extension will open a new tab with instructions. Follow the instructions on screen.
3. When you are done, you will see either a "Success!" or "Error!" page and the Chrome extension will uninstall. You may now move on to the next part of the survey.

Do not proceed until data collection is complete or you will not get the full $10.

If the extension has succeeded, you will see a "Success!" message like the one shown below**.** You can skip to the bottom of this page and proceed to the next page.

Success!

Data collection is complete. The extension will now automatically uninstall. You may now return to the survey.

If you accidentally exited out of the tab or an error occurred, you can restart the process through the following steps:

1. Open the extensions menu in the top right of your browser by clicking the puzzle piece icon.

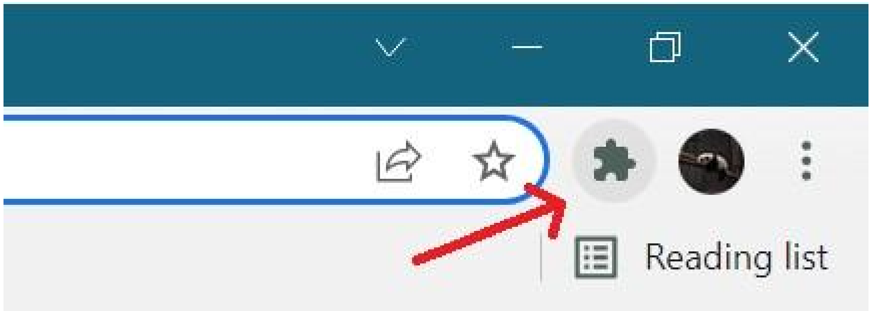

2. Click the bird icon to restart the authorization and data collection process.

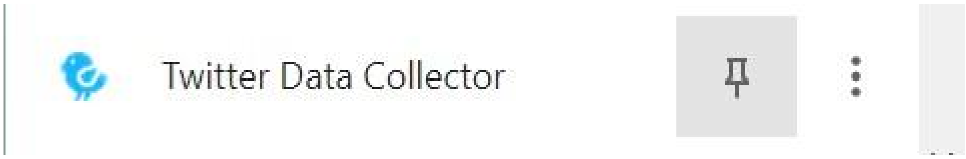

If repeating the steps above still leads you to errors, you can proceed to the next page, and you will still receive the base pay of 0.75$ for having attempted the survey.

I have followed the instructions on the Chrome extension

Outcome success

The previous step completed successfully. For the next part of the study you will be answering questions about individual Tweets we'll show you.

Aim to spend no more than 1-2 minutes per tweet.

The following choices represent the rank of the tweet. This is a hidden question that is used for the Qualtric loop / merge randomization that is used to for randomizing the order of tweets in our outcome survey.

Outcome questions

You have ${e://Field/tweetsLeft} tweets left.

You will be answering questions about the following tweet(s):

Note that there are tweets by both ${lm://Field/4} and ${lm://Field/2}. We will first be asking you questions about **${lm://Field/4}**'s tweet. You can use for ${lm://Field/2}'s tweet for context, but answer the following questions while focusing on ${lm://Field/4}'s tweet.

How is **${lm://Field/4}** feeling in their tweet?

	Not at all	Slightly	Somewhat	Moderately	Extremely
Angry	○	○	○	○	○
Anxious	○	○	○	○	○
Happy	○	○	○	○	○
Sad	○	○	○	○	○

How did **${lm://Field/4}**'s tweet make *you* feel?

	Not at all	Slightly	Somewhat	Moderately	Extremely
Angry	○	○	○	○	○
Anxious	○	○	○	○	○
Happy	○	○	○	○	○
Sad	○	○	○	○	○

Is **${lm://Field/4}**'s tweet about a political or social issue?

Yes

No

How does **${lm://Field/4}**'s tweet lean politically?

	Far left	Left	Moderate	Right	Far right	
-2		-1	0	1		2
						0

How does **${lm://Field/4}**'s tweet make you feel about people or groups on the Left?

	Much worse	Worse	The same as before	Better	Much better	
-2		-1	0	1		2
						0

How does **${lm://Field/4}**'s tweet make you feel about people or groups on the Right?

	Much worse	Worse	The same as before	Better	Much better	
-2		-1	0	1		2
						0

Is **${lm://Field/4}**'s tweet expressing anger, frustration, or hostility towards a person or group on the Left?

Yes

No

Is **${lm://Field/4}**'s tweet expressing anger, frustration, or hostility towards a person or group on the Right?

Yes

No

When you use Twitter, do you want to be shown tweets like **${lm://Field/4}**'s tweet?

Yes

No

Indifferent

Next, we will be asking you questions about **${lm://Field/2}**'s tweet.

How is **${lm://Field/2}** feeling in their tweet?

	Not at all	Slightly	Somewhat	Moderately	Extremely
Angry	◯	◯	◯	◯	◯
Anxious	◯	◯	◯	◯	◯
Happy	◯	◯	◯	◯	◯
Sad	◯	◯	◯	◯	◯

How did **${lm://Field/2}**'s tweet make *you* feel?

	Not at all	Slightly	Somewhat	Moderately	Extremely
Angry	◯	◯	◯	◯	◯
Anxious	◯	◯	◯	◯	◯
Happy	◯	◯	◯	◯	◯
Sad	◯	◯	◯	◯	◯

Is **${lm://Field/2}**'s tweet about a political or social issue?

Yes

No

How does **${lm://Field/2}**'s tweet lean politically?

Far left	Left	Moderate	Right	Far right

-2 -1 0 1 2

0

How does **${lm://Field/2}**'s tweet make you feel about people or groups on the Left?

Much worse	Worse	The same as before	Better	Much better
-2	-1	0	1	2

0

How does **${lm://Field/2}**'s tweet make you feel about people or groups on the Right?

Much worse	Worse	The same as before	Better	Much better
-2	-1	0	1	2

0

Is **${lm://Field/2}**'s tweet expressing anger, frustration, or hostility towards a person or group on the Left?

Not at all

Slightly

Somewhat

Moderately

Extremely

Is **${lm://Field/2}**'s tweet expressing anger, frustration, or hostility towards a person or group on the Right?

Not at all

Slightly

Somewhat

Moderately

Extremely

When you use Twitter, do you want to be shown tweets like **${lm://Field/2}**'s tweet?

Yes

No

Indifferent

Overall Content Questions

What were the tweets we showed you today predominantly about? Select a maximum of two.

News

Politics

Work

Entertainment

Hobbies

Other

Data collection failed

Sorry, an error occurred and we were unable to collect your data. This is a known issue that happens with a small portion of the participants.

You should fill out the form at the link below to be paid *a base rate of compensation of $0.75:* https://forms.gle/CJNtmYVbMhHaeNTw7

Please advance to the next page to complete this survey and then "Return"/"Withdraw from" the study in CloudResearch Connect. If you submit a completion code, your HIT will be rejected.

If you have any feedback, share it with us on CloudResearch Connect.

Additional background information

To end, we will ask some basic questions about your background.

In which state do you currently reside?

What gender do you identify as?

Woman

Non-binary

Man

Other

How old are you?

18-24 years old

25-34 years old

35-44 years old

45-54 years old

55-64 years old

65-74 years old

75 years or older

Are you Spanish, Hispanic, or Latino or none of these (select all that apply).

Spanish

Hispanic

Latino

None of these

Choose one or more races that you consider yourself to be:

White

Black or African American

American Indian or Alaska Native

Asian

Native Hawaiian or Pacific Islander

Other

What is the highest level of schooling that you have completed?

Some high school

High school graduate

Associate degree

Bachelor's degree

Master's degree or above

Prefer not to answer

Other

In politics today, do you consider yourself a:

Republican

Democrat

Independent

Something else

As of today do you lean more to...

The Republican Party

The Democratic Party

How do you identify politically?

Far left

Left

Moderate

Right

Far right

Other

Which way do you lean politically?

Towards the Left

Towards the Right

What is your annual household income?

Less than $25,000

$25,000 - $50,000

$50,000 - $100,000

$100,000 - $200,000

More than $200,000

Prefer not to say

Why do you use Twitter? ***Select all that apply***.

Entertainment

A way to stay informed

A way to express my opinions

Keeping me connected to other people

Lets me see different points of view

It's useful for my job or school

What would you say is the ***main*** reason you use Twitter?

Entertainment

A way to stay informed

A way to express my opinions

Keeping me connected to other people

Lets me see different points of view

It's useful for my job or school

Thank you! **To complete the survey, click next and you will be redirected back to Cloudresearch.**

Powered by Qualtrics
